# A Scalable Stereoselective Synthesis of Polysubstituted Housanes

**DOI:** 10.1021/acs.orglett.6c01751

**Published:** 2026-06-04

**Authors:** Valentin V. Veselinov, Ayan Dasgupta, Andreas Pielmeier, Adrián López-Francés, Hafdís Haraldsdóttir, Kirsten E. Christensen, Edward A. Anderson

**Affiliations:** Chemistry Research Laboratory, Department of Chemistry, 6396 University of Oxford, 12 Mansfield Road, Oxford, OX1 3TA, U.K.

## Abstract

Small ring bicyclic carbocycles are valuable building blocks in medicinal chemistry due to their rigid structures and useful physicochemical properties. Bicyclo[2.1.0]­pentanes (housanes) have received relatively little attention, and methods for their late-stage diversification remain limited. Here we report a straightforward directed metalation approach enabling the synthesis of di- and trisubstituted housanes with excellent regio- and diastereoselectivity via sequential bridgehead and cyclopropane bridge functionalization. We also explore housanes as isosteres of *ortho*-substituted benzene rings using computational and X-ray crystallographic analysis, and describe a stereospecific boron-mediated 1,2-metalate rearrangement that affords a highly substituted cyclopentaneboronic ester. Finally, we disclose an enantioselective synthesis of a housane, enabling access to enantioenriched polysubstituted housane scaffolds.

## Graphical abstract

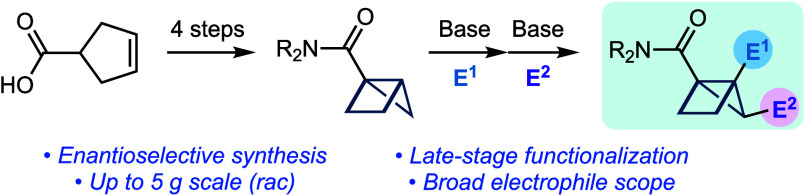

Small ring bicyclic hydrocarbons such as bicyclo[1.1.1]­pentanes (BCPs), bicyclo[2.1.1]­hexanes (BCHs) and bicyclo[3.1.1]­heptanes (BCHeps) have blossomed in popularity in recent years due to their potential utility as bioisosteres of benzene rings in drug design (Figure 1a).^1^ This is due to the superior pharmacokinetic and physicochemical properties of these saturated cores compared to their aromatic counterparts, and their well-defined substituent exit vectors, which in the case of BCPs and BCHeps accurately mimic the geometries of *para-* ^2^ and *meta-*substituted^3^ aromatic rings, respectively. The most common routes to such motifs involve either ring-opening reactions of [n.1.1]­propellanes,^2,3^ or one-, two- or three-atom insertions into bicyclo[1.1.0]­butanes (BCBs),^4^ both of which are generally considered to be facilitated by relief of ring-strain on cleavage of the central C–C bonds.^5^

**Figure 1:**
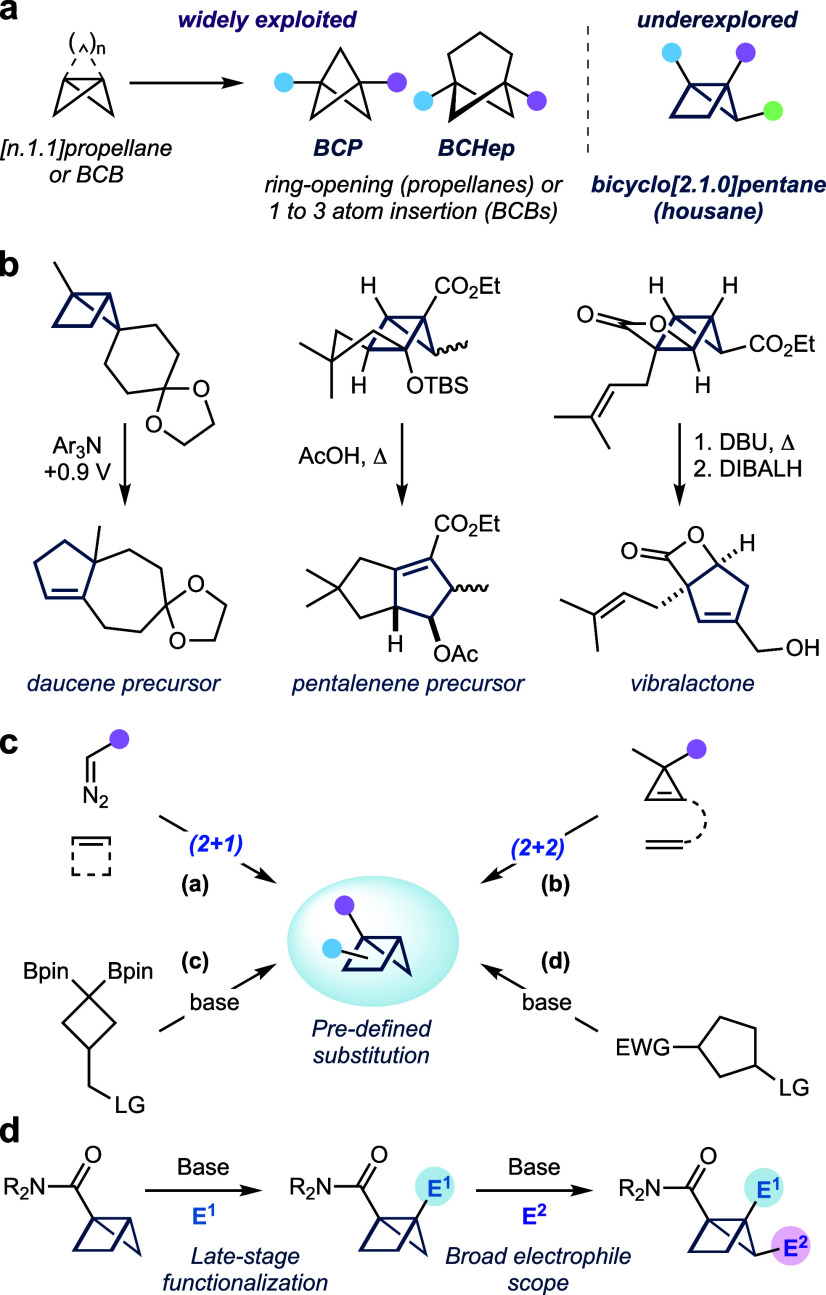
**a** Structures of widely explored Bicyclo­[n.1.1]­alkanes (BCPs and BHeps). **b** Applications of housanes. **c** Existing routes for the functionalized housanes employ ’early stage’ substituent installation. **d** This work, late-stage functionalization of housane via regio- and stereoselective amide-directed metalation.

Bicyclo­[2.1.0]­pentanes, also known as ’housanes’, are underexplored homologues of BCBs. Housanes are kinetically stable compared to BCBs due to the presence of only one cyclopropane ring fused to their central C–C bond, which reduces kinetic lability toward ring-opening.^6^ Nevertheless, housanes are versatile molecules capable of undergoing a variety of transformations, for instance having been deployed as precursors to cyclopentenes in target-oriented synthesis

(Figure 1b). Examples include an electrosynthetic oxidative housane rearrangement toward daucene,^7^ acid-promoted ring-opening in a formal synthesis of pentalenene,^8^ and base-mediated fragmentation toward vibralactone.^9^ In addition, sulfone-substituted housanes have been shown to exhibit ’strain-release’ alkylation reactivity to form disubstituted cyclopentanes,^10^ albeit such structures are uniformly unsubstituted at one of the bridgehead position.

A number of methods have recently been developed that overcome the limitations of classical approaches^11^ to access housanes featuring predefined substituents (Figure 1c). Strategies include (a) (2 + 1) cyclopropanations of alkenes^12^ and cyclobutenes;^13^ (b) (2 + 2) photocatalyzed cycloadditions of cyclopropenes or 1,4-dienes^14^ and related stepwise approaches;^15^ (c) cyclization reactions of 1,1-diborylcyclobutanes onto pendent electrophiles.^16^ The most scalable tactic developed to date (d) involves the transannular cyclization of cyclopentane rings equipped with anion-stabilizing groups (esters, sulfones, etc.) and suitable leaving groups.^10,17^ As noted, all of these methods intrinsically predefine the nature of the substituents on the housane framework, and broader applications of housanes are therefore somewhat limited by a paucity of methods for late-stage functionalization. Similarly, enantioselective syntheses of housanes remain relatively underdeveloped, with only a limited number of asymmetric strategies reported, which exhibit constraints in scope or selectivity.^10,14^a,^16^a

We previously reported methods for the sequential introduction of substituents at the bridgehead and bridge positions of BCBs via directed metalation/functionalization,^13^b,^18^ and questioned whether a similar approach might be used to decorate a monosubstituted housane framework. Here we disclose the realization of this straightforward method for the functionalization of housanes with a wide variety of groups. An enantioselective route for synthesis of the housane framework is also described.

Housane **1** (Figure 2) was first accessed in four steps from commercially available cyclopentene-3-carboxylic acid by modification of the elegant chemistry described by Gyrgorenko et al.^17b^ Thus, formation of the diisopropylamide was followed by alkene hydroboration using BH_3_·THF, which delivered alcohol **2** as a single diastereomer after oxidative workup.^19^ Treatment with benzenesulfonyl chloride converted **2** to **3** with retention of configuration, a process that presumably benefits from anchimeric assistance by the amide carbonyl.^19^ Finally, amide enolization using LiHMDS was followed by cyclization to form housane **1**. This sequence, which proceeded in 32% overall yield from the carboxylic acid starting material, could be readily conducted on multigram scale.

**Figure 2:**
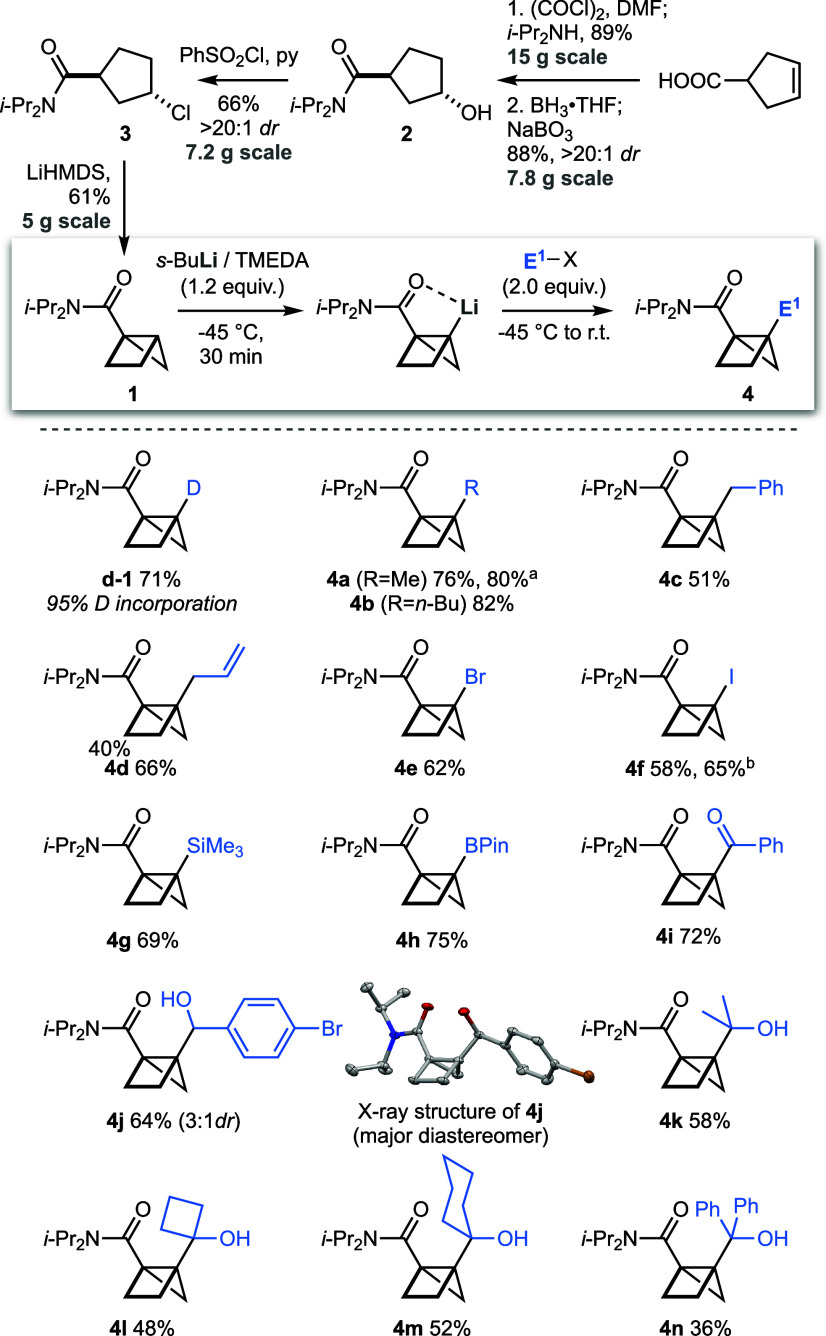
Substrate scope for the bridgehead functionalization of housane **1.** All reactions were carried out on a 0.1–0.2 mmol scale, ^a^ 1.5 mmol scale. ^b^ 0.72 mmol scale. Yields reported are isolated yields.

Targeting conditions to achieve deprotonation at the bridgehead position of **1**, we found that treatment with bases such as *t-* or *s-*BuLi at – 78 °C followed by quenching with CD_3_OD resulted in no deuterium incorporation at the bridgehead position. However, by conducting the reaction using *s*- BuLi at – 45 °C in conjunction with an equivalent quantity of TMEDA, 93% deuterium incorporation was observed, exclusively at the bridgehead position (**d-1**, Figure 2).

The scope of bridgehead lithiation/electrophilic functionalization was then assessed under these optimized conditions. Carbon electrophiles such as alkyl, benzyl and allyl halides were readily accommodated (**4a**-**d**). Bridgehead heteroatom installation proved possible using *N-*halosuccinimides, chlorotrimethylsilane, and isopropanol-pinacolboronic ester (**4e**-**h**). The reaction also proved amenable to the use of carbonyl-based electrophiles such as Weinreb amides (**4i**), aldehydes (**4j**), and ketones (**4k**-**n**). Cyclobutyl product **4l** illustrates the potential to install other valuable small ring motifs onto the housane scaffold. Crystallization of **4i**, **4j** and **4k** enabled confirmation of the structures by single-crystal X-ray diffraction analysis^20^ and offered insight into housane geometry (see below). We were also able to scale up the reactions to 0.72 (**4f**) and 1.5 mmol (**4a**) scale with no detriment to the yield.

With successful bridgehead lithiation/functionalization achieved, we next explored whether a second, regioselective lithiation of the cyclopropyl bridge C–H bond could be achieved. For this purpose, we selected housane **4a**, bearing a methyl group at the bridgehead position. Under optimized bridgehead lithiation conditions (*s*-BuLi/TMEDA, Figure 3), 91% deuterium incorporation was observed on quenching with MeOD, demonstrating excellent regiocontrol for *exo* lithiation, and complete preference for cyclopropyl over cyclobutyl C–H activation. These selectivities presumably arise from the greater acidity of the cyclopropyl C–H bond, and the direction of lithiation *syn* to the amide group, reinforced by the *cis*-fused bicyclic nature of the housane skeleton. The reaction of lithiated **4a** with various electrophiles was then examined, which also proceeded with exceptional diastereoselectivity in all cases (**5a**-**h**, > 20:1 *dr*, 57–89% yield). Once again, a variety of electrophiles were accommodated, including alkyl halides (**5a**-**c**), heteroatoms (**5d** and **5e**) and carbonyls (**5f** and **5g**), and a boronic ester (**5h**, 71%). Collectively, this sequenced metalation strategy thus enables the synthesis of a wide variety of polysubstituted housanes with complete regio and stereocontrol. A one-pot sequential lithiation/electrophilic difunctionalization was explored, where **1** was converted to **4a**, and then *in situ* to **5e** (by sequential deprotonations and use of MeI and TMSCl as electrophiles, Scheme 1a). **5e** was isolated in 38% yield (compared to 48% yield over two discrete steps), demonstrating the potential to carry out one-pot syntheses of trisubstituted housanes.

**Figure 3:**
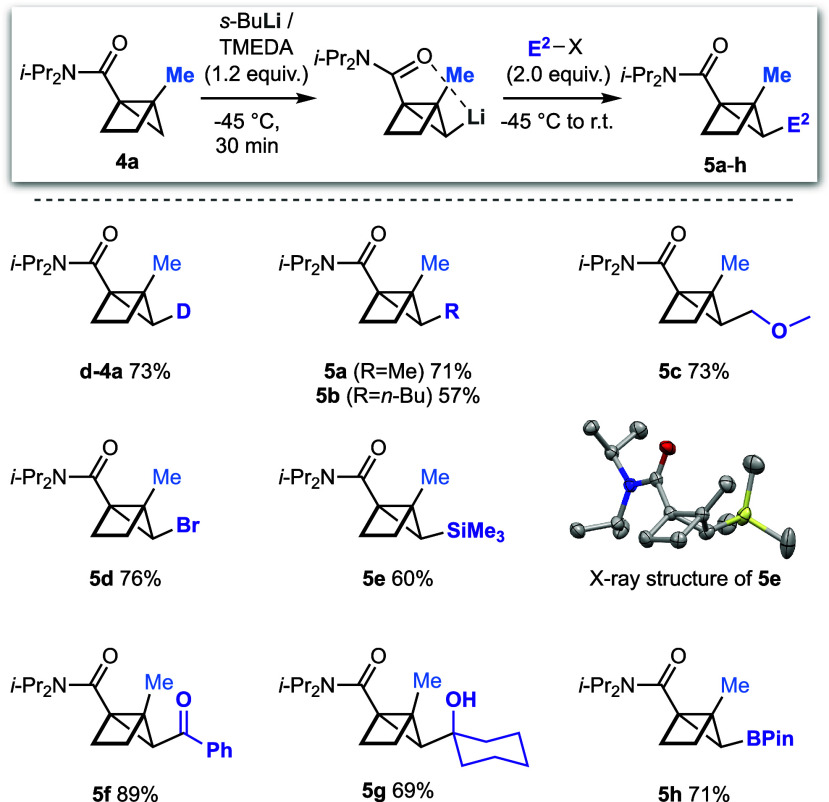
Substrate scope for the cyclopropane bridge functionalization of housane **4a**. All reactions were carried out on a 0.1–0.2 mmol scale, and the yields reported are isolated yields.

**Scheme 1:**
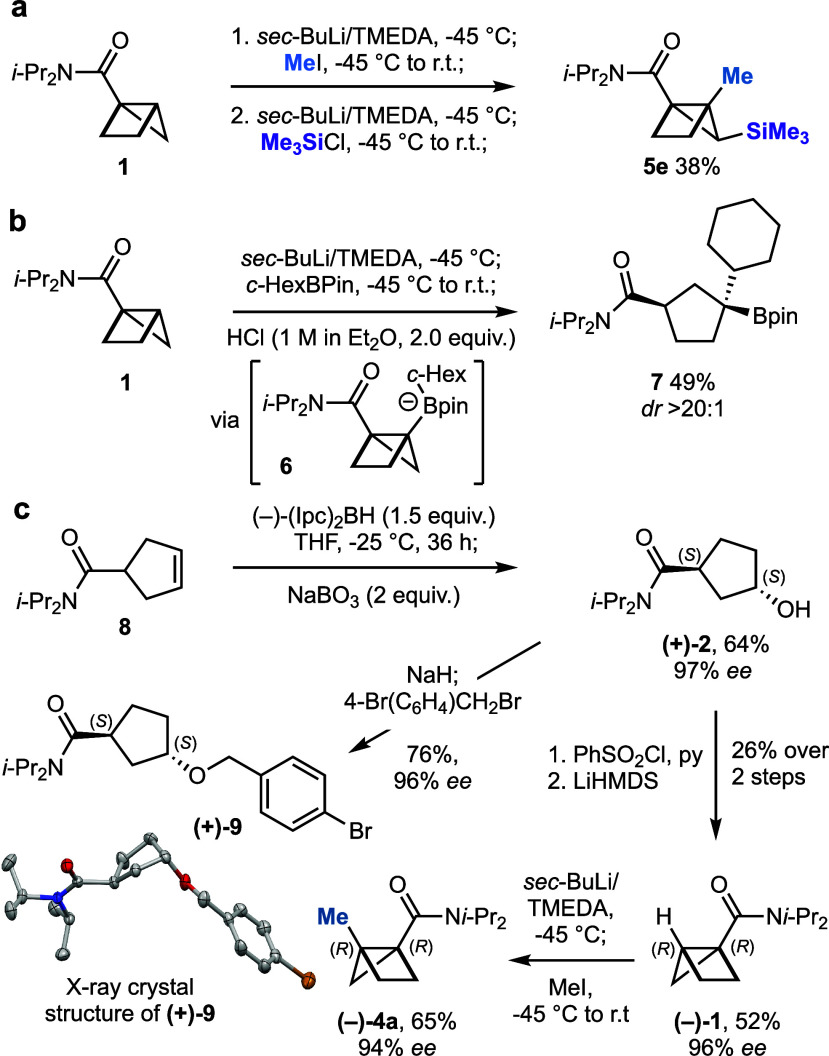
Further Housane Transformations: a. One-Pot Electrophilic Functionalization; b. Boron 1,2-Metallate Rearrangement; c. Enantioselective Synthesis of (−)-**1** and (−)-**4**

Bridgehead boronic esters of BCBs have been shown to undergo 1,2-metalate rearrangements that cleave the central bond of the BCB upon treatment with electrophiles,^21^ and we questioned whether the less reactive inter bridgehead C–C bond of a housane might also be subject to such chemistry. To test this, lithiated **1** was treated with cyclohexyl pinacolboronic ester, affording a presumed boron ate-complex **6**

(Scheme 1b). *In situ* treatment of this complex with ethereal HCl successfully triggered the 1,2-metalate rearrangement, affording a single diastereomer of the cyclopentylboronic ester **7**. The formation of this product can be explained by a stereospecific migration/ring-opening step, which is consistent with the findings of Aggarwal and co-workers.^21^

Unlike BCBs, housanes are intrinsically chiral, and thus offer opportunities for enantioselective synthesis. We therefore aimed to develop a straightforward route to access housanes **1** (and by inference, their di- or trisubstituted derivatives) in enantioenriched form (Scheme 1c). Toward this end, we explored an enantioselective hydroboration of prochiral alkene **8**. Pleasingly, enantioselective hydroboration using (−)-Ipc_2_BH followed by oxidation afforded cyclopentanol **(+)-2** as a single diastereomer in 64% yield and 97% *ee*. The absolute stereochemistry of **(+)-2** was established by its derivatization as the *p-*bromobenzyl ether **(+)-9**, single crystal X-ray diffraction analysis^20^ of which allowed assignment of relative and absolute stereochemistry. Conversion of **(+)-2** to the enantioenriched housane **(−)-1** proceeded as before, with almost no erosion of enantioselectivity (96% *ee*). Finally, we demonstrated the potential to use this route to access enantioenriched polysubstituted housanes: methylation at the bridgehead position of **(−)-1** under the standard conditions afforded **(−)-4a** in 64% yield and 94% *ee*, demonstrating the stereochemical stability of the housane framework.

Housanes have previously been proposed as bioisosteres for cyclopentane rings.^17b^ However, we recognized that the geometries of the substituents might also resemble those of an *ortho* disubstituted aromatic, for which an accurate, tunable bioisostere platform remains elusive.^22^ The synthesis of crystalline di- and trisubstituted housanes enabled comparison of their geometries with those of equivalent *ortho-*substituted arenes (Figure 4). Two sets of exit vectors were considered (see ’model’), namely the bridgehead substituents (gray/blue spheres, exit vector angle α_1_°, dihedral angle φ_1_°, atomic separation r_1_), and the bridgehead/cyclopropane bridge substituents (purple/blue spheres, exit vector angle α_2_°, dihedral angle φ_2_°, atomic separation r_2_). Housanes **4i** and **5e** were used, with the values determined from X-ray crystallographic analysis compared with those computed for **4i** and **5e** at the ωB97X-D/def2-TZVPP level of theory, as well as equivalent calculations for the corresponding *ortho-*disubstituted arenes. Comparison of the angles between calculated (housane and arene) and solid state structures showed excellent agreement, with values for angles α_1_ and φ_1_ being within 9–17° and 0–11° respectively between the three systems. The larger discrepancy of 17° between calculated and experimental values may reflect effects of crystal packing in **5e**). Similar agreement was noted for the bridgehead–bridge angles α_2_ and φ_2_ in **5e**, which exhibited difference ranges of 8° and 3° respectively. The separation of the substituent atoms directly bonded to the housane also exhibited good similarity to that of the *ortho* arene (2.90–3.52 Å ± 0.35–0.5 Å) for both the bridgehead and bridgehead–bridge pairs. These results indicate that housanes and their *ortho-*phenyl analogues are indeed geometrically isosteric. It is possible that other underrepresented structures, such as *cis-*alkenes, may also be mimicked by the housane system.^23^

**Figure 4:**
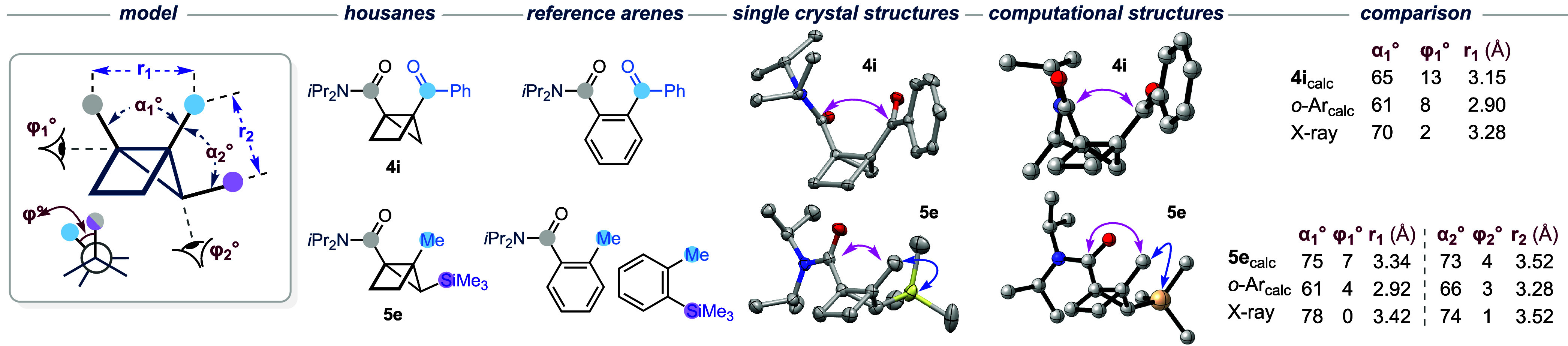
Comparison of exit vector angles between the parent arenes (*o*-A*r*_calc_), single-crystal X-ray structures of **4i** and **5e**, and the corresponding computed structures for housane (**4i**_calc_ and **5e**_calc_). Substituent exit vector angles are defined as α_1_° and α_2_°, and out-of-plane exit vector angles (dihedral angles) are defined as φ_1_° and φ_2_°. Distances in Å between substituent atoms are labeled r_1_ and r_2_. Calculations were carried out at the ωB97X-D/def2-TZVPP level of theory.

In conclusion, housanes are underexploited as small ring building blocks in organic synthesis, in part due to the difficulty of installing a diversity of substituents on the bicyclic framework. Using a straightforward directed lithiation approach followed by electrophilic trapping of the intermediate organolithium, a wide variety of polysubstituted housanes can now be accessed. Challenges remain, including the successful derivatization^24^ or variation of the amide directing group, and the validation of housanes as bioisosteres of *ortho*-arenes in a biological context. However, the close agreement between crystallographic and computational geometries, along with asymmetric housane synthesis and downstream ring functionalization, is highly suggestive of new possibilities for the applications of housanes in synthesis and biology.

## Supplementary Material



## Data Availability

The data underlying this study are available in the published article and its Supporting Information.

## Notes

Experimental procedures, copies of NMR spectra, computational details, and X-ray crystallographic data (PDF)
