# Supplementary material for: A Scalable Stereoselective Synthesis of Polysubstituted Housanes
Source: Org Lett. 2026 Jun 4;28(23):7279–84. doi: 10.1021/acs.orglett.6c01751 (PMC13270642; doi:10.1021/acs.orglett.6c01751)
Supplement: Supplementary file 1 [file ol6c01751_si_001.pdf]

A Scalable Stereoselective Synthesis of Polysubstituted Housanes

Valentin V. Veselinov,‡ Ayan Dasgupta,‡ Andreas Pielmeier,‡ Adrián López-Francés, Hafdís Haraldsdóttir, Kirsten E. Christensen and Edward A. Anderson*

‡ These authors contributed equally

Chemistry Research Laboratory, Department of Chemistry, University of Oxford, 12 Mansfield Road, Oxford, OX1 3TA, U.K.

*Corresponding author. Email: edward.anderson@chem.ox.ac.uk

Supporting Information

Table of Contents

1. General experimental methods 2

2. Synthesis of housane **(±)-1** 3

3. Optimisation of the metallation protocol: Deuterium quench 8

3.1 Deuterium installation at the bridgehead position of bicyclo[2.1.0]pentane 8

3.2 Deuterium installation at the bridge position 9

4. General procedures 10

5. Experimental procedures and characterisation 11

6. Enantioselective synthesis of **(–)-1** 26

7. Unsuccessful attempts 34

8. NMR spectra 35

9. Computational details 72

9.1 Comparative geometric analysis of computed and X-ray structures 72

9.2 Structural superposition of housanes and corresponding ortho-phenyl moieties 75

9.3 Cartesian coordinates 76

10. Crystallographic data 81

10.1 Solid-state structures 82

10.2 X-ray refinement data 85

11. References 91

1. General experimental methods

NMR Spectroscopy: ^{1}H and ^{13}C NMR spectra were recorded on Bruker Avance III HD 400, Bruker Avance III HD 500, Bruker NEO 600 (broadband helium cryoprobe) and Bruker Avance III 700 spectrometers. Chemical shifts (δ) are quoted in parts per million (ppm) to the nearest 0.01 for ^{1}H and 0.1 for ^{13}C (unless ambiguous). ^{1}H NMR spectra were recorded using an internal deuterium lock for residual $CDCl_3$ (δ = 7.26) or C_6D_6 (δ = 7.16); ^{13}C NMR spectra were recorded using an internal deuterium lock for residual $CDCl_3$ (δ = 77.16) or C_6D_6 (δ = 128.06). Assignments were made based on unambiguous chemical shifts, coupling patterns, COSY, HSQC and/or NOESY experiments. Peak multiplicities used are singlet (s), doublet (d), triplet (t), triplet (t), quintet (p), multiplet (m), broad (br.). Coupling constants (*J*) are reported to the nearest 0.1 Hz. In the case of the di-*iso*-propylamide motif, in some cases the ^{13}C NMR signals of the diastereotopic CH_3 and CH protons are overlapping and/or broad. Spectra are therefore reported as observed.

Mass Spectrometry: High-resolution mass spectra were recorded on a Thermo Exactive High-Resolution Orbitrap FTMS or Waters BioAccord System with Acquity Premier. High-resolution values are calculated to 4 decimal places from the molecular formula, and all values are within a tolerance of 5 ppm or 3 mmu.

Infrared Spectroscopy: Infrared spectra were obtained from evaporation from chloroform or benzene as solvent on a Bruker Tensor 27 FT-IR spectrometer, as a thin film on a diamond ATR module. Wavelengths of maximum absorbance (v_{max}) are quoted in cm^{-1}, with only characteristic IR absorption data provided for each novel compound.

Chromatography: Normal phase flash column chromatography was performed on silica gel obtained from Merck (Silica gel Si 60, 0.040-0.063 mm) under a positive pressure of nitrogen, using the stated solvent system. Thin-layer chromatography was performed on pre-coated aluminium-backed plates (Merck Kieselgel 60 F254 plates) with visualization by ultraviolet light (254 nm) and/or by a suitable stain. Retention factors (R_f) are reported with the solvent system in parentheses. High-performance liquid chromatography (HPLC) was carried out using a Phenomenex Lux i-Amylose-1 (250 mm x 4.6 mm ID) or DAICEL CHIRALPAK IA or IC (250 mm x 4.6 mm) column (wavelength: 210 nm or 222 nm) with purified material on an Agilent 1200 series under UV-vis detection.

Polarimetry: Optical rotations were recorded using a Perkin Elmer 241 Polarimeter, using the sodium D line (589 nm), with a path length of 1 dm at 20 °C.

Materials/procedures: All air- or moisture-sensitive reactions were carried out in anhydrous solvents in heat gun-dried glassware under an inert atmosphere of nitrogen. Reactions were performed at room temperature (rt, ~23 °C) unless otherwise stated. 0 °C was maintained using an ice-water bath, -25 °C using a Huber TC50E cooler, -45 °C using an acetonitrile-dry ice bath, -78 °C using an acetone-dry ice bath. Heating was performed using a sand bath and maintained with an electric hot plate. Dry solvents were collected fresh from an mBraun SPS-800 solvent purification system, having been passed through anhydrous alumina columns. Reagents were directly used as supplied by commercial suppliers.

2. Synthesis of Housane (±)-1

***N,N*-diisopropylcyclopent-3-ene-1-carboxamide, 8**

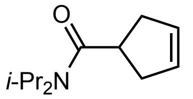

Cyclopent-3-ene-1-carboxylic acid (15.0 g, 134 mmol, 1.0 equiv.) was dissolved in CH_2Cl_2 (75 mL), and DMF (15 drops) was added. The mixture was cooled to 0 °C and oxalyl chloride (14.0 mL, 161 mmol, 1.2 equiv.) was added dropwise at 0 °C. The mixture was stirred at ambient temperature (23 °C) overnight, then the solvent was evaporated *in vacuo* (to 200 mbar at 30 °C). The residue was dissolved in CH_2Cl_2 (75 mL), the solution was cooled to 0 °C, and diisopropylamine (56.3 mL, 401 mmol, 3.0 equiv.) was added dropwise at 0 °C. The reaction mixture was stirred at ambient temperature (23 °C) overnight, then washed with NH_4Cl (sat., aq., 2 × 30 mL) and brine (30 mL), dried over $MgSO_4$, filtered and concentrated *in vacuo*. The residue was purified by flash column chromatography using pentane/ethyl acetate (0→10% v/v) to afford *N,N*-diisopropylcyclopent-3-ene-1-carboxamide **8** (23.3 g, 119 mmol, 89%) as a white solid.

$\mathbf{R}_f$ = 0.5 (pentane/ethyl acetate 9:1, v/v); **^{1}H NMR** (400 MHz, $CDCl_3$) δ_H 5.58 (s, 2H), 4.03 (p, *J* = 6.7, 1H), 3.44 (br. s, 2H), 3.20 (tt, *J* = 9.5, 6.8 Hz, 1H), 2.80–2.57 (m, 2H), 2.54–2.42 (m, 2H), 1.32 (d, *J* = 6.8 Hz, 6H), 1.16 (d, *J* = 6.7, 6H,); **^{13}C NMR** (101 MHz, $CDCl_3$) δ_C 174.2, 128.8, 48.0, 45.6, 40.8, 36.8, 21.2, 20.7; **IR** ν_{max} (cm^{-1}): 3056, 2967,

1626, 1440, 1308; **HRMS** (ESI+) calculated for $C_{12}H_{22}NO$ $[M+H]^+$ 196.1696, found 196.1695.

(1*S,3*S**)-3-hydroxy-*N*,*N*-diisopropylcyclopentane-1-carboxamide, 2**

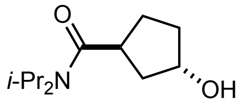

To a THF solution (30 mL) of *N,N*-diisopropylcyclopent-3-ene-1-carboxamide (7.80 g, 39.9 mmol, 1.0 equiv.) at 0 °C was added 1.0 M BH_3·THF complex (28.0 mL, 28.0 mmol, 0.70 equiv.) dropwise. The solution was stirred at ambient temperature (23 °C) for 2 h. Water (30 mL) was added slowly dropwise, followed by the addition of sodium perborate tetrahydrate (6.14 g, 39.9 mmol, 1.0 equiv.). The mixture was stirred overnight at ambient temperature. Saturated brine (25 mL) solution was added to the reaction mixture and the organic compounds were extracted with ethyl acetate (3 x 30 mL). The combined organic extracts were washed with brine (100 mL), dried over $MgSO_4$, filtered and concentrated *in vacuo.* The residue was purified by flash column chromatography on silica gel using pentane/ethyl acetate (3:7, v/v) to afford **2** (7.50 g, 35.2 mmol, 88%) as a clear viscous liquid.

$\mathbf{R}_f$ = 0.2 (pentane/ethyl acetate 3:7, v/v); **^{1}H NMR** (600 MHz, $CDCl_3$) δ_H 4.57–4.36 (m, 1H), 4.10 (br. s, 1H), 3.6–3.36 (m, 1H), 3.17 (p, *J* = 8.3 Hz, 1H), 2.09 (ddd, *J* = 14.0, 9.1, 5.2 Hz, 1H), 2.06–1.96 (m, 2H), 1.84–1.75 (m, 2H), 1.68–1.59 (m, 1H), 1.36 (d, *J* = 6.8 Hz, 6H), 1.18 (dd, *J* = 6.8, 3.5 Hz, 6H); **^{13}C NMR** (151 MHz, $CDCl_3$) δ_C 174.4, 74.0, 48.0, 45.7, 40.7, 39.6, 35.2, 28.0, 21.4, 20.8, 20.7; **IR** ν_{max} (cm^{-1}): 3398 (br), 2968, 1621, 1430, 1372; **HRMS** (ESI+): calculated for $C_{12}H_{23}NO_2$ $[M+H]^+$ 214.1802, found 214.1801.

(1*S,3*S**)-3-chloro-*N*,*N*-diisopropylcyclopentane-1-carboxamide, 3**

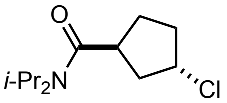

A solution of **2** (7.20 g, 33.8 mmol, 1.0 equiv.) in pyridine (45 mL) was cooled to 0 °C, and $PhSO_2Cl$ (5.60 mL, 43.9 mmol, 1.3 equiv.) was added dropwise. The mixture was stirred at ambient temperature (23 °C) for 8 h, then it was diluted with CH_2Cl_2 (50 mL), washed with NH_4Cl (sat., aq., 3 × 50 mL) and brine (30 mL), dried over $MgSO_4$, filtered and concentrated *in vacuo.* The residue was purified by flash column chromatography

on silica gel using pentane/diethyl ether (8:2, v/v) to afford **3** (5.20 g, 22.4 mmol, 66%) as a yellow liquid.

R*f* = 0.5 (pentane/diethyl ether 7:3, v/v); **^{1}H NMR** (600 MHz, $CDCl_3$) δ_H 4.57 (tt, *J* = 5.3, 2.6 Hz, 1H), 4.07 (br. s, 1H), 3.52 (br. s, 1H), 3.31–3.24 (m, 1H), 2.41 (ddd, *J* = 14.1, 8.8, 5.4 Hz, 1H), 2.27–2.12 (m, 2H), 2.12–2.05 (m, 1H), 2.03–1.95 (m, 1H), 1.90–1.81 (m, 1H), 1.35 (d, *J* = 6.8 Hz, 6H), 1.23 (d, *J* = 6.6 Hz, 6H), 1.22 (d, *J* = 6.9 Hz, 6H); ^{13}C NMR (151 MHz, $CDCl_3$) δ_C 173.8, 62.9, 48.1, 45.8, 41.09, 40.47, 36.7, 27.9, 21.4, 20.8; **IR** ν_{max} (cm^{-1}): 2966, 1646, 1434, 1331, 1161; HRMS (ESI+): calculated for $C_{12}H_{23}{}^{35}ClNO$ $[M+H]^+$: 232.1463, found 232.1458.

Stereochemical analysis

The stereochemistry of product **3** (substitution with retention of configuration) was established retrospectively through ring opening reaction of housane **1** on treatment with HCl, which afforded a mixture of diastereoisomers **3** (identical ^{1}H NMR specturm to that obtained above) and **3'**:

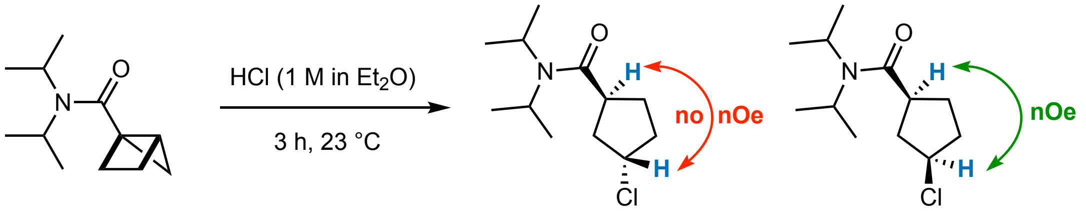

1 **3** (d.r. 2:1) **3'**

A ^{1}H–^{1}H NOESY enhancement between the indicated protons in **3'** (and lack thereof in **3**) confirmed the stereochemistry of these compounds. This assignment of stereochemistry then enabled assignment of the absolute configuration of **(+)-3** as synthesized from **(+)-2** (the absolute stereochemistry of which was in turn assigned by X-ray crystallographic diffraction analysis of a crystalline derivative, see below), and therefore the absolute configuration of housane **(–)-1** (see below).

Ring-opening procedure: 1 (20 mg, 0.10 mmol, 1.0 eq.) was dissolved in CH_2Cl_2 (0.5 mL) and a 1 M solution of HCl in Et_2O (0.12 mL, 0.12 mmol, 1.2 equiv.) was added. The reaction mixture was stirred for 3 h at 23 °C, after which it was concentrated *in vacuo*. The residue was purified by flash column chromatography on silica gel using pentane/diethyl ether (8:2, v/v) to afford **3** and **3'** as colourless liquids. Yield for **3**

(major): 16 mg, 69 µmol, 69% (spectroscopic data identical to that described above). Yield for **3'** (minor): 5 mg, 21 µmol, 16%.

(1*S,3*R**)-3-chloro-*N,N*-diisopropylcyclopentane-1-carboxamide, 3'**

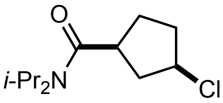

^{1}H NMR (400 MHz, $CDCl_3$) δ 4.25–4.13 (m, 1H), 4.05–3.91 (m, 1H), 3.59– 3.38 (br. s, 1H), 3.00–2.81 (m, 1H), 2.37 (m, 1H), 2.27–2.07 (m, 3H), 2.00 (tt, *J* = 6.8, 3.3 Hz, 1H), 1.81 (ddd, *J* = 11.5, 9.2, 6.6 Hz, 1H), 1.37 (dd, *J* = 6.8, 1.7 Hz, 6H), 1.20 (d, *J* = 6.6 Hz, 6H); **^{13}C NMR** ($CDCl_3$, 101 MHz) δ 172.6, 58.7, 48.1, 45.8, 41.4, 40.5, 36.7, 27.8, 21.2, 20.7; **IR** v_{max} (cm^{-1}): 2960, 1635, 1530, 1466, 1446, 1350, 1299, 1146; **HRMS** (ESI+): calculated for $C_{12}H_{22}{}^{35}ClNONa$ $[M+Na]^+$ 254.1282, found 254.1276.

(1*R,4*R**)-*N,N*-diisopropylbicyclo[2.1.0]pentane-1-carboxamide, 1**

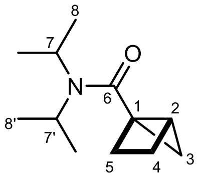

3 (5.00 g, 21.6 mmol, 1.0 equiv.) was dissolved in THF (20 mL) and cooled to 0 °C. LiHMDS (32.3 mL of a 1.0 M solution in THF, 32.3 mmol, 1.5 equiv.) was added dropwise at 0 °C, the cooling bath was removed, and the solution was stirred at ambient temperature (23 °C) overnight. Then, the mixture was diluted with NH_4Cl (aq.) (20 mL) and diethyl ether (20 mL), the organic layer was washed with brine (20 mL), dried with $MgSO_4$ and concentrated *in vacuo*. The crude mixture was purified by flash column chromatography on silica gel using pentane/diethyl ether (7:3 v/v) as eluent to afford **1** (2.60 g, 13.3 mmol, 61%) as a yellow solid.

R_f = 0.3 (pentane/diethyl ether 7:3, v/v); **^{1}H NMR** (400 MHz, $CDCl_3$) $δ_H$ 4.14 (br. s, 1H, H7), 3.29 (br. s, 1H, H7'), 2.30 (tdd, *J* = 10.8, 4.2, 1.7 Hz, 1H, $H5_{exo}$), 2.20–2.01 (m, 2H, H2 and $H4_{exo}$), 1.72–1.62 (m, 1H, $H5_{endo}$), 1.49–1.08 (m, 14H, H8,8' and $H4_{endo}$, $H3_{exo}$), 0.93 (dd, *J* = 4.0, 2.0 Hz, 1H, $H3_{endo}$); **^{13}C NMR** (101 MHz, $CDCl_3$) $δ_C$ 172.1 (C6), 48.6 (C7), 45.7 (C7'), 27.1 (C1), 26.6 (C5), 23.5 (C3), 21.2 (C8/8'), 20.6 (C8/8'), 20.31 (C2), 20.27 (C4); **IR** v_{max} (cm^{-1}): 2968, 1630, 1378, 1040. **HRMS** (ESI+): calculated for $C_{12}H_{21}NONa$ [M+Na]+ 218.1515, found 218.1514.

3. Optimisation of the metalation protocol: Deuterium quench

3.1 Deuterium installation at the bridgehead position of 1 to form d-1:

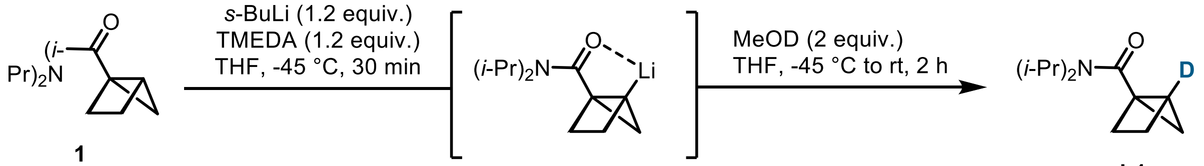

1

d-1
71% (95% D incorporation)

1 (20 mg, 0.10 mmol, 1.0 equiv.) was dissolved in THF (1.0 mL) and TMEDA (18 µL, 0.12 mmol, 1.2 equiv.) was added. The resulting mixture was cooled to -45 °C, then *sec*-BuLi (0.12 mL, 1.05 M in hexane, 0.12 mmol, 1.2 equiv.) was added dropwise and the solution was stirred at -45 °C for 30 min. MeOD (8 µL, 0.20 mmol, 2.0 equiv.) was then added and the solution was stirred at -45 °C for 30 min, then brought to ambient temperature and stirred for a further 1.5 h. $NaHCO_3$ (sat., aq., 0.5 mL) was then added dropwise and the mixture was diluted with diethyl ether (1.0 mL). The aqueous layer was extracted with diethyl ether (3 x 1 mL). The organic layers were combined, washed with brine, dried ($MgSO_4$), then filtered and concentrated *in vacuo*. The crude mixture was purified by flash column chromatography using silica gel with pentane/diethyl ether eluent (8:2, v/v). **d-1** (14 mg, 71 µmol, 71%) was isolated as a colourless liquid. The extent of deuteration was determined by ^{1}H NMR spectroscopy to be 93%.

$\mathbf{R_f}$ = 0.5 (pentane/diethyl ether 8:2, v/v); $\mathbf{^1H}$ **NMR** (400 MHz, $CDCl_3$) δ_H 4.14 (br. s, 1H), 3.30 (br. s, 1H), 2.31 (td, *J* = 10.7, 4.2 Hz, 1H), 2.07 (td, *J* = 10.9, 4.3 Hz, 1H), 1.68 (ddd, *J* = 10.5, 6.1, 4.3 Hz, 1H), 1.51–1.05 (m, 14H), 0.93 (d, *J* = 4.0 Hz, 1H); $\mathbf{^{13}C}$ **NMR** (101 MHz, $CDCl_3$) δ 172.2, 48.6, 45.7, 27.1, 26.6, 23.5, 21.2, 20.9, 20.7, 20.3–20.2 (t, due to isoproyl group it appears broad), 20.2; **IR** ν_{max} (cm^{-1}): 2966, 2925, 2852, 1629, 1444, 1367, 1290, 1040; HRMS (ESI^+): calc. $C_{12}H_{20}DNONa$ $[M+Na]^+$ 219.1578, found 219.1572.

Stacked ^{1}H NMR spectra of **1** (top) and the crude reaction mixture **d-1** (bottom).

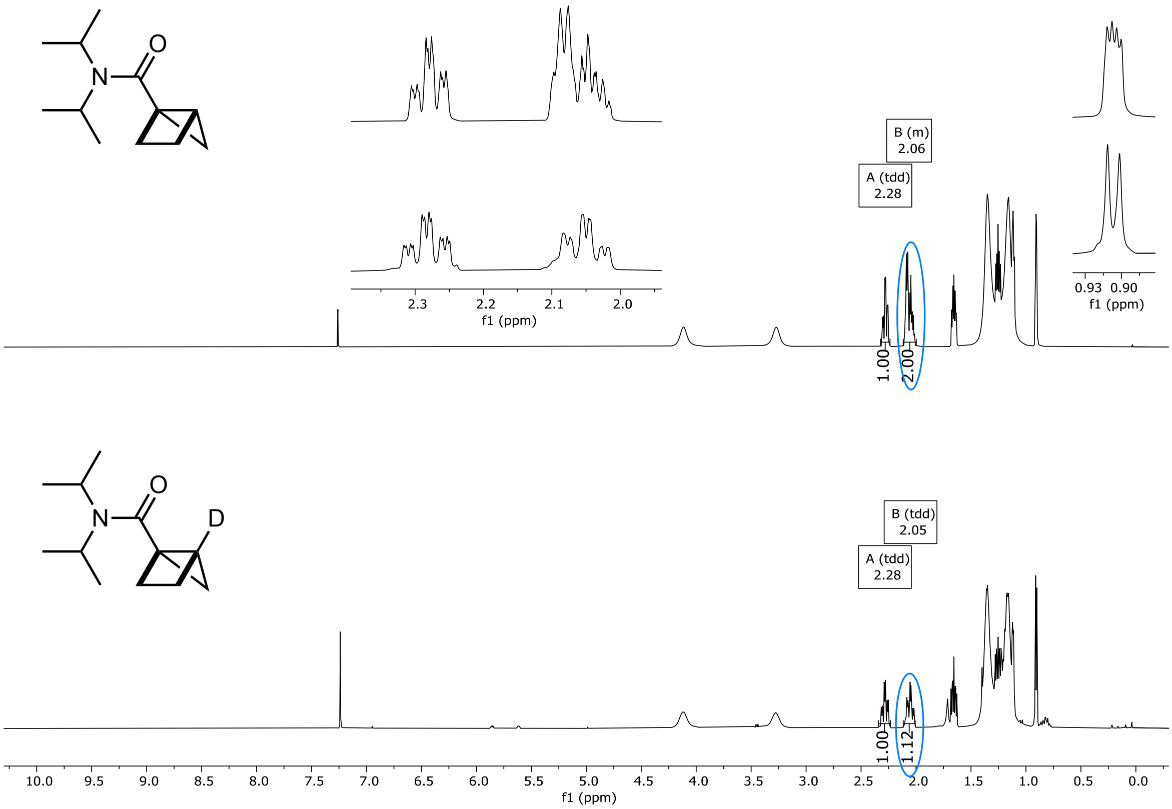

3.2 Deuterium installation at the bridge position to form d-4a

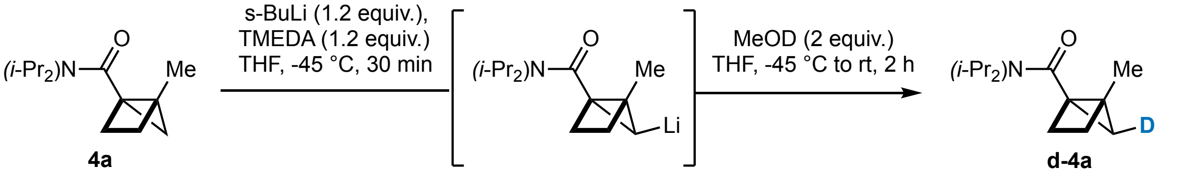

4a **d-4a**

81% (91% D incorporation)

4a (21 mg, 0.10 mmol, 1.0 equiv.) was dissolved in THF (1.5 mL) and TMEDA (28 μL, 0.18 mmol, 1.2 equiv.) was added. The resulting mixture was cooled to -45 °C, then *sec*-BuLi (0.1 mL, 1.4 M in hexane, 0.18 mmol, 1.2 equiv.) was added dropwise and the solution was stirred at -45 °C for 30 min. MeOD (8 μL, 0.20 mmol, 2.0 equiv.) was then added and the solution was stirred at -45 °C for 30 min, then brought to ambient temperature and stirred for a further 1.5 h. $NaHCO_3$ (aq) (1 mL) was then added dropwise and the mixture was diluted with diethyl ether (2 mL). The aqueous layer was extracted with diethyl ether (3 x 2 mL). The organic layers were combined, washed with brine, dried ($MgSO_4$), then filtered and concentrated in vacuo. The residue was purified by flash column chromatography using silica gel with pentane/diethyl ether as

eluent (8:2 v/v) to afford **d-4a** (17 mg, 81 µmol, 81%) as a colourless liquid. The extent of deuteration was determined by ^{1}H NMR spectroscopy to be 91%.

^{1}H NMR (500 MHz, $CDCl_3$) δ_H 4.16 (br. s, 1H), 3.30 (br. s, 1H), 2.28 (td, *J* = 10.6, 3.9 Hz, 1H), 1.87 (td, *J* = 10.6, 4.3 Hz, 1H), 1.60 (ddd, *J* = 10.4, 6.3, 4.2 Hz, 1H), 1.48 (s, 3H), 1.47–1.43 (m, 1H), 1.42–1.00 (m, 12H), 0.96 (s, 1H); **^{13}C NMR** (126 MHz, $CDCl_3$) δ_C 172.7, 48.0, 45.8, 30.6, 30.5, 29.8, 28.4 (t), 26.89, 26.86, 21.3, 21.0, 17.1; **IR** ν_{max} (cm^{-1}): 2966, 1631, 1440, 1369, 1333, 1034; **HRMS** (ESI+): calc. $C_{13}H_{22}DNONa$ $[M+Na]^+$ 233.1735, found 233.1736.

Stacked ^{1}H NMR spectra of **4a** (top) and the crude reaction mixture **d-4a** (bottom).

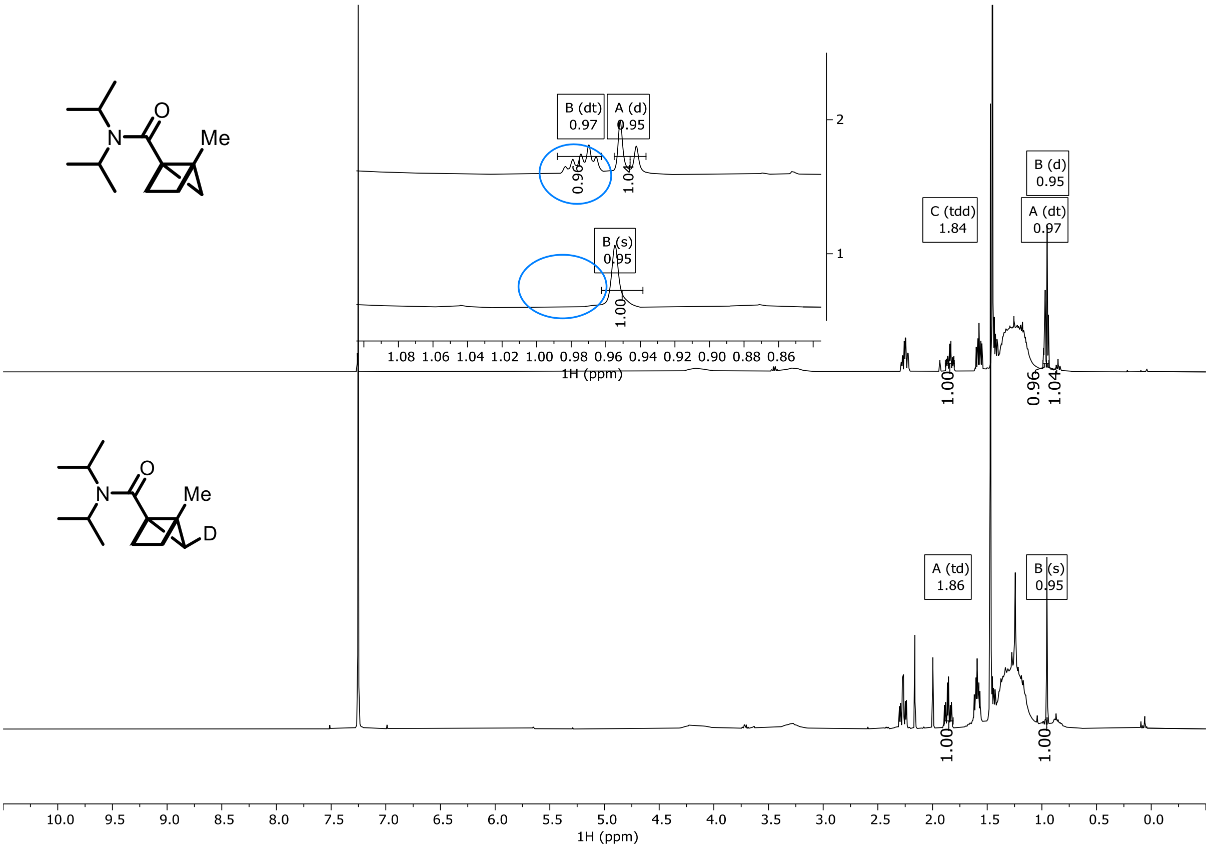

4. General procedures

General procedure A: Compound **1** or **4a** (0.15 mmol, 1.0 equiv.) was dissolved in THF (1.5 mL) and TMEDA (28 µL, 0.18 mmol, 1.2 equiv.) was added. The resulting mixture was cooled to -45 °C, then *sec*-BuLi (0.1 mL, 1.4 M in hexane, 0.18 mmol, 1.2 equiv.) was added dropwise and the solution was stirred at -45 °C for 30 min. The electrophile (2.0 equiv.) was then added slowly and the solution was stirred at -45 °C for 30 min., then brought to ambient temperature and stirred for a further 1.5 h. $NaHCO_3$ (aq., sat., 1 mL) was then added dropwise and the mixture was diluted with diethyl ether (2 mL). The aqueous layer was extracted with diethyl ether (3 x 2 mL). The organic layers were combined, washed with brine, dried ($MgSO_4$), then filtered and concentrated *in vacuo*. The residue was purified by flash column chromatography using silica gel and the indicated eluent. **Note:** if the electrophile is a solid it was first dissolved in 0.5 mL of THF before addition.

General procedure B: Compound **1** or **4a** (0.1 mmol, 1.0 equiv.) was dissolved in THF (0.5 mL) and TMEDA (20 µL, 0.12 mmol, 1.2 equiv.) was added. The resulting mixture was cooled to -45 °C, then *sec*-BuLi (0.1 mL, 1.2 M in *c*-hexane, 0.12 mmol, 1.2 equiv.) was added dropwise and the solution was stirred at -45 °C for 30 min. The electrophile (2.0 equiv.) was then added slowly and the solution was stirred at -45 °C for 30 min, then brought to ambient temperature and stirred for a further 1 h. $NaHCO_3$ (aq., sat., 0.5 mL) was then added dropwise, and the mixture was diluted with diethyl ether (1 mL) and separated. The aqueous layer was extracted with diethyl ether (1 mL), the organic layers were combined, dried ($MgSO_4$), filtered and concentrated *in vacuo*. The residue was purified by flash column chromatography using silica gel and the indicated eluent. **Note**: if the electrophile is a solid it was first dissolved in 0.3 mL of THF before addition.

5. Experimental procedures and characterisation

(1*S**,4*S**)-*N*,*N*-diisopropyl-4-methylbicyclo[2.1.0]pentane-1-carboxamide, 4a

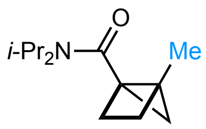

Synthesized according to **General procedure A** using compound **1** (29 mg, 0.15 mmol, 1 equiv.) and iodomethane (19 μL, 0.3 mmol, 2 equiv.). Purification using pentane/diethyl ether (8:2, v/v) as eluent afforded **4a** (24 mg, 0.12 mmol, 76%) as a colourless liquid.

Scale up synthesis: Scale up of General Procedure A using **1** (0.30 g, 1.5 mmol, 1.0 equiv.), THF (13.0 mL), TMEDA (0.3 mL, 1.9 mmol, 1.2 equiv.), *sec*-BuLi (1.3 mL, 1.4 M in THF, 1.9 mmol, 1.2 equiv.), MeI (0.1 mL, 3.0 mmol, 2.0 equiv.) afforded **4a** (0.26 g, 1.2 mmol, 80%) as a colourless liquid.

R_f = 0.4 (pentane/diethyl ether 8:2, v/v); **^{1}H NMR** (500 MHz, $CDCl_3$) δ_H 4.14 (br. s, 1H), 3.25 (br. s, 1H), 2.24 (tdd, *J* = 10.7, 3.9, 1.7 Hz, 1H), 1.83 (tdd, *J* = 10.7, 4.3, 1.7 Hz, 1H), 1.56 (ddd, *J* = 10.6, 6.2, 4.4 Hz, 1H), 1.44 (s, 3H), 1.43–1.40 (m, 1H), 1.40–0.99 (m, 12H), 0.99–0.94 (m, 1H), 0.94 (d, *J* = 3.7 Hz, 1H); **^{13}C NMR** (126 MHz, $CDCl_3$) δ_C 172.5, 48.0, 45.6, 30.52, 30.50, 28.6, 26.85, 26.81, 21.2, 20.9, 17.1; **IR** ν_{max} (cm^{-1}): 2968, 1633, 1439, 1369, 1327, 1044; **HRMS** (ESI+) calculated for $C_{13}H_{24}NO$ $[M+H]^+$ 210.1852, found 210.1846.

(1*S**,4*S**)-4-butyl-*N*,*N*-diisopropylbicyclo[2.1.0]pentane-1-carboxamide, 4b

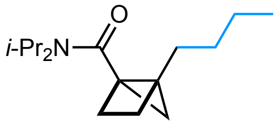

Synthesized according to **General procedure A** using **1** (29 mg, 0.15 mmol, 1.0 equiv.) and 1-iodobutane (34.0 μL, 0.30 mmol, 2.0 equiv.). Purification using pentane/diethyl ether (9:1, v/v) as eluent gave **4b** (31 mg, 0.12 mmol, 82%) as a colourless liquid.

R_f = 0.7 (pentane/diethyl ether 8:2, v/v); **^{1}H NMR** (400 MHz, C_6D_6) δ_H 4.01 (br. s, 1H), 2.95 (br. s, 1H), 2.09 (m, 3H), 1.82 (tdd, J = 10.7, 4.0, 1.9 Hz, 1H), 1.60–1.26 (m, 13H), 1.12 (m, 4H), 0.94 (t, J = 7.1 Hz, 5H), 0.82 (d, J = 3.5 Hz, 2H); **^{13}C NMR** (101 MHz, C_6D_6) δ_C 171.4, 46.9 (br., 2C, due to isopropyl group), 35.0, 31.0, 30.9, 30.1, 28.3, 26.7, 25.2, 23.4, 21.2, 21.0, 14.5; **IR** ν_{max} (cm^{-1}): 2963, 1632, 1439, 1372, 1043; **HRMS** (ESI+) calculated for $C_{16}H_{30}NO$ [M+H]$^+$ 252.2322, found 252.2323.

(1*S**,4*R**)-4-benzyl-*N,N*-diisopropylbicyclo[2.1.0]pentane-1-carboxamide, 4c

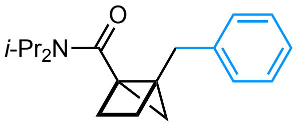

Synthesized according to **General procedure B** using **1** (20 mg, 0.1 mmol, 1.0 equiv.) and benzyl chloride (24.0 µL, 0.20 mmol, 2.0 equiv.). Purification using pentane/diethyl ether (8:2, v/v) as eluent afforded **4c** (15 mg, 51 µmol, 51%) as a clear liquid.

R_f = 0.3 (pentane/diethyl ether 8:2, v/v); **^{1}H NMR** (600 MHz, $CDCl_3$) δ_H 7.29–7.26 (m, 4H), 7.20–7.17 (m, 1H), 4.19 (br, 1H), 3.39–3.27 (br, 1H, H1), 3.35 (d, J = 15.0 Hz, 1H), 3.06 (d, J = 15.0 Hz, H4, 1H), 2.30 (tdd, J = 10.7, 3.9, 1.7 Hz, 1H), 1.92 (tdd, J = 10.8, 4.2, 1.9 Hz, 1H), 1.62 (ddd, J = 10.4, 6.2, 4.2 Hz, 1H), 1.47–1.15 (m, 14H), 1.10 (d, J = 2.9 Hz, 1H); **^{13}C NMR** (151 MHz, $CDCl_3$) δ_C 172.5, 141.0, 129.0, 128.3, 125.8, 48.1, 45.7, 37.2, 34.9, 31.2, 28.5, 26.5, 25.2, 21.3, 20.8; **IR** ν_{max} (cm^{-1}): 2966, 2930, 1629, 1442, 1369, 1331, 1040; **HRMS** (ESI+): calculated for $C_{19}H_{28}NO$ [M+H]$^+$ 286.2165, found 286.2160.

(1*S**,4*R**)-4-allyl-*N,N*-diisopropylbicyclo[2.1.0]pentane-1-carboxamide, 4d

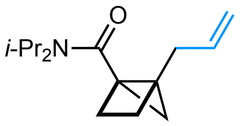

Synthesized according to **General procedure B** using **1** (20 mg, 0.1 mmol, 1.0 equiv.) and allyl bromide (18.0 µL, 0.20 mmol, 2.0 equiv.). Purification using pentane/diethyl ether (9:1, v/v) as eluent afforded **4d** (16 mg, 68 µmol, 66%) as a clear liquid.

R$_f$ = 0.3 (pentane/diethyl ether 9:1, v/v); **^{1}H NMR** (400 MHz, $CDCl_3$) δ_H 5.89 (ddt, *J* = 17.0, 10.2, 6.7 Hz, 1H), 5.06 (d, *J* = 17.2 Hz, 1H), 5.00 (d, *J* = 10.2 Hz, 1H), 4.18 (br, 1H), 3.29 (br. s, 1H), 2.66 (dd, *J* = 15.2, 6.3 Hz, 1H), 2.49 (dd, *J* = 15.1, 7.0 Hz, 1H), 2.27 (tdd, *J* = 10.6, 3.9, 1.8 Hz, 1H), 1.93 (tdd, *J* = 10.7, 4.1, 1.8 Hz, 1H), 1.63–1.58 (m, 2H), 1.44–1.32 (m, 6H), 1.25–1.14 (m, 6H), 1.03 (dt, *J* = 3.7, 1.8 Hz, 1H), 0.98 (d, *J* = 3.8 Hz, 1H); **^{13}C NMR** (126 MHz, $CDCl_3$) δ_C 172.1, 136.8, 115.2, 48.0, 45.5, 35.5, 33.4, 30.6, 27.8, 26.4, 24.5, 21.1, 20.6; **IR** ν_{max} (cm^{-1}): 2964, 2934, 2361, 2331, 1633, 1442, 1370, 1333; **HRMS** (ESI+): calculated for $C_{15}H_{26}NO$ $[M+H]^+$ 236.2009, found 236.2004.

(1*R**,4*R**)-4-bromo-*N,N*-diisopropylbicyclo[2.1.0]pentane-1-carboxamide, 4e

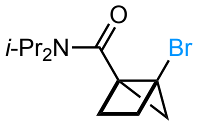

Synthesized according to **General procedure B** using **1** (20 mg, 0.1 mmol, 1.0 equiv.) and NBS (36 mg, 0.20 mmol, 2.0 equiv.). Purification using pentane/diethyl ether (7:3, v/v) as eluent afforded **4e** (17 mg, 62 μmol, 62%) as a white solid.

R$_f$ = 0.3 (pentane/diethyl ether 7:3, v/v); **^{1}H NMR** (500 MHz, $CDCl_3$) δ_H 4.10 (br. s, 1H), 3.33 (br, 1H), 2.53–2.41 (m, 2H), 2.14–2.04 (m, 1H), 1.82–1.74 (m, 1H), 1.62 (dt, *J* = 4.1, 1.9 Hz, 1H), 1.46 (d, *J* = 4.6 Hz, 1H), 1.21 (br. m, 6H), 1.41 (br. m, 6H); **^{13}C NMR** (126 MHz, $CDCl_3$) δ_C 168.2, 48.2, 45.9, 35.1, 34.4, 32.0, 30.0, 27.6, 21.0, 20.8; **IR** ν_{max} (cm^{-1}): 2967, 2360, 1637, 1445, 1369; **HRMS** (ESI+): calculated for $C_{12}H_{21}{}^{79}BrNO$ $[M+H]^+$ 274.0801, found 274.0795.

(1*R**,4*R**)-4-iodo-*N,N*-diisopropylbicyclo[2.1.0]pentane-1-carboxamide, 4f

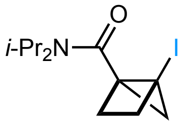

Synthesized according to **General procedure B** using **1** (20 mg, 0.1 mmol, 1.0 equiv.) and NIS (46 mg, 0.20 mmol, 2.0 equiv.). Purification using pentane/diethyl ether (7:3 v/v) as eluent afforded the desired product **4f** (19 mg, 59 μmol, 58%) as a yellow solid.

Scale up synthesis: Scale up of General Procedure B using Compound **1** (140 mg, 0.72 mmol, 1.0 equiv.), THF (3.5 mL), TMEDA (0.15 mL, 0.86 mmol, 1.2 equiv.), *sec*-BuLi (0.75 mL, 1.2 M in *c*-hexane, 0.86 mmol, 1.2 equiv.), NIS (323 mg, 1.43 mmol. 2.0 equiv.) afforded **4f (**150 mg, 0.47 mmol, 65%) as a yellow solid.

R*f* = 0.3 (pentane/diethyl ether 7:3, v/v); **^{1}H NMR** (600 MHz, $CDCl_3$) δ_H 4.11 (br, 1H), 3.34 (br, 1H), 2.54–2.45 (m, 2H), 2.10–2.03 (m, 1H), 2.00–1.90 (m, 1H), 1.56–1.52 (m, 2H), 1.49–1.13 (m, 12H); **^{13}C NMR** (151 MHz, $CDCl_3$) δ_C 168.1, 48.0, 46.1, 33.5, 32.40, 32.35, 28.3, 21.0, 20.8, 2.6; **IR** ν_{max} (cm^{-1}): 3351, 2968, 2937, 1624, 1512, 1445, 1369, 1235, 1038; **HRMS** (ESI+): calculated for $C_{12}H_{20}INONa$ $[M+Na]^+$ 344.0482, found 344.0472.

(1*R**,4*R**)-*N,N*-diisopropyl-4-(trimethylsilyl)bicyclo[2.1.0]pentane-1-carboxamide, 4g

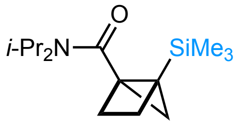

Synthesized according to **General procedure B** using compound **1** (20 mg, 0.1 mmol, 1.0 equiv.) and TMSCl (26.0 µL, 0.20 mmol, 2.0 equiv.). Purification using pentane/diethyl ether (9:1, v/v) as eluent afforded **4g** (19 mg, 71 µmol, 69%) as a viscous liquid.

R*f* = 0.8 (pentane/diethyl ether 9:1, v/v); **^{1}H NMR** (500 MHz, $CDCl_3$) δ_H 4.26 (br. s, 1H), 3.28 (br. s, H1), 2.30 (tdd, *J* = 10.6, 4.6, 1.6 Hz, 1H), 1.89 (tdd, *J* = 10.6, 4.1, 1.4 Hz, 1H), 1.80 (ddd, *J* = 10.6, 6.4, 4.1 Hz, 1H), 1.45–1.13 (m, 13H), 1.12–1.10 (m, 1H), 1.01 (d, *J* = 3.6 Hz, 1H), 0.05 (s, 9H); **^{13}C NMR** (126 MHz, $CDCl_3$) δ_C 171.4, 48.5, 45.4, 32.4, 27.0, 26.6, 22.1, 21.8, 21.3, 21.1, 20.6, -2.0; **IR** ν_{max} (cm^{-1}): 2964, 2363, 2332, 1636, 1444, 1360; **HRMS** (ESI+): calculated for $C_{15}H_{29}NOSiNa$ $[M+Na]^+$ 290.1911, found 290.1922.

(1*S,4*S**)-*N*,*N*-diisopropyl-4-(4,4,5,5-tetramethyl-1,3,2-dioxaborolan-2-yl)bicyclo[2.1.0]pentane-1-carboxamide**, **4h**

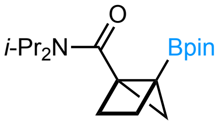

Synthesized according to **General procedure A** using **1** (29 mg, 0.15 mmol, 1.0 equiv.) and 2-isopropoxy-4,4,5,5-tetramethyl-1,3,2-dioxaborolane (61.0 µL, 0.30 mmol, 2.0 equiv.). Purification using pentane/diethyl ether (1:1, v/v) as eluent afforded **4h** (36 mg, 0.11 mmol, 75%) as a colourless liquid.

R*f* = 0.4 (pentane/diethyl ether 45:55, v/v); **^{1}H NMR** (400 MHz, C_6D_6) δ_H 3.97 (br, 1H), 2.95 (br, 1H), 2.41 (tdd, *J* = 10.8, 4.3, 1.3 Hz, 1H), 2.15 (tdd, *J* = 10.8, 4.4, 1.6 Hz, 1H), 1.74 (d, *J* = 3.7 Hz, 1H), 1.61–1.33 (m, 7H), 1.29 (s, 6H), 1.26 (s, 6H), 1.18 (ddd, J = 10.8, 6.5, 4.4 Hz, 1H), 0.96 (d, *J* = 3.6 Hz, 1H), 0.82 (s, 6H); **^{13}C NMR** (101 MHz, C_6D_6) δ_C 170.1, 83.4, 48.5, 45.5, 36.0, 27.1, 26.1, 25.5, 25.1, 25.0, 21.0, 20.8; **^{11}B NMR** (128 MHz, C_6D_6) δ 32.13; **IR** ν_{max} (cm^{-1}): 2974, 1638, 1427, 1351, 1313, 1137, 1042; **HRMS** (ESI+) calculated for $C_{18}H_{33}BNO_3$ $[M+H]^+$ 322.2548, found 322.2548.

(1*S,4*R**)-4-benzoyl-*N*,*N*-diisopropylbicyclo[2.1.0]pentane-1-carboxamide**, **4i**

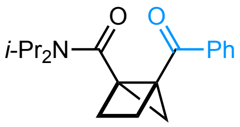

Synthesized according to **General procedure A** (with reaction quenching and workup using water) using **1** (29 mg, 0.15 mmol, 1.0 equiv.) and *N*-methoxy-*N*-methylbenzamide (45.0 µL, 0.30 mmol, 2.0 equiv.). Purification using pentane/diethyl ether (1:1, v/v) as eluent afforded **4i** (32 mg, 0.11 mmol, 72%) as a colourless solid.

R*f* = 0.3 (pentane:diethyl ether 1:1, v/v); **^{1}H NMR** (500 MHz, C_6D_6) δ_H 7.93–7.80 (m, 2H), 7.13–7.06 (m, 3H), 3.91 (br, 1H), 2.93 (br, 1H), 2.54 (tdd, *J* = 11.0, 4.2, 1.7 Hz, 1H), 2.35 (d, *J* = 4.1 Hz, 1H), 2.17 (tdd, *J* = 10.9, 4.2, 1.8 Hz, 1H), 1.59–1.23 (m, 8H), 1.17 (d, *J* = 4.1 Hz, 1H), 0.96–0.63 (m, 6H); **^{13}C NMR** (126 MHz, C_6D_6) δ_C 198.2, 167.4, 139.3, 131.4, 128.8, 48.8, 45.9, 43.0, 38.9, 29.1, 25.0, 23.7, 21.0, 20.8; **IR** ν_{max} (cm^{-1}): 2970, 1663, 1631, 1445, 1374, 1330, 1042; **HRMS** (ESI+) calculated for $C_{19}H_{26}NO_2$ $[M+H]^+$ 300.1958, found 300.1956.

(1*S,4*R**)-4-((4-bromophenyl)(hydroxy)methyl)-*N*,*N*-diisopropylbicyclo[2.1.0] pentane-1-carboxamide, 4j**

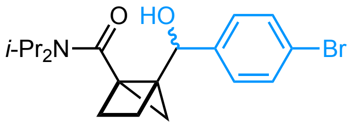

Synthesized according to **General procedure B** using **1** (20 mg, 0.1 mmol, 1.0 equiv.) and 4-bromobenzaldehyde (38 mg, 0.20 mmol, 2.0 equiv.). The title compound **4j** showed a 3:1 diastereomeric ratio in the ^{1}H NMR spectrum of the crude mixture and was purified by flash column chromatography using pentane/diethyl ether (9:1, v/v) as eluent, with the two diastereomers isolated as white solids (major isomer: 15 mg, 39 μmol, 38%; minor compound: 10 mg, 26 μmol, 26%). The stereochemistry of the major diastereomer was determined by single crystal X-ray diffraction analysis.

$\mathbf{R}_f$ = 0.4 (major) and 0.3 (minor) (pentane:diethyl ether 7:3, v/v)

Major Compound: **(1*S**,4*R**)-4-((*S**)-(4-bromophenyl)(hydroxy)methyl)-*N*,*N*-diisopropylbicyclo[2.1.0]pentane-1-carboxamide, 4j**

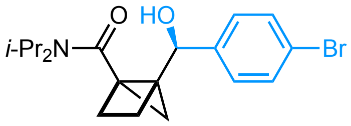

$\mathbf{R}_f$ = 0.4 (pentane:diethyl ether 7:3, v/v); ^{1}**H NMR** (500 MHz, $CDCl_3$) δ_H 7.45 (d, *J* = 8.4 Hz, 2H), 7.33 (d, *J* = 7.9 Hz, 2H), 5.51 (d, *J* = 2.7 Hz, 1H), 4.72 (d, *J* = 2.3 Hz, 1H), 4.23 (hept, *J* = 6.7 Hz, 1H), 3.36 (hept, *J* = 6.8 Hz, 1H), 2.24 (tdd, *J* = 10.5, 3.7, 1.8 Hz, 1H), 2.14 (tdd, *J* = 10.8, 4.1, 1.7 Hz, 1H), 1.57 (ddd, *J* = 10.3, 6.2, 4.0 Hz, 1H), 1.50 (dt, *J* = 3.8, 1.7 Hz, 1H), 1.42 (d, *J* = 6.8 Hz, 6H), 1.22 (d, *J* = 6.5 Hz, 3H), 1.19 (d, *J* = 6.7 Hz, 3H), 1.07 (ddd, *J* = 10.3, 6.1, 3.7 Hz, 1H); ^{13}**C NMR** (126 MHz, $CDCl_3$) δ_C 172.4, 140.7, 131.3, 128.0, 120.6, 73.3, 49.4, 46.0, 38.0, 31.3, 30.4, 27.8, 26.0, 21.00, 20.98, 20.2, 19.7; **IR** ν_{max} (cm^{-1}): 2999, 1630, 1476, 1333; **HRMS** (ESI+): calculated for $C_{19}H_{27}{}^{79}BrNO_2$ $[M+H]^+$ 380.1220, found 380.1213.

Minor compound: (1*S,4*R**)-4-((*S**)-(4-bromophenyl)(hydroxy)methyl)-*N*,*N*-diisopropylbicyclo[2.1.0]pentane-1-carboxamide, 4j'**

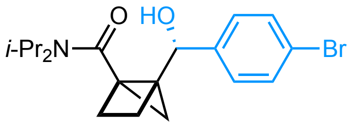

R*f* = 0.3 (pentane:diethyl ether 7:3, v/v); **^{1}H NMR** (500 MHz, $CDCl_3$) δ_H 7.44 (d, *J* = 8.4 Hz, 2H), 7.28 (d, *J* = 8.4 Hz, 2H), 6.08 (s, 1H), 5.15 (s, 1H), 4.11 (hept, *J* = 6.8 Hz, 1H), 3.35 (hept, *J* = 6.7 Hz, 1H), 2.35 (tdd, *J* = 10.7, 3.9, 1.7 Hz, 1H), 1.99 (tdd, *J* = 10.8, 4.2, 1.8 Hz, 1H), 1.77 (dt, *J* = 3.8, 1.8 Hz, 1H), 1.62 (ddd, *J* = 10.6, 6.3, 4.2 Hz, 1H), 1.45–1.37 (m, 6H), 1.29–1.23 (m, 1H), 1.21–1.18 (m, 6H), 0.91 (d, *J*= 4.0 Hz, 1H); **^{13}C NMR** (126 MHz, $CDCl_3$) δ_C 173.3, 141.4, 131.4, 128.3, 121.1, 71.3, 49.4, 46.2, 39.7, 33.8, 26.1, 23.3, 22.2, 21.0, 20.97, 20.93, 20.4; **IR** ν_{max} (cm^{-1}): 3249, 2971, 2935, 1599, 1477, 1458, 1369, 1330, 1070, 1040; **HRMS** (ESI+): calculated for $C_{19}H_{27}{}^{79}BrNO_2$ $[M+H]^+$ 380.1220, found 380.1213 and 380.1212.

(1*S,4*R**)-4-(2-hydroxypropan-2-yl)-*N,N*-diisopropylbicyclo[2.1.0]pentane-1-carboxamide, 4k**

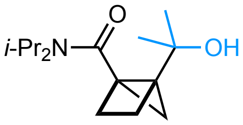

Synthesized according to **General procedure B** using **1** (20 mg, 0.1 mmol, 1.0 equiv.) and acetone (16.0 µL, 0.20 mmol, 2.0 equiv.). Purification using pentane/diethyl ether (7:3, v/v) as eluent afforded **4k** (15 mg, 59 µmol, 58%) as a white solid.

R*f* = 0.3 (pentane/diethyl ether 7:3, v/v); **^{1}H NMR** (500 MHz, $CDCl_3$) δ_H 5.17 (s, 1H), 4.21 (hept, *J* = 6.7 Hz, 1H), 3.34 (hept, *J* = 6.8 Hz,1H), 2.30 (tdd, *J* = 10.6, 4.1, 1.8 Hz, 1H), 2.10 (tdd, *J* = 10.8, 3.9, 2.0 Hz, 1H), 1.68–1.62 (m, 3H), 1.42 (s, 3H), 1.40 (d, *J* = 6.7 Hz, 3H), 1.38 (d, *J* = 6.7 Hz, 3H), 1.22 (d, *J* = 6.7 Hz, 3H), 1.20 (d, *J* = 6.7 Hz, 3H), 1.19 (s, 3H), 0.94 (d, *J* = 3.8 Hz, 1H); **^{13}C NMR** (126 MHz, $CDCl_3$) δ_C 173.2, 67.8, 49.1, 46.0, 43.4, 32.8, 28.0, 26.7, 24.3, 22.6, 21.08, 21.06, 20.9, 20.3; **IR** ν_{max} (cm^{-1}): 3258, 2969, 2933, 1586, 1458, 1333, 1042. **HRMS** (ESI+): calculated for $C_{15}H_{28}NO_2$ $[M+H]^+$ 254.2115, found 254.2110.

(1*S,4*R**)- 4-(1-hydroxycyclobutyl)-*N*,*N*-diisopropylbicyclo[2.1.0]pentane-1-carboxamide**, **4l**

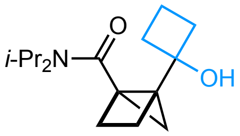

Synthesized according to **General procedure A** using **1** (29 mg, 0.15 mmol, 1.0 equiv.) and cyclobutanone (22.0 μL, 0.30 mmol, 2.0 equiv.). Purification using pentane/diethyl ether (65:35, v/v) as eluent afforded **4l** (19 mg, 72 μmol, 48%) as a colourless solid.

R*f* = 0.5 (pentane/diethyl ether 7:3, v/v); **^{1}H NMR** (500 MHz, C_6D_6) δ_H 5.68 (s, 1H), 3.88 (hept, *J* = 6.7 Hz, 1H), 2.87 (hept, *J* = 6.8 Hz, 1H), 2.52–2.43 (m, 1H), 2.43–2.34 (m, 2H), 2.26–2.18 (m, 1H), 2.16 –2.09 (m, 1H), 2.05–1.98 (m, 1H), 1.98–1.90 (m, 1H), 1.66–1.63 (m, 1H), 1.62–1.52 (m, 1H), 1.35 (d, *J* = 6.8 Hz, 6H), 1.29–1.23 (m, 2H), 0.82–0.75 (m, 7H); **^{13}C NMR** (126 MHz, $CDCl_3$) δ_C 172.8, 73.4, 48.9, 45.9, 40.2, 34.1, 32.1, 26.3, 25.2, 21.9, 20.9, 20.7, 20.6, 20.2, 13.9; **IR** ν_{max} (cm^{-1}): 3319 (br.), 2965, 1597, 1463, 1342, 1368, 1156, 1082; **HRMS** (ESI+) calculated for $C_{16}H_{28}NO_2$ $[M+H]^+$ 266.2115, found 266.2117.

(1*S,4*R**)- 4-(1-hydroxycyclohexyl)-*N*,*N*-diisopropylbicyclo[2.1.0]pentane-1-carboxamide**, **4m**

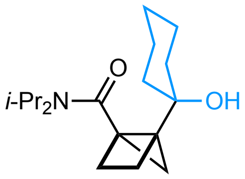

Synthesized according to **General procedure A** using **1** (29 mg, 0.15 mmol, 1.0 equiv.) and cyclohexanone (31.0 μL, 0.30 mmol, 2.0 equiv.). Purification using pentane/diethyl ether (8:2, v/v) as eluent afforded **4m** (23 mg, 78 μmol, 52%) as a white solid.

R*f* = 0.5 (pentane/diethyl ether 8:2, v/v); **^{1}H NMR** (500 MHz, C_6D_6) δ_H 5.16 (s, 1H), 3.87 (hept, *J* = 6.8 Hz, 1H), 2.86 (hept, *J* = 6.7 Hz, 1H), 2.28–2.13 (m, 2H), 2.11–1.95

(m, 3H), 1.88–1.80 (m, 2H), 1.80–1.73 (m, 1H), 1.72–1.56 (m, 3H), 1.40–1.32 (m, 7H), 1.28–1.15 (m, 3H), 0.79 (d, *J* = 6.7 Hz, 3H), 0.77 (d, *J* = 6.7 Hz, 3H), 0.69 (d, *J* = 3.6 Hz, 1H); **^{13}C NMR** (126 MHz, C_6D_6) δ_C 173.3, 68.2, 48.7, 46.0, 44.2, 35.8, 35.1, 32.6, 26.8, 24.3, 22.4, 22.3, 22.1, 21.0, 20.68, 20.65, 20.3; **IR** ν_{max} (cm^{-1}): 3276 (br.), 2969, 1596, 1457, 1365, 1329, 1043; **HRMS** (ESI+) calculated for $C_{18}H_{31}NO_2Na$ $[M+Na]^+$ 316.2247, found 316.2244.

(1*S,4*R**)-4-(hydroxydiphenylmethyl)-*N,N*-diisopropylbicyclo[2.1.0]pentane-1-carboxamide, 4n**

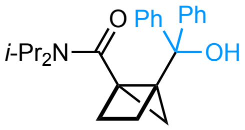

Synthesized according to **General procedure B** using **1** (20 mg, 0.1 mmol, 1.0 equiv.) and benzophenone (38 mg, 0.20 mmol, 2.0 equiv.). Purification using pentane/diethyl ether (9:1, v/v) as eluent afforded **4n** (14 mg, 37 µmol, 36%) as a white solid.

R_f = 0.3 (pentane:diethyl ether 9:1, v/v); **^{1}H NMR** (500 MHz, $CDCl_3$) δ_H 7.45 (dd, *J* = 8.3, 1.3 Hz, 2H), 7.41 (dd, *J* = 8.4, 1.4 Hz, 2H), 7.33–7.27 (m, 4H), 7.26–7.19 (m, 2H), 4.18 (hept, *J* = 6.7 Hz, 1H), 3.35 (hept, *J* = 6.8 Hz, 1H), 2.38 (tdd, *J* = 10.7, 4.2, 1.8 Hz, 1H), 2.10 (tdd, *J* = 10.8, 3.8, 2.0 Hz, 1H), 1.82 (dt, *J* = 3.9, 1.9 Hz, 1H), 1.68 (ddd, *J* = 10.4, 6.4, 3.8 Hz, 1H), 1.46 (ddd, *J* = 10.5, 6.2, 4.0 Hz, 1H), 1.41 (dd, *J* = 6.8 Hz, 3H), 1.39 (d, *J* = 6.8 Hz, 3H) 1.22 (d, *J* = 6.6 Hz, 3H), 1.18 (d, *J* = 6.7 Hz, 3H), 1.06 (d, *J* = 3.9 Hz, 1H); **^{13}C NMR** (126 MHz, $CDCl_3$) δ_C 173.1, 147.2, 146.1, 128.1, 127.9, 127.7, 126.9, 126.7, 126.5, 49.2, 46.3, 43.8, 36.2, 26.0, 25.2, 25.1, 21.1, 21.1, 20.9, 20.3; **IR** ν_{max} (cm^{-1}): 3299 (br.), 2362, 1599, 1476, 1381, 1041. **HRMS** (ESI+): calculated for $C_{25}H_{32}NO_2$ $[M+H]^+$ 378.2428, found 378.2420.

(1S^*,4S^*,5S^*)-N,N-diisopropyl-4,5-dimethylbicyclo[2.1.0]pentane-1-carboxamide, 5a

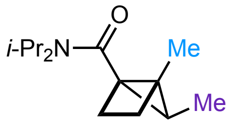

Synthesized according to **General procedure B** using **4a** (21 mg, 0.10 mmol, 1.0 equiv.) and iodomethane (13.0 µL, 0.20 mmol, 2.0 equiv.). Purification using pentane/diethyl ether (8:2, v/v) as eluent afforded **5a** (16 mg, 71 µmol, 71%) as a white solid.

R_f = 0.4 (pentane/diethyl ether 8:2, v/v); **^{1}H NMR** (600 MHz, $CDCl_3$) δ_H 4.19 (hept, J = 6.7 Hz, 1H), 3.27 (hept, J = 6.7 Hz, 1H), 2.25 (td, J = 10.7, 4.0 Hz, 1H), 1.90 (td, J = 10.8, 4.3 Hz, 1H), 1.55 (ddd, J = 10.5, 6.2, 4.3 Hz, 1H), 1.45–1.42 (m, 1H), 1.41 (d, J = 6.9 Hz, 3H), 1.38 (d, J = 6.8 Hz, 3H), 1.36 (s, 3H), 1.19 (d, J = 6.7 Hz, 3H), 1.16–1.14 (m, 1H), 1.12 (d, J = 6.8 Hz, 3H), 1.00 (d, J = 6.3 Hz, 1H); **^{13}C NMR** (151 MHz, $CDCl_3$) δ_C 171.4, 48.3, 45.4, 34.5, 32.1, 31.6, 27.6, 26.8, 21.4, 21.3, 21.2, 20.6, 12.3, 9.6; **IR** ν_{max} (cm^{-1}): 2970, 2934, 1796, 1718, 1632, 1504, 1348, 1160; **HRMS** (ESI+): calculated for $C_{14}H_{26}NO$ $[M+H]^+$ 224.2009, found 224.2013.

(1S^*,4S^*,5S^*)-N,N-diisopropyl-4-methyl-5-propylbicyclo[2.1.0]pentane-1-carboxamide, 5b

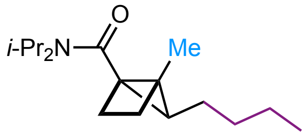

Synthesized according to **General procedure A** using **4a** (31 mg, 0.15 mmol, 1.0 equiv.) and 1-iodopropane (29.0 µL, 0.30 mmol, 2.0 equiv.). Purification using pentane/diethyl ether (8:2, v/v) as eluent afforded **5b** (22 mg, 86 µmol, 57%) as a colourless liquid.

R_f = 0.5 (pentane/diethyl ether 8:2, v/v); **^{1}H NMR** (500 MHz, C_6D_6) δ_H 4.00–3.80 (m, 1H), 3.05–2.84 (m, 1H), 2.15–2.06 (m, 1H), 1.78–1.69 (m, 1H), 1.62 (s, 3H), 1.59–1.30 (m, 12H), 1.02 (t, J = 6.9 Hz, 1H), 0.98–0.81 (m, 9H); **^{13}C NMR** (126 MHz, C_6D_6) δ_C 170.7, 47.7, 45.5, 38.3, 33.6, 32.7, 28.0, 27.4, 27.2, 23.1, 21.4, 21.3, 21.1, 20.7, 14.4,

13.2; **IR** ν_{max} (cm^{-1}): 2962, 1631, 1440, 1372, 1321, 1271, 1215, 1040; **HRMS** (ESI+) calculated for $C_{16}H_{29}NONa$ $[M+Na]^+$ 274.2141, found 274.2138.

(1*S,4*S**,5*S**)-*N,N*-diisopropyl-5-(methoxymethyl)-4-methylbicyclo[2.1.0] pentane -1-carboxamide**, **5c**

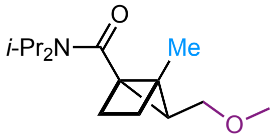

Synthesized according to **General procedure A** using **4a** (31 mg, 0.15 mmol, 1.0 equiv.) and chloromethyl methyl ether (23.0 μL, 0.30 mmol, 2.0 equiv.). Purification using pentane/diethyl ether (75:25, v/v) as eluent afforded **5c** (28 mg, 0.11 mmol, 73%) as a colourless liquid.

$\mathbf{R_f}$ = 0.3 (pentane:diethyl ether 8:2, v/v); **^{1}H NMR** (500 MHz, C_6D_6) δ_H 4.07–3.93 (m, 1H), 3.51 (dd, *J* = 9.9, 5.4 Hz, 1H), 3.40 (pt, *J* = 9.5, 9.5 Hz, 1H), 3.11 (s, 3H), 3.04–2.94 (m, 1H), 2.08 (td, *J* = 10.6, 3.8 Hz, 1H), 1.67 (td, *J* = 10.6, 4.0 Hz, 1H), 1.59 (s, 3H), 1.53 (d, *J* = 6.7 Hz, 6H), 1.42 (dd, *J* = 9.2, 5.3 Hz, 1H), 1.36 (ddd, *J* = 10.1, 6.2, 4.0 Hz, 1H), 1.30 (ddd, *J* = 10.2, 6.2, 3.7 Hz, 1H), 1.02 (d, *J* = 6.7 Hz, 3H), 0.87 (d, *J* = 6.7 Hz, 3H); **^{13}C NMR** (126 MHz, C_6D_6) δ_C 170.3, 69.6, 58.4, 48.1, 45.5, 37.5, 33.8, 31.8, 28.1, 27.2, 21.3, 21.2, 20.7, 20.6, 13.3; **IR** ν_{max} (cm^{-1}): 2965, 1630, 1442, 1375, 1344, 1214, 1109; **HRMS** (ESI+) calculated for $C_{15}H_{27}NO_2Na$ $[M+Na]^+$ 276.1934, found 276.1934.

(1*S,4*R**,5*S**)-5-bromo-*N,N*-diisopropyl-4-methylbicyclo[2.1.0]pentane-1-carboxamide**, **5d**

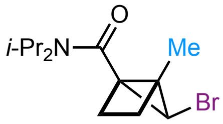

Synthesized according to**General procedure A** using **4a** (31 mg, 0.15 mmol, 1.0 equiv.) and *N*-bromosuccinimide (53 mg, 0.30 mmol, 2.0 equiv.). Purification using pentane/diethyl ether (8:2, v/v) as eluent afforded **5d** (33 mg, 0.11 mmol, 76%) as a colourless solid.

R$_f$ = 0.3 (pentane/diethyl ether 8:2, v/v); **^{1}H NMR** (500 MHz, C_6D_6) δ_H 3.80 (hept, *J* = 6.7 Hz, 1H), 2.97 (hept, *J* = 6.8 Hz, 1H), 2.74 (s, 1H), 2.05–1.95 (m, 1H), 1.76–1.69 (m, 1H), 1.67 (s, 3H), 1.55–1.49 (m, 6H), 1.13–1.05 (m, 2H), 1.05 (d, *J* = 6.6 Hz, 3H), 0.79 (d, *J* = 6.7 Hz, 3H); **^{13}C NMR** (126 MHz, C_6D_6) δ_C 167.3, 48.5, 45.8, 38.2, 35.9, 32.6, 26.6, 26.1, 21.2, 21.0, 20.9, 20.6, 14.2; **IR** ν_{max} (cm^{-1}): 2969, 1637, 1445, 1371, 1332, 1243, 1209, 1038; **HRMS** (ESI+) calculated for $C_{13}H_{22}BrNONa$ $[M+Na]^+$ 310.0777, found 310.0781.

(1*S,4*R**,5*S**)-*N,N*-diisopropyl-4-methyl-5-(trimethylsilyl)bicyclo[2.1.0]pentane-1-carboxamide, 5e**

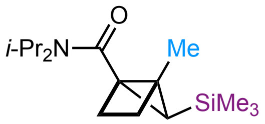

Synthesized according to **General procedure B** using **4a** (21 mg, 0.10 mmol, 1.0 equiv.) and TMSCl (26.0 µL, 0.20 mmol, 2.0 equiv.). Purification using pentane/diethyl ether (8:2, v/v) as eluent afforded **5e (**17 mg, 60 µmol, 60%) as a white solid.

R$_f$ = 0.4 (pentane/diethyl ether 8:2, v/v); **^{1}H NMR** (600 MHz, $CDCl_3$) δ_H 4.24 (br. s, 1H), 3.29 (br, 1H), 2.27 (td, *J* = 10.4, 3.7 Hz, 1H), 1.90 (td, *J* = 10.4, 4.2 Hz, 1H), 1.57 (ddd, *J* = 10.4, 6.2, 4.1 Hz, 1H), 1.50 (s, 3H), 1.48 (td, *J* = 6.4, 3.1 Hz, 1H), 1.40 (br, 6H), 1.22 (d, *J* = 6.6 Hz, 3H), 1.14 (d, *J* = 6.2 Hz, 3H), 0.15 (s, 1H), 0.08 (s, 9H); **^{13}C NMR** (151 MHz, $CDCl_3$) δ_C 172.2, 48.2, 45.5, 34.4, 32.2, 30.0, 29.9, 29.7, 21.6, 21.3, 21.0, 20.6, 16.5, 0.2; **IR** ν_{max} (cm^{-1}): 2963, 1795, 1718, 1633, 1439; **HRMS** (ESI+): calculated for $C_{16}H_{32}NOSi$ $[M+H]^+$: 282.2248, found 282.2242.

One-pot electrophilic functionalization : Compound **1** (20 mg, 0.10 mmol, 1.0 equiv.) was dissolved in THF (1.0 mL) and TMEDA (18 µL, 0.12 mmol, 1.2 equiv.) was added. The resulting mixture was cooled to -45 °C, *sec*-BuLi (0.12 mL, 1.05 M in hexane, 0.12 mmol, 1.2 equiv.) was added dropwise and the solution was stirred at -45 °C for 30 min. MeI (8 µL, 0.12 mmol, 1.2 equiv.) was then added slowly and the solution was stirred at -45 °C for 30 min, then brought to ambient temperature (23 °C) and stirred for a further 30 min.Then, the mixture was again cooled to -45 °C, TMEDA (18 µL, 0.12 mmol, 1.2 equiv.) was added, followed by the dropwise addition of *sec*-

BuLi (0.12 mL, 1.05 M in hexane, 0.12 mmol, 1.2 equiv.) after which the solution was stirred at -45 °C for 30 min. TMSCl (26 μL, 0.20 mmol, 2.0 equiv.) was then added slowly and the solution was stirred at -45 °C for 30 min, then brought to ambient temperature (23 °C) and stirred for a further 30 min. $NaHCO_3$ (aq) (0.5 mL) was then added dropwise and the mixture was diluted with diethyl ether (1.0 mL). The aqueous layer was extracted with diethyl ether (1.0 mL × 3). The organic layers were combined, washed with brine, dried ($MgSO_4$), then filtered and concentrated *in vacuo*. The crude mixture was purified using pentane/diethyl ether (8:2, v/v) as eluent afforded **5e** (11 mg, 39 μmol, 38%) as whilte solid.

(1*S,4*S**,5*S**)-5-benzoyl-*N*,*N*-diisopropyl-4-methylbicyclo[2.1.0]pentane-1-carboxamide**, **5f**

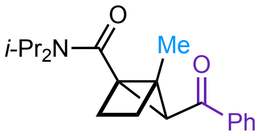

Synthesized according to **General procedure A** using **4a** (31 mg, 0.15 mmol, 1.0 equiv.) and *N*-methoxy-*N*-methylbenzamide (46.0 μL, 0.30 mmol, 2.0 equiv.). Purification using pentane/diethyl ether (6:4, v/v) as eluent afforded **5f** (42 mg, 0.13 mmol, 89%) as a colourless solid.

R_f = 0.2 (pentane/diethyl ether 6:4, v/v); **^{1}H NMR** (500 MHz, C_6D_6) δ_H 7.94–7.89 (m, 2H), 7.15–7.13 (m, 1H), 7.12–7.07 (m, 2H), 4.00 (hept, *J* = 6.7 Hz, 1H), 3.03 (hept, *J* = 6.5 Hz, 1H), 2.60 (s, 1H), 2.19 (td, *J* = 10.4, 3.7 Hz, 1H), 1.89 (td, *J* = 10.7, 3.9 Hz, 1H), 1.73 (d, *J* = 6.7 Hz, 3H), 1.61 (d, *J* = 6.7 Hz, 3H), 1.54 (s, 3H), 1.47 (m, 1H), 1.40 (m, 1H), 0.89 (d, *J* = 6.7 Hz, 3H), 0.84 (d, *J* = 6.7 Hz, 3H); **^{13}C NMR** (126 MHz, C_6D_6) δ_C 195.8, 167.2, 139.4, 132.5, 128.7, 128.4, 48.5, 45.6, 44.8, 41.6, 40.0, 29.1, 26.4, 21.5, 21.0, 20.8, 20.3, 12.7; **IR** ν_{max} (cm^{-1}): 2968, 1671, 1637, 1448, 1372, 1323, 1224, 1018; **HRMS** (ESI+) calculated for $C_{20}H_{27}NO_2Na$ [M+Na]$^+$ 336.1934, found 336.1936.

(1*S,4*S**,5*S**)-5-(1-hydroxycyclohexyl)-*N*,*N*-diisopropyl-4-methylbicyclo[2.1.0] pentane-1-carboxamide**, **5g**

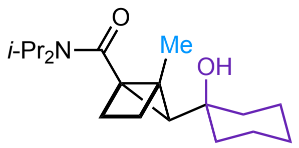

Synthesized according to **General procedure A** (with reaction quenching and workup using water) using **4a** (31 mg, 0.15 mmol, 1.0 equiv.) and cyclohexanone (31 µL, 0.30 mmol, 2.0 equiv.). Purification using pentane/diethyl ether (8:2, v/v) as eluent afforded **5g** (32 mg, 0.10 mmol, 69%) as a colourless solid.

R_f = 0.5 (pentane diethyl ether 8:2, v/v); **^{1}H NMR** (600 MHz, $CDCl_3$) δ_H 4.27 (s, 1H), 4.12 (br, 1H), 3.33 (br, 1H), 2.26 (td, *J* = 10.8, 3.9 Hz, 1H), 1.84–1.76 (m, 2H), 1.72 (s, 3H), 1.72–1.63 (m, 4H), 1.57–1.13 (m, 20H); **^{13}C NMR** (151 MHz, $CDCl_3$) δ_C 175.0, 70.2, 48.8, 48.5, 46.3, 40.6, 39.2, 35.8, 32.9, 29.6, 28.2, 26.0, 22.1, 21.5, 21.1, 20.8, 14.7; **IR** ν_{max} (cm^{-1}): 3339 (br.), 2933, 1593, 1450, 1370, 1322, 1078, 1041; **HRMS** (ESI+) calculated for $C_{19}H_{34}NO_2$ $[M+H]^+$ 308.2584, found 308.2584.

(1*S,4*S**,5*S**)-*N*,*N*-diisopropyl-4-methyl-5-(4,4,5,5-tetramethyl-1,3,2-dioxaborolan-2-yl)bicyclo[2.1.0]pentane-1-carboxamide**, **5h**

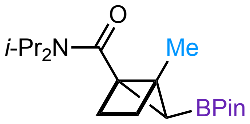

Synthesized according to **General procedure A** using **4a** (31 mg, 0.15 mmol, 1.0 equiv.) and 2-isopropoxy-4,4,5,5-tetramethyl-1,3,2-dioxaborolane (61.0 µL, 0.30 mmol, 2.0 equiv.). Purification using pentane/diethyl ether (75:25, v/v) as eluent afforded **5h** (36 mg, 0.11 mmol, 71%) as a light-yellow solid.

R_f = 0.3 (pentane/diethyl ether 7:3, v/v); **^{1}H NMR** (500 MHz, C_6D_6) δ_H 4.13 (br, 1H), 3.02 (br, 1H), 2.13 (td, *J* = 10.1, 3.3 Hz, 1H), 1.81 (s, 3H), 1.77 (td, *J* = 10.2, 3.5 Hz, 1H), 1.70–0.71 (m, 26H), 0.59 (s, 1H); **^{13}C NMR** (126 MHz, C_6D_6) δ_C 170.4, 82.7, 48.0, 45.7, 36.2, 35.37, 35.35, 29.7, 28.2, 25.3, 25.1, 21.1, 20.9, 16.3; **^{11}B NMR** (161 MHz, C_6D_6) δ 31.01; **IR** ν_{max} (cm^{-1}): 2974, 1638, 1427, 1351, 1313, 1137, 1042; **HRMS** (ESI+) calculated for $C_{19}H_{35}BNO_3$ $[M+H]^+$ 336.2705, found 336.2706.

(1*R,3*R**)-3-cyclohexyl-*N*,*N*-diisopropyl-3-(4,4,5,5-tetramethyl-1,3,2-dioxaborolan-2-yl)cyclopentane-1-carboxamide, 7**

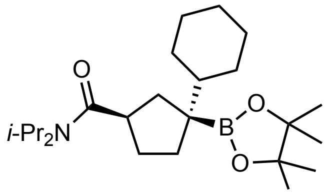

1 (20 mg, 0.10 mmol, 1.0 equiv.) was dissolved in THF (0.5 mL, 0.2 M) and TMEDA (20.0 μL, 0.12 mmol, 1.2 equiv.) was added. The resulting mixture was cooled to -45 °C, then *sec*-BuLi (0.10 mL, 1.2 M in cyclohexane, 0.12 mmol, 1.2 equiv.) was added dropwise and the solution was stirred at -45 °C for 30 min. 1-Cyclohexen-1-yl-boronic acid pinacol ester (28.0 μL, 0.12 mmol, 1.2 equiv.) was then added slowly and the solution was stirred at -45 °C for 30 min, then brought to ambient temperature and stirred for a further 30 min. The mixture was cooled to 0 °C and HCl (0.20 mL of a 1 M solution in diethyl ether, 0.20 mmol, 2.0 equiv.) was added. The resulting mixture was stirred for 30 min at ambient temperature, then it was diluted with water (0.5 mL) and diethyl ether (0.5 mL). The layers were separated and the aqueous layer was extracted with diethyl ether (0.5 mL). The organic layers were combined, dried ($MgSO_4$), filtered and concentrated *in vacuo*. Purification using silica and pentane/diethyl ether (97:3, v/v) as eluent afforded **7** (20 mg, 49 μmol, 49%) as a clear liquid.

R_f = 0.3 (pentane/diethyl ether 97:3, v/v); **^{1}H NMR** (600 MHz, $CDCl_3$) δ_H 4.03 (br., 1H), 3.45 (br, 1H), 2.74 (tt, *J* = 9.7, 7.6 Hz, 1H), 2.03 (m, 1H), 1.95 (dd, *J* = 13.0, 7.8 Hz, 1H), 1.88 (m, 1H), 1.75–1.63 (m, 7H), 1.39–1.33 (m, 7H), 1.24 (d, *J* = 4.3 Hz, 12H), 1.21 (d, *J* = 6.7 Hz, 4H), 1.18 (d, *J* = 6.7 Hz, 4H), 1.17–0.98 (m, 4H); **^{13}C NMR** (151 MHz, $CDCl_3$) δ_C 174.2, 83.0, 47.7, 46.9, 45.4, 45.3, 42.8, 37.3, 33.5, 31.0, 30.3, 27.1, 27.0, 26.9, 24.9, 24.7, 21.3, 21.8, 20.6; **IR** ν_{max} (cm^{-1}): 2975, 2930, 1643, 1408, 1386, 1301,1146; **HRMS** (ESI+): calculated for $C_{24}H_{45}{}^{10}BNO_3$ $[M+H]^+$ 406.3487, found 406.3492.

6. Enantioselective synthesis of (-)-1

(1*S*,3*S*)-3-hydroxy-*N*,*N*-diisopropylcyclopentane-1-carboxamide, (+)-2

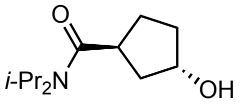

To a stirred solution of (-)-(Ipc)$_2$BH (2.67 g, 9.32 mmol, 1.3 equiv., prepared according to a modified literature procedure[2]) in anhydrous THF (6 mL) at −35 °C was added *N,N*-diisopropylcyclopent-3-ene-1-carboxamide **S1** (1.40 g, 7.17 mmol, 1.0 equiv.) as a solution in THF (1 mL) dropwise. The mixture was stirred at this temperature for 48 h, after which time water (6 mL) was added. The reaction flask was allowed to warm to room temperature, and sodium perborate tetrahydrate (2.21 g, 14.3 mmol, 2.0 equiv.) was added. The mixture was stirred overnight (16 h), then brine (10 mL) was added. The aqueous phase was extracted with ethyl acetate (3 × 10 mL), and the combined organic extracts were washed with brine (10 mL), dried over $MgSO_4$, and concentrated *in vacuo*. The residue was purified by flash column chromatography on silica gel using pentane/ethyl acetate (3:7, v/v) as eluent to afford **(+)-2** (980 mg, 4.59 mmol, 64%) as a clear viscous liquid.

$[\alpha]_D^{20}$ = +3.3 (*c* 0.8, $CHCl_3$), *ee* 97%.

All other spectroscopic data were identical to that of **(±)-2** as prepared above.

HPLC method: Daicel ChiralPak IC column, 25 °C, 1.0 mL/min, eluent: 90:10 *n*-hexane:isopropanol. HPLC chromatograms for **2:** racemic sample (top), enantioenriched (bottom).

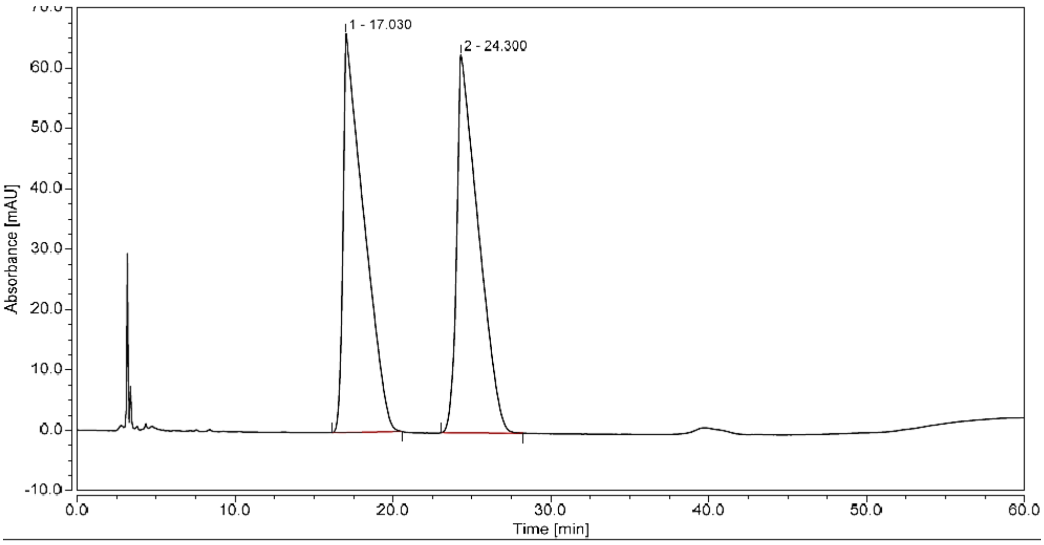

Peak	Ret. Time (min)	Area (mAU*min)	Relative Area (%)
1	17.03	100.07	50.2
2	24.30	99.19	49.8

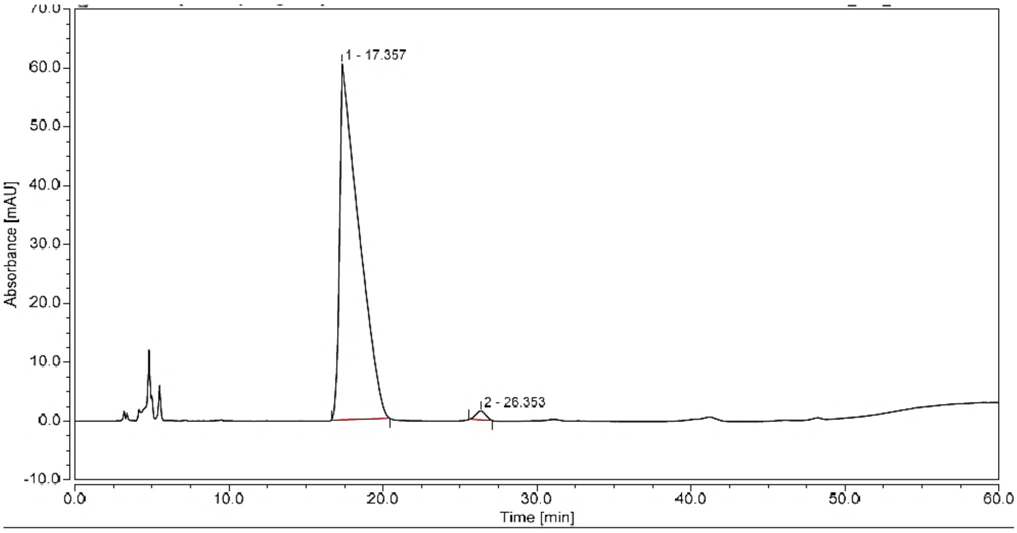

Peak	Ret. Time (min)	Area (mAU*min)	Relative Area (%)
1	17.36	87.22	98.8
2	26.35	1.09	1.2

(1*S*,3*S*)-3-((4-bromobenzyl)oxy)-*N*,*N*-diisopropylcyclopentane-1-carboxamide, (+)-9

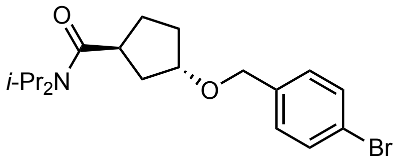

NaH (60% w/w in mineral oil, 5 mg, 0.13 mmol, 1.3 equiv.) was suspended in dry DMF (0.6 mL) at ambient temperature. To this stirred suspension was added a solution of **(+)-2** (21 mg, 0.1 mmol, 1.0 equiv.) in dry DMF (0.2 mL). After 15 min, a solution of 1-bromo-4-(bromomethyl)benzene (32 mg, 0.13 mmol, 1.3 equiv.) in DMF (0.2 mL) was added and the solution was stirred a further 1 h at rt. After this time, the reaction mixture was quenched by addition of water (2 mL), and then diluted with EtOAc (2 mL). The organic layer was washed with water (4 x 2 mL), brine (2 mL), dried over $MgSO_4$, filtered and concentrated *in vacuo*. The residue was purified by flash column chromatography on silica gel using pentane/Et_2O (10:1, v/v) as eluent to afford **(+)-9** (29 mg, 76 μmol, 76%) as a pale yellow solid. A crystal suitable for single crystal X-ray diffraction analysis was obtained by recrystallization from dichloromethane/hexane.

$\mathbf{R}_f$ = 0.2 (pentane/Et_2O 10:1, v/v); $[\alpha]_D^{20}$ = +8.2 (*c* 0.3, $CHCl_3$), *ee* 96%.

^{1}H NMR (400 MHz, $CDCl_3$) δ 7.48–7.44 (m, 2H), 7.23–7.19 (m, 2H), 4.42 (d, *J* = 3.2 Hz, 2H), 4.14–4.09 (m, 1H), 4.09–4.02 (m, 1H), 3.48 (s, 1H), 3.09 (p, *J* = 8.1 Hz, 1H), 2.07 (ddd, *J* = 14.4, 8.8, 5.6 Hz, 1H), 2.01–1.87 (m, 2H), 1.83–1.72 (m, 2H), 1.36 (d, *J* = 6.8 Hz, 6H), 1.19 (dd, *J* = 6.8, 3.7 Hz, 6H); **^{13}C NMR** (101 MHz, $CDCl_3$) δ_C 174.3, 138.0, 131.5, 129.3, 121.4, 81.2, 70.1, 48.0, 45.7, 40.9, 36.4, 32.0, 28.1, 21.3, 20.8, 20.8; **IR** ν_{max} (cm^{-1}): 2968, 1638, 1488, 1441, 1371, 1298; **HRMS** (ESI+): calc. for $C_{19}H_{28}BrNO_2Na$ $[M+Na]^+$ 404.1196, found 404.1208.

HPLC method: Daicel ChiralPak IA column, 5 °C, 1.0 mL/min, eluent: 98.5:1.5 *n*-hexane:isopropanol. HPLC chromatograms for **9**, racemic (top) and enantioenriched (bottom).

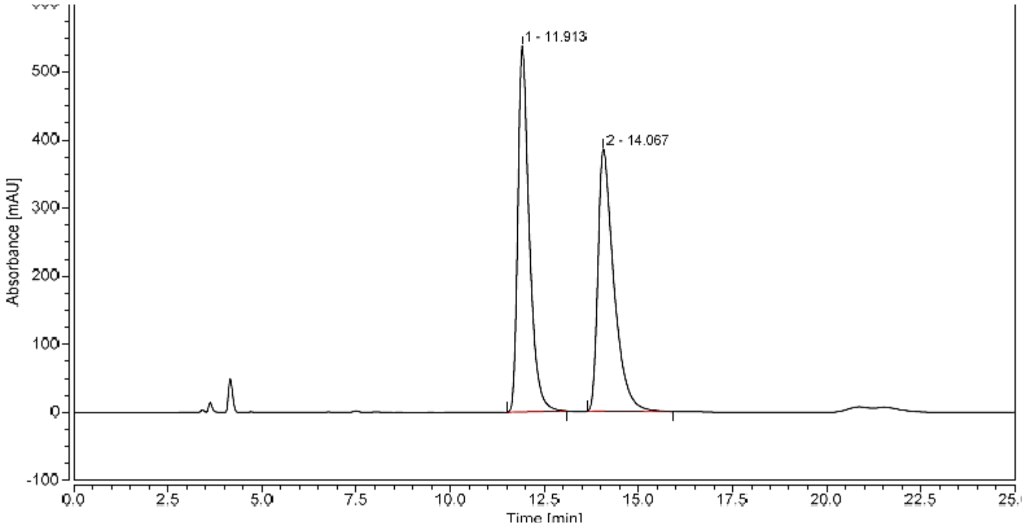

Peak	Ret. Time (min)	Area (mAU*min)	Relative Area (%)
1	11.91	189.13	55.1
2	14.07	188.44	49.9

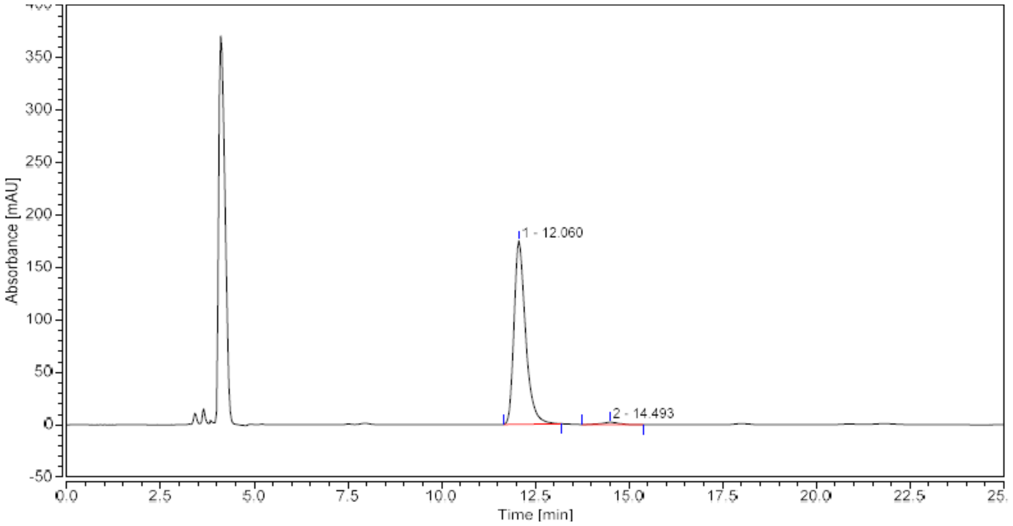

Peak	Ret. Time (min)	Area (mAU*min)	Relative Area (%)
1	12.06	62.58	98.2
2	14.49	1.14	1.80

(1*S*,3*S*)-3-chloro-*N*,*N*-diisopropylcyclopentane-1-carboxamide, (+)-3

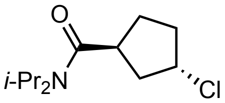

A solution of **(+)-2** (720 mg, 3.38 mmol, 1.0 equiv.) in pyridine (5 mL) was cooled to 0 °C, and $PhSO_2Cl$ (0.56 mL, 4.39 mmol, 1.3 equiv.) was added dropwise. The mixture was stirred at ambient temperature (23 °C) for 8 h, then it was diluted with CH_2Cl_2 (5 mL), washed with NH_4Cl (sat., aq., 3 × 5 mL) and brine (5 mL), dried over $MgSO_4$, filtered and concentrated *in vacuo*. The residue was purified by flash column chromatography on silica gel using pentane/diethyl ether (8:2, v/v) as eluent to afford **(+)-3** (395 mg, 1.70 mmol, 50%) as a yellow liquid.

$[\alpha]_D^{20}$ =+7.7 (*c* 1.9, $CHCl_3$); *ee* 97%.

All other spectroscopic data were identical to that of **(±)-3** as prepared above.

HPLC method: Daicel ChiralPak IC column, 5 °C, 1.0 mL/min, eluent: 97:3 *n*-hexane:isopropanol. HPLC Chromatogram for **3,** racemic top, enantioenriched bottom.

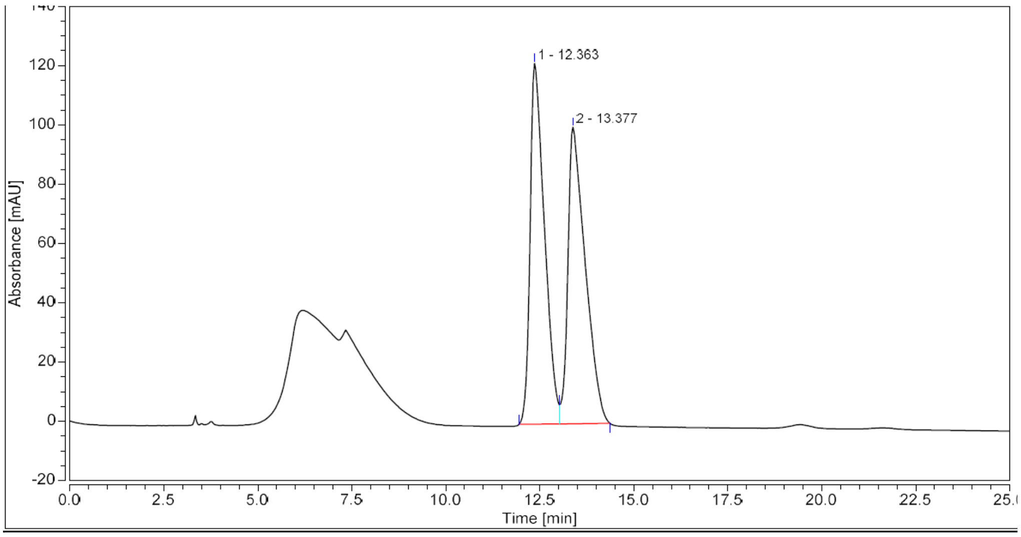

Peak	Ret. Time (min)	Area (mAU*min)	Relative Area (%)
1	12.36	53.75	50.1
2	13.38	53.58	49.9

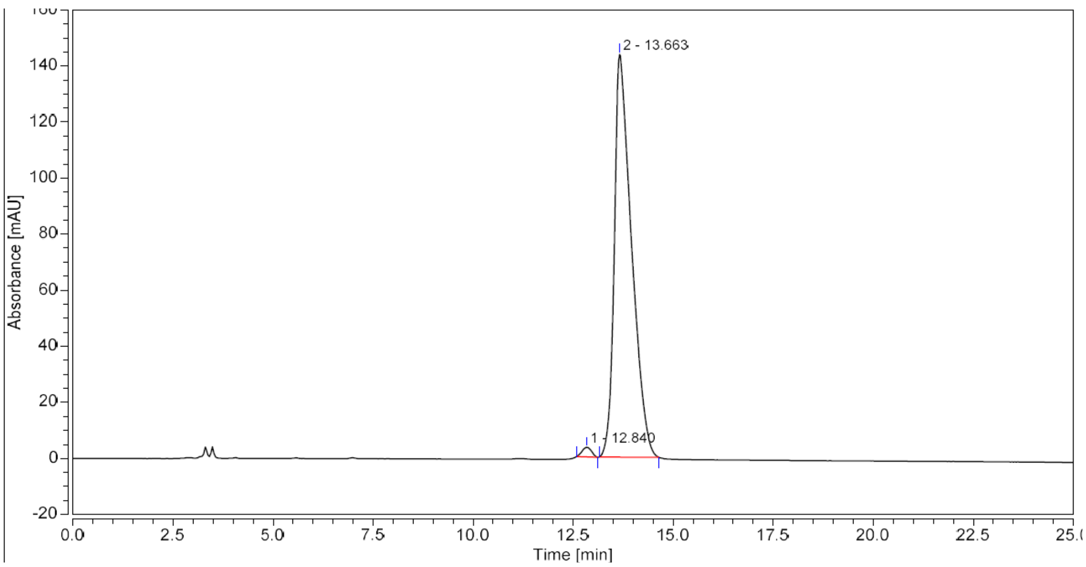

Peak	Ret. Time (min)	Area (mAU*min)	Relative Area (%)
1	12.84	0.92	1.3
2	13.66	71.39	98.7

(1*R*,4*R*)-*N*,*N*-diisopropylbicyclo[2.1.0]pentane-1-carboxamide, (-)-1

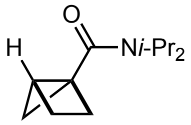

(+)-3 (300 mg, 1.29 mmol, 1.0 equiv.) was dissolved in THF (2 mL) and cooled to 0 °C. LiHMDS (1.9 mL of 1.0 M solution in THF, 1.94 mmol, 1.5 equiv.) was added dropwise at 0 °C and the solution was stirred at ambient temperature (23 °C) overnight. Then, the mixture was diluted with NH_4Cl (sat., aq., 10 mL) and diethyl ether (10 mL), the organic layer was washed with brine (10 mL), dried with $MgSO_4$ and concentrated *in vacuo*. The crude mixture was purified by flash column chromatography on silica gel using pentane/diethyl ether (7:3 v/v) as eluent to afford **(-)-1** (141 mg, 0.72 mmol, 56%) as a yellow solid.

$[\alpha]_D^{20}$ = -47.8 (*c* 0.5, $CHCl_3$), *ee* 96%.

All other spectroscopic data were identical to that of **(±)-1** as prepared above.

HPLC method: Daicel ChiralPak IC column, 5 °C, 1.0 mL/min, eluent: 98:2 *n*-hexane:isopropanol. HPLC Chromatogram for **1** racemic top, enantioenriched bottom

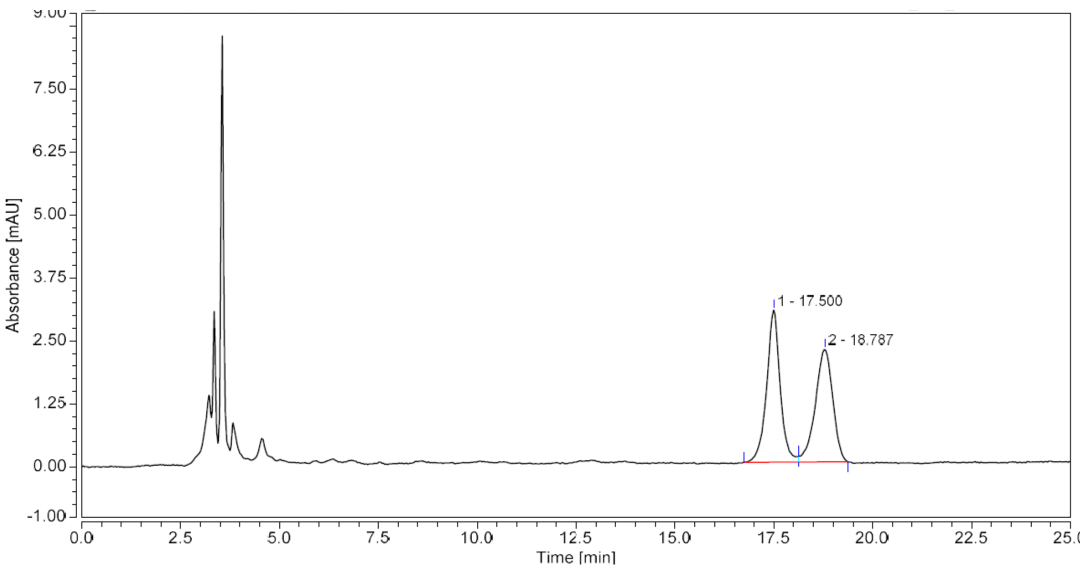

Peak	Ret. Time (min)	Area (mAU*min)	Relative Area (%)
1	17.50	1.20	51.3
2	18.79	1.14	48.7

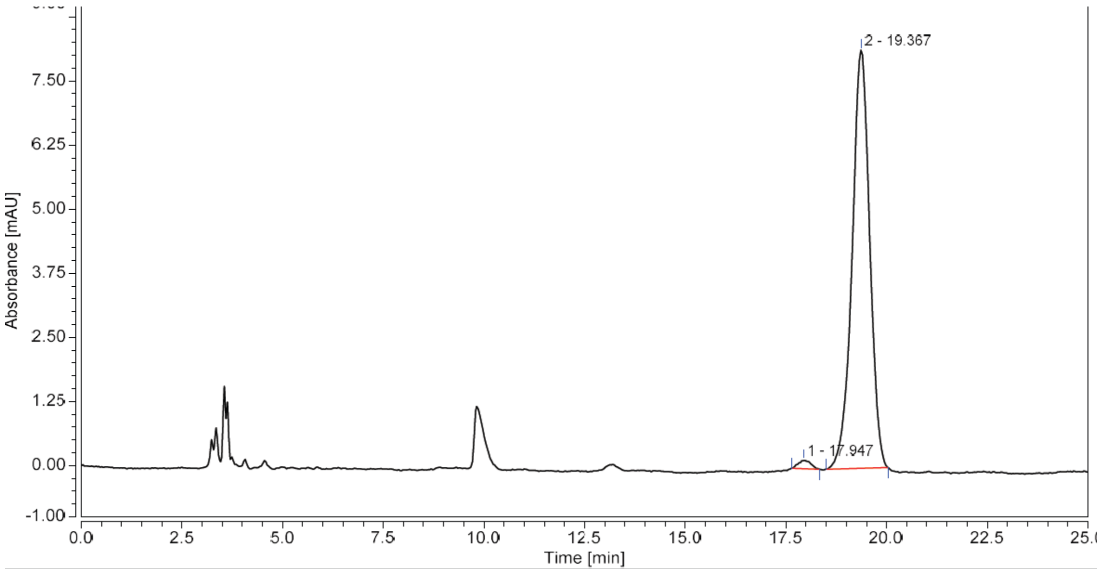

Peak	Ret. Time (min)	Area (mAU*min)	Relative Area (%)
1	17.95	0.06	2.0
2	19.37	4.13	98.0

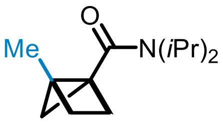

(-)-**4a**

Compound (-)-**1** (20 mg, 0.10 mmol, 1.0 equiv.) was dissolved in THF (1.0 mL) and TMEDA (18 μL, 0.12 mmol, 1.2 equiv.) was added. The resulting mixture was cooled to –45 °C, *sec*-BuLi (0.12 mL, 1.05 M in hexane, 0.12 mmol, 1.2 equiv.) was added dropwise and the solution was stirred at –45 °C for 30 min. MeI (13 μL, 0.20 mmol, 2.0 equiv.) was then added slowly and the solution was stirred at –45 °C for 30 min, then brought to ambient temperature and stirred for a further 30 min. $NaHCO_3$ (aq) (0.5 mL) was then added dropwise and the mixture was diluted with diethyl ether (1.0 mL). The aqueous layer was extracted with diethyl ether (1.0 mL × 3). The organic layers were combined, washed with brine, dried ($MgSO_4$), then filtered and concentrated *in vacuo*. The crude mixture was purified by flash column chromatography using silica gel and pentane/diethyl ether (8:2, v/v) and isolated as a colourless oil. Yield: 14 mg, 67 μmol, 65%. $[\alpha]_D^{20}$ =-4.8 (*c* 0.2, $CHCl_3$), *ee* 94%.

HPLC method: Daicel ChiralPak IC column, 5 °C, 1.0 mL/min, eluent: 98:2 *n*-hexane:isopropanol. HPLC Chromatogram for **1** racemic top, enantioenriched bottom

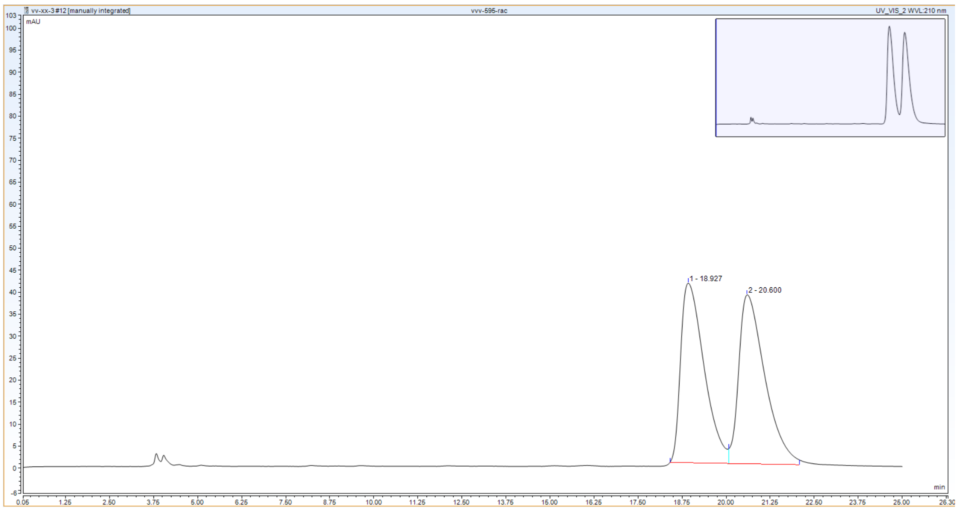

Peak	Ret. Time (min)	Area (mAU*min)	Relative Area (%)
1	18.93	31.52	48.9
2	20.60	33.00	51.15

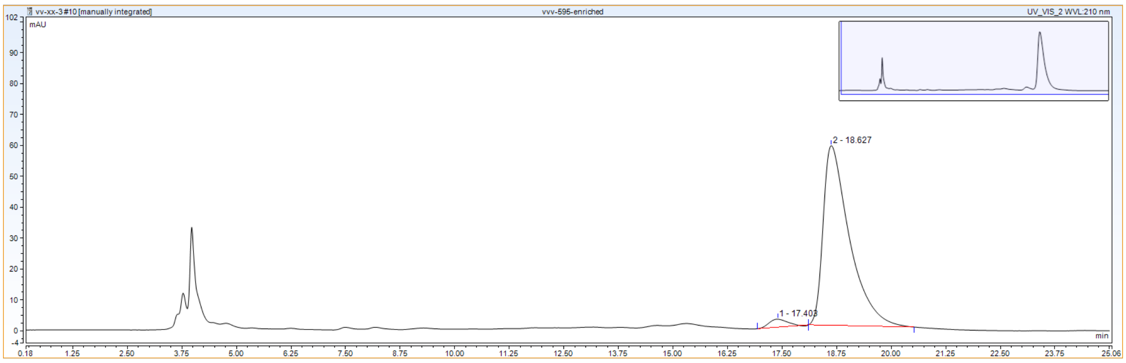

Peak	Ret. Time (min)	Area (mAU*min)	Relative Area (%)
1	17.40	1.23	2.93
2	18.63	40.70	97.1

7. Unsuccessful attempts

Attempts at synthesising the following compounds using **General procedure A** proved unsuccessful, resulting in complicated reaction mixtures and possible ring opening.

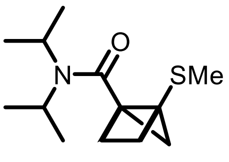

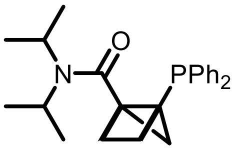

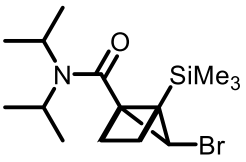

8. NMR spectra

^{1}H NMR (500 MHz, $CDCl_3$) spectrum of **1**

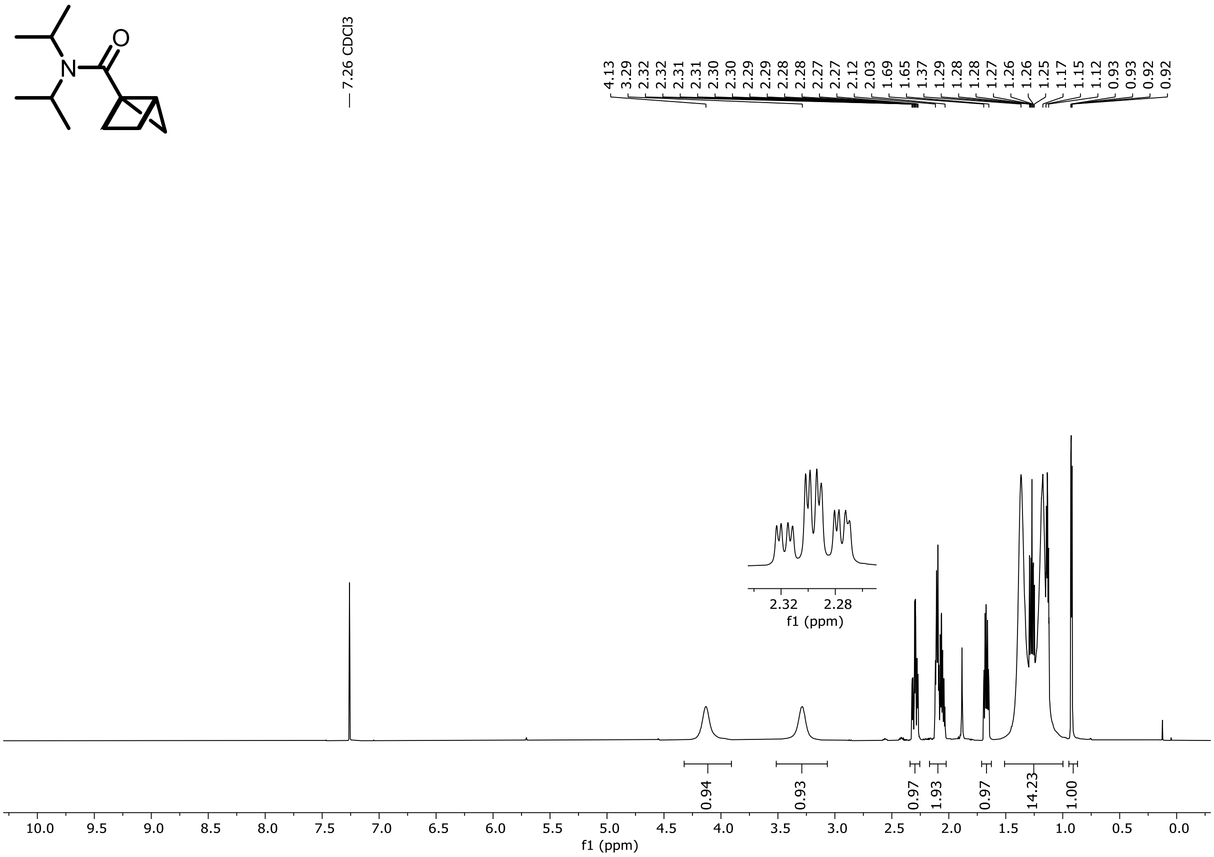

^{13}C NMR (126 MHz, $CDCl_3$) spectrum of **1**

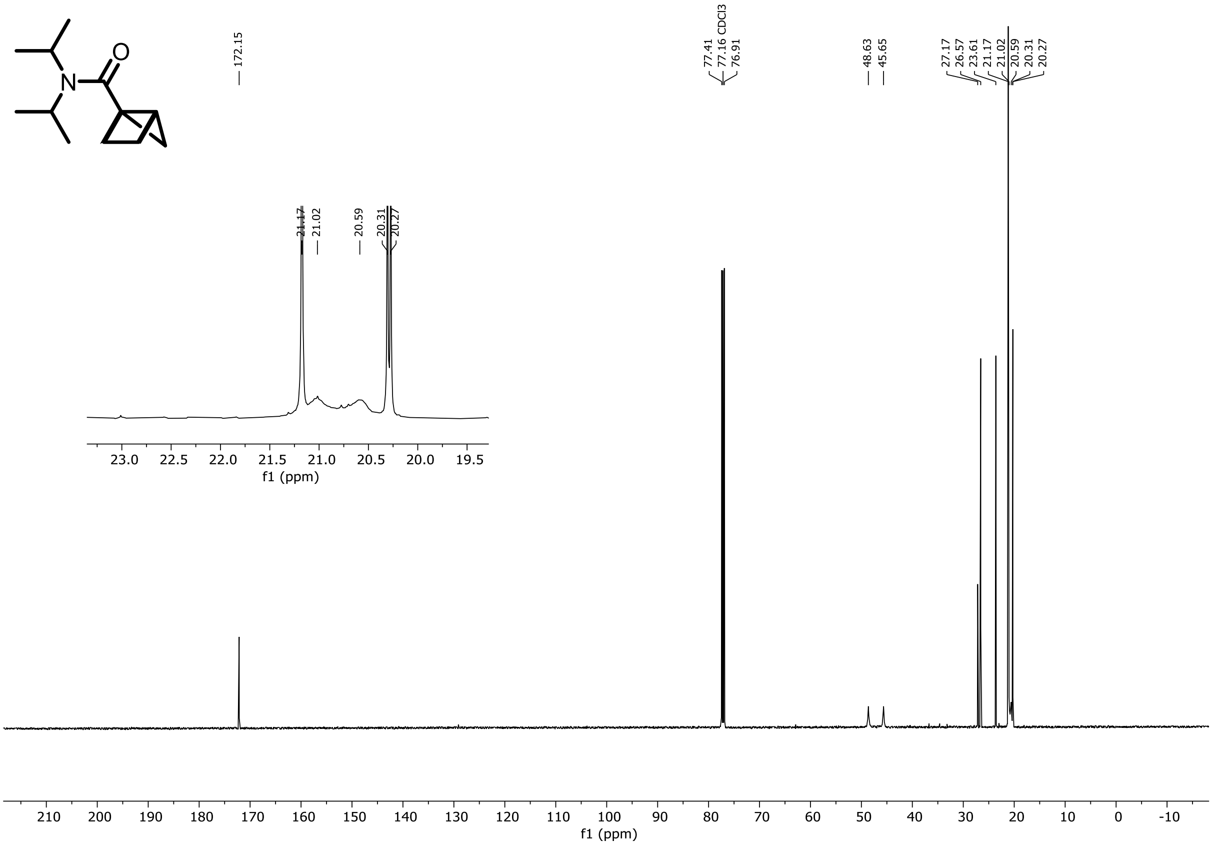

^{1}H - ^{1}H COSY (500 MHz, $CDCl_3$) spectrum of **1**

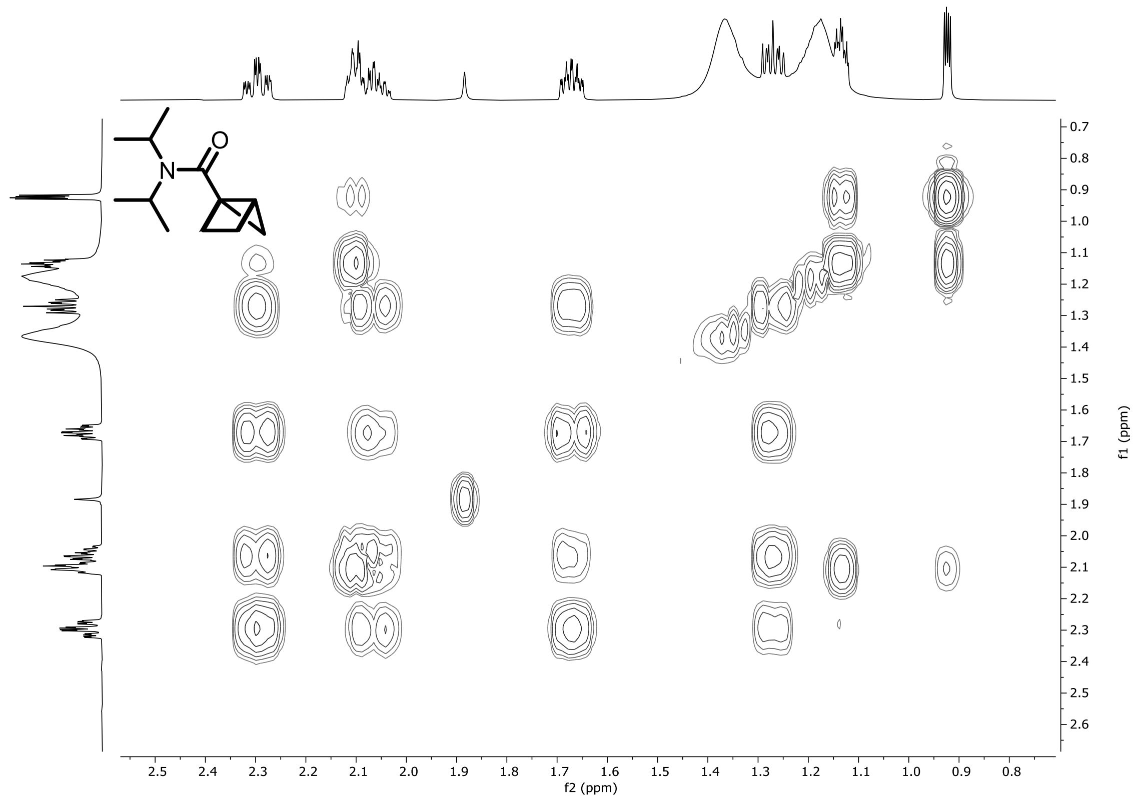

^{1}H – ^{13}C HSQC ($CDCl_3$) spectrum of **1**

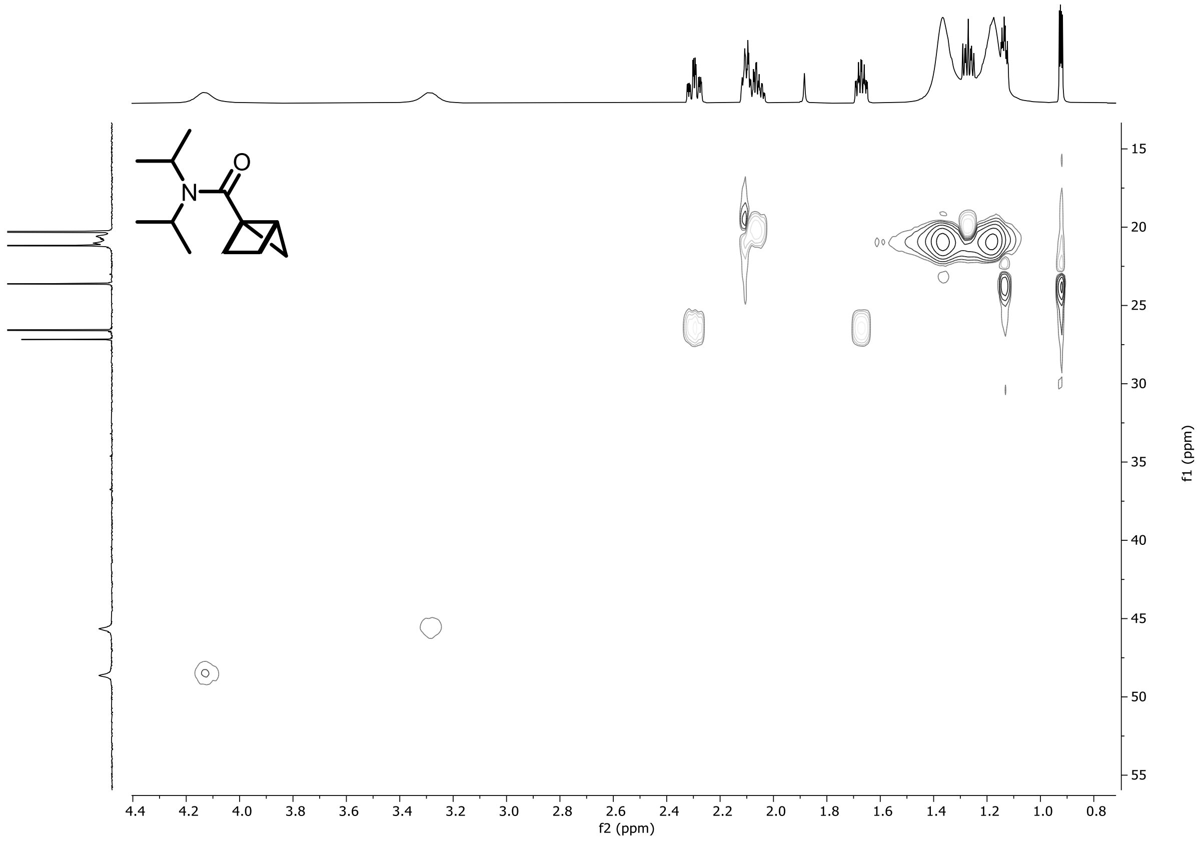

^{1}H NMR (400 MHz, $CDCl_3$) spectrum of **d-1**

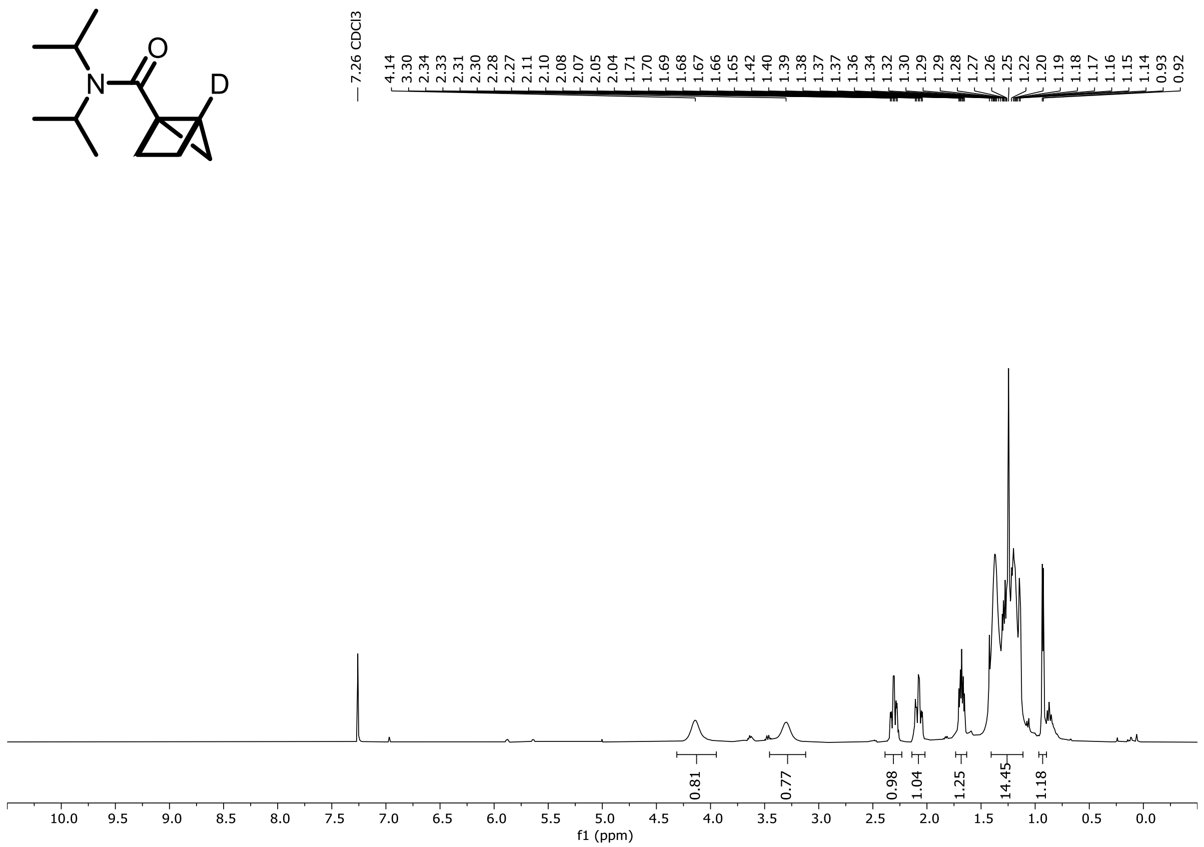

^{13}C NMR (101 MHz, C_6D_6) spectrum of **d-1**

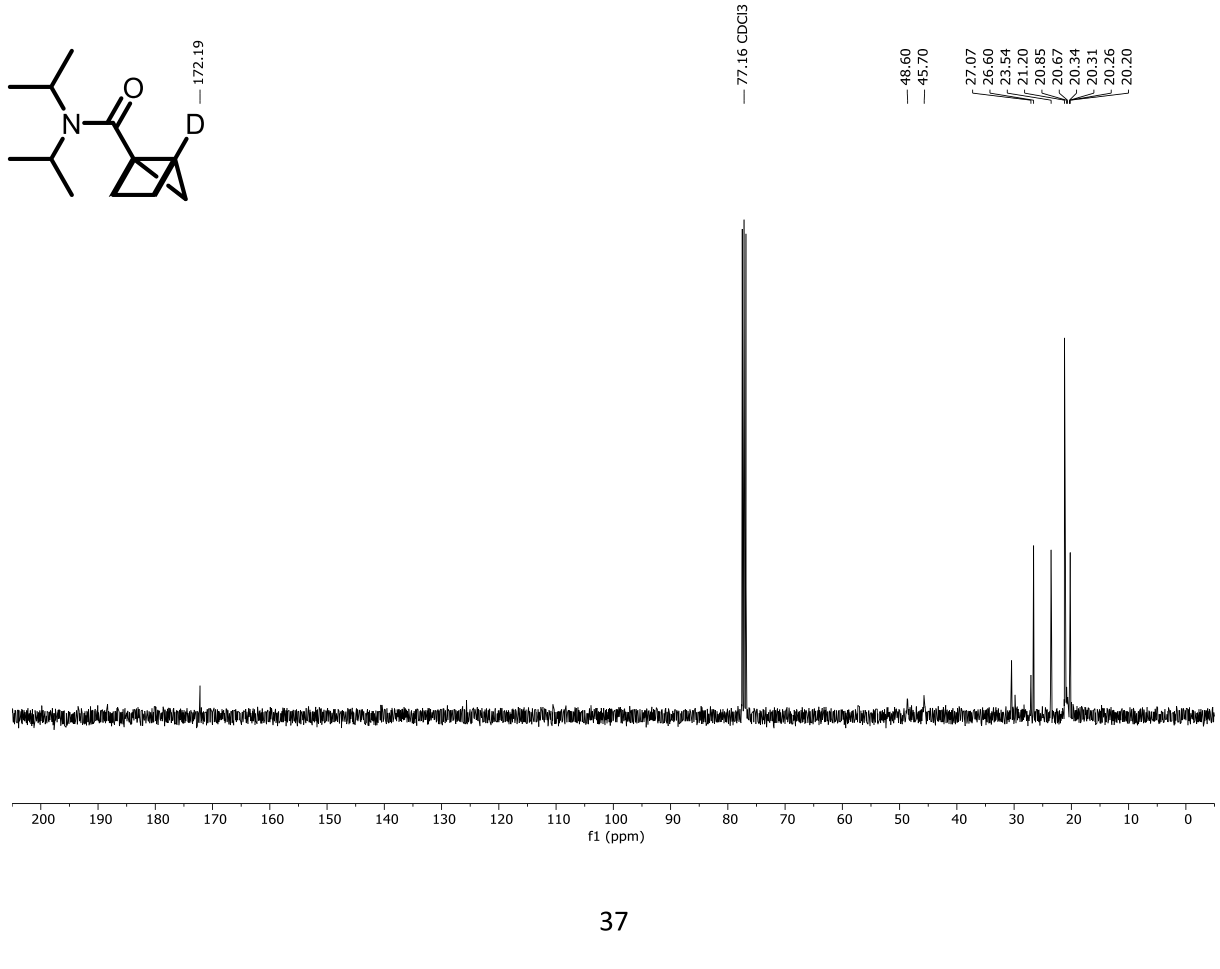

^{1}H NMR (500 MHz, CDCl$_3$) spectrum of **d-4a**

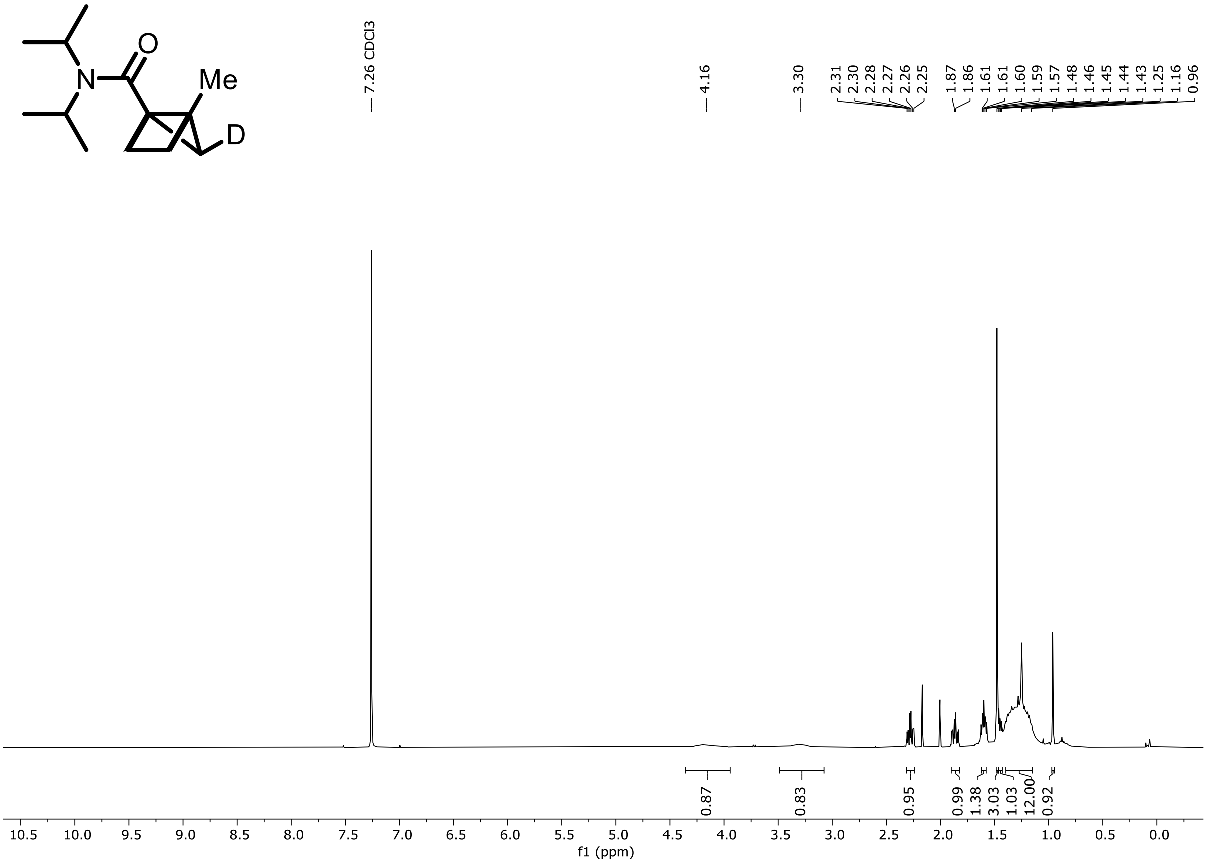

^{13}C NMR (126 MHz, CDCl$_3$) spectrum of **d-4a**

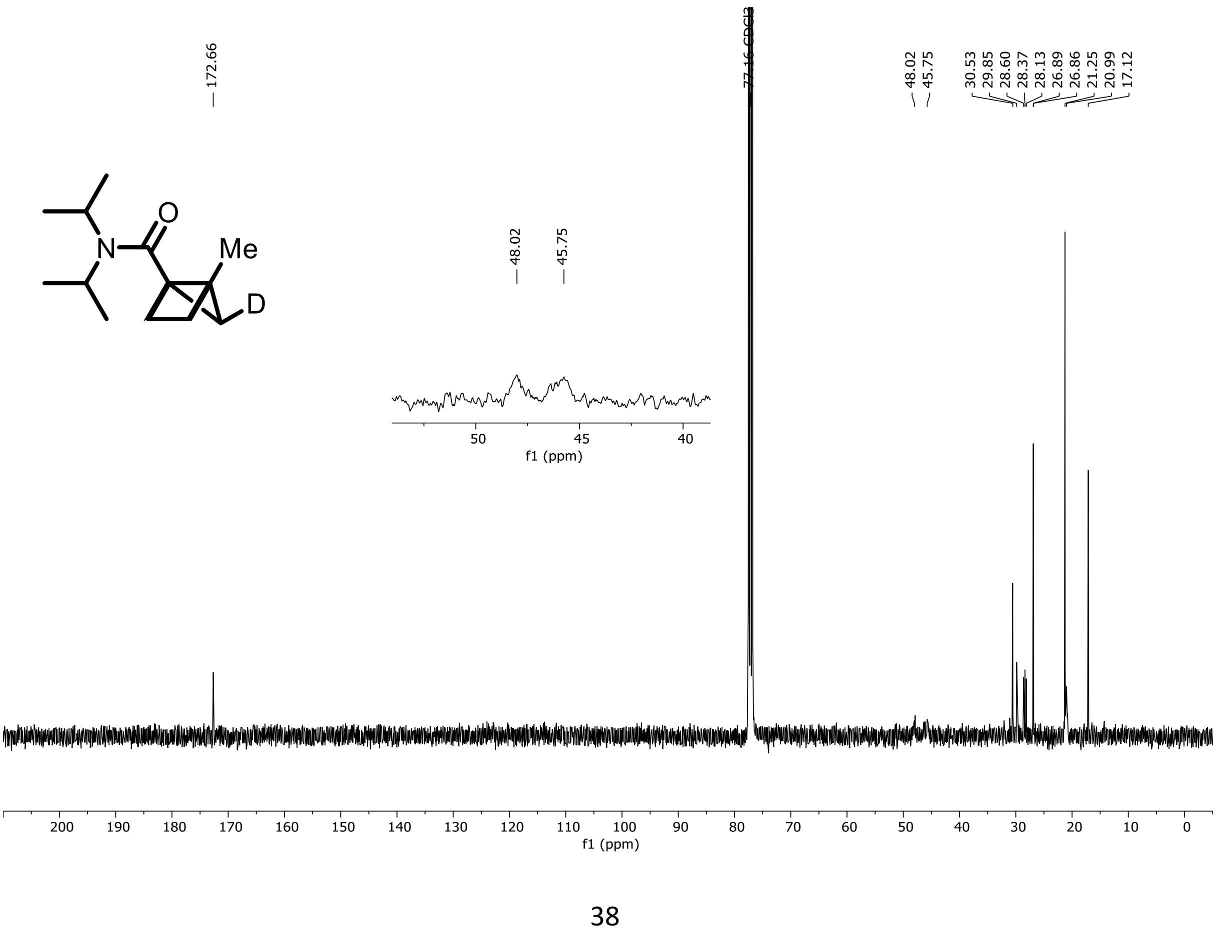

^{1}H NMR (500 MHz, $CDCl_3$) spectrum of *N,N*-diisopropylcyclopent-3-ene-1-carboxamide, **8**

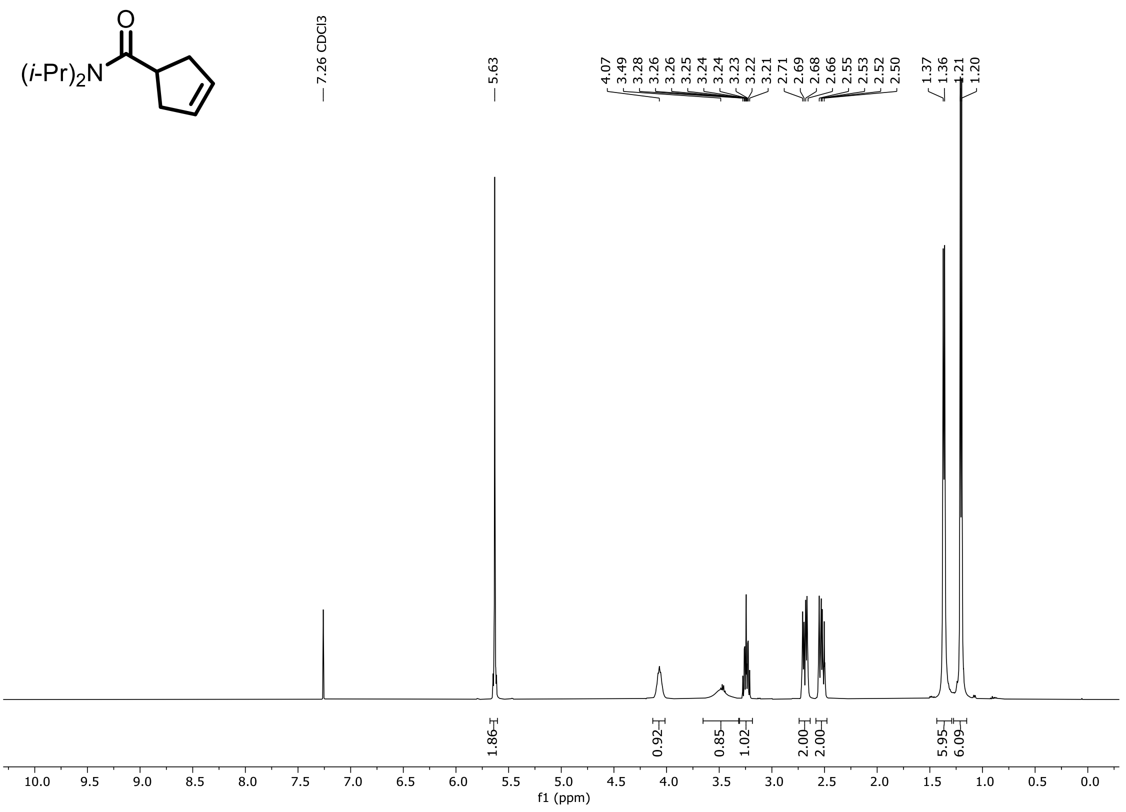

^{13}C NMR (126 MHz, $CDCl_3$) spectrum of *N,N*-diisopropylcyclopent-3-ene-1-carboxamide, **8**

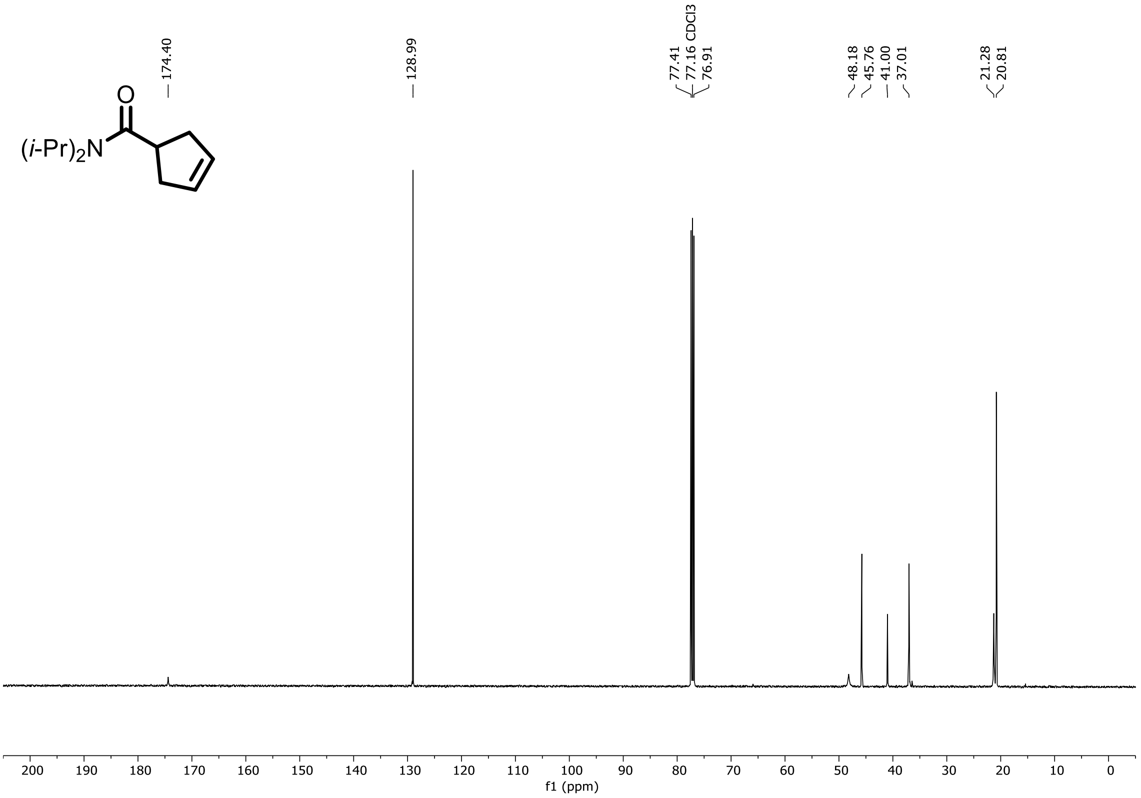

^{1}H NMR (500 MHz, $CDCl_3$) spectrum of **2**

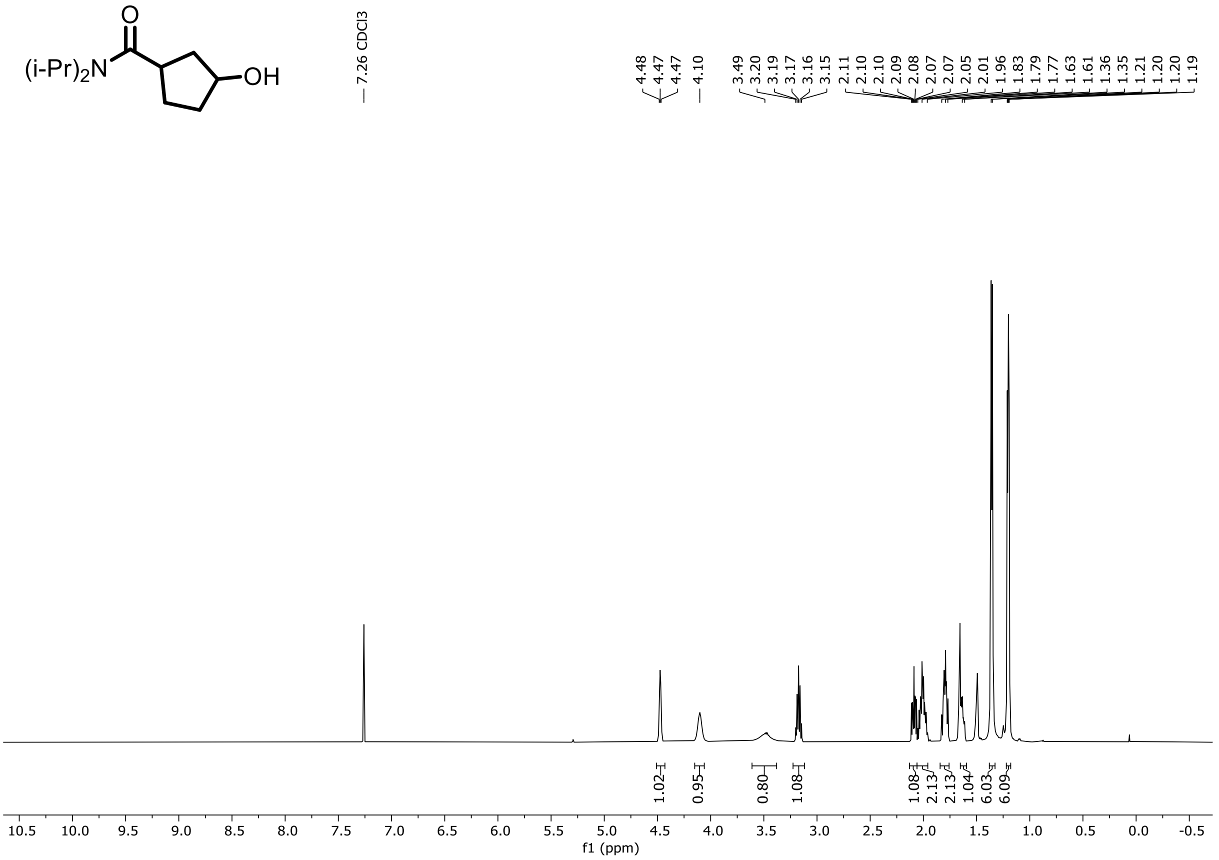

^{13}C NMR (126 MHz, $CDCl_3$) spectrum of **2**

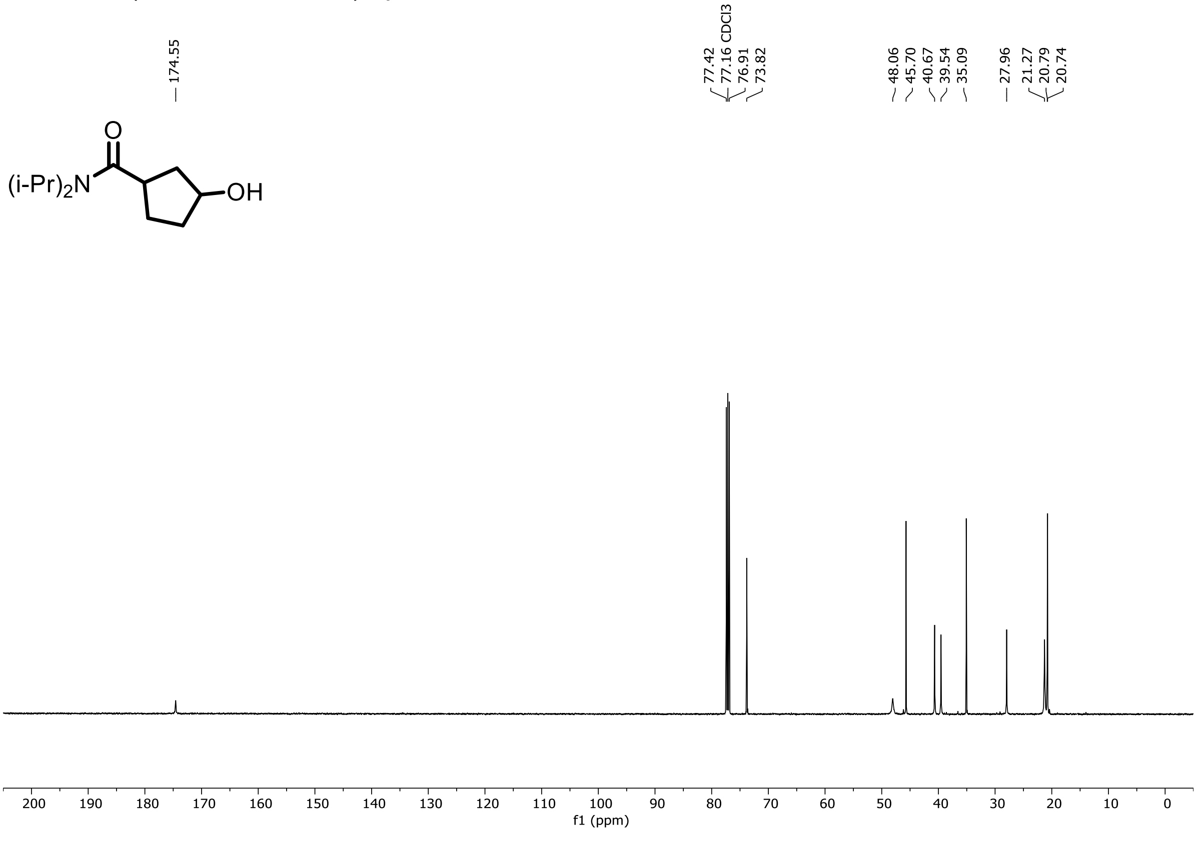

^{1}H NMR (500 MHz, $CDCl_3$) spectrum of **3**

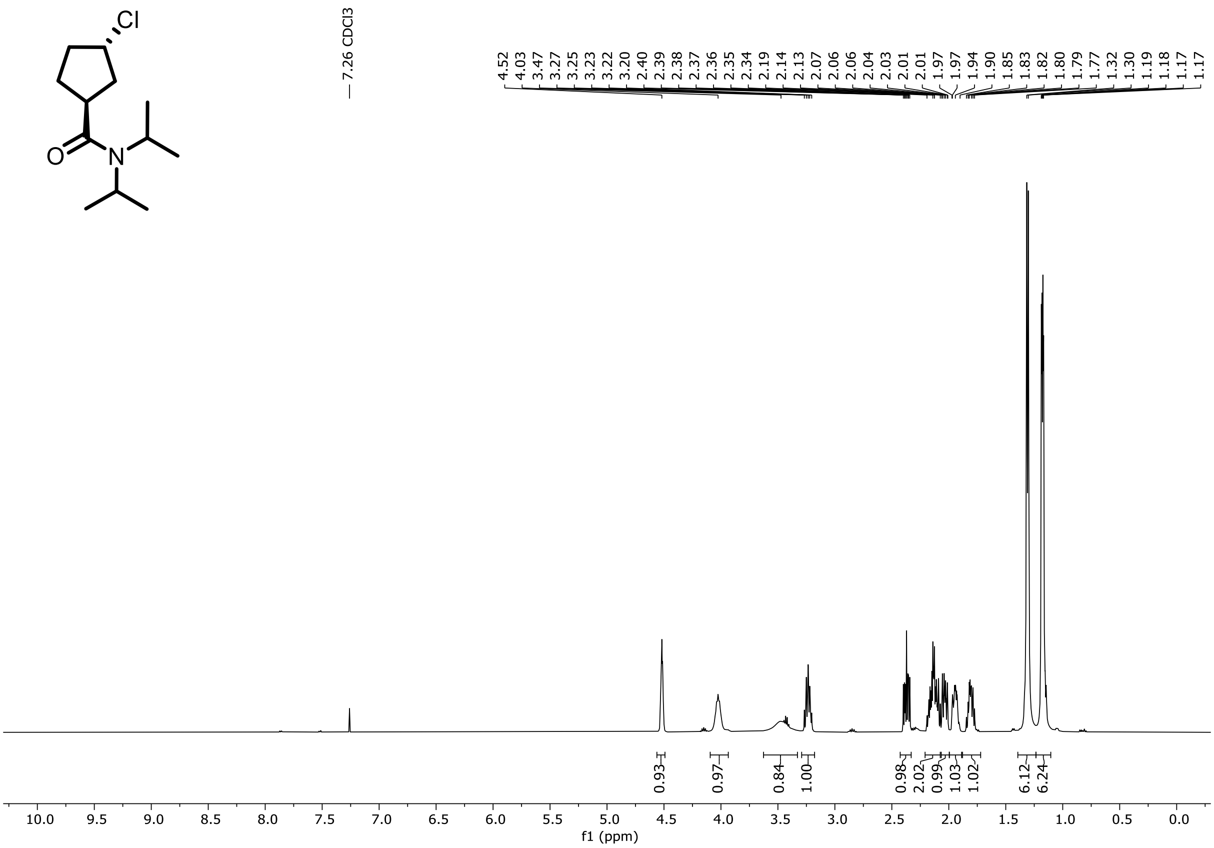

^{13}C NMR (126 MHz, $CDCl_3$) spectrum of **3**

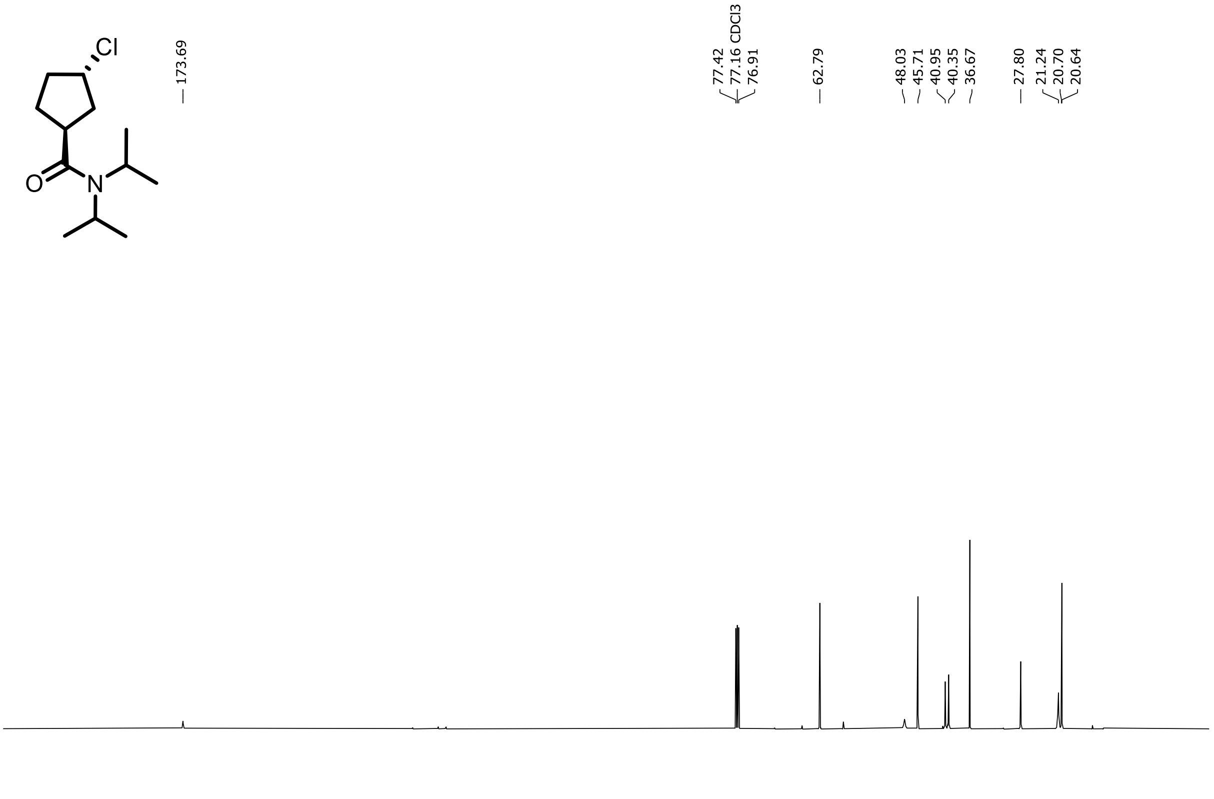

NOESY (600 MHz, $CDCl_3$) spectrum of **3**

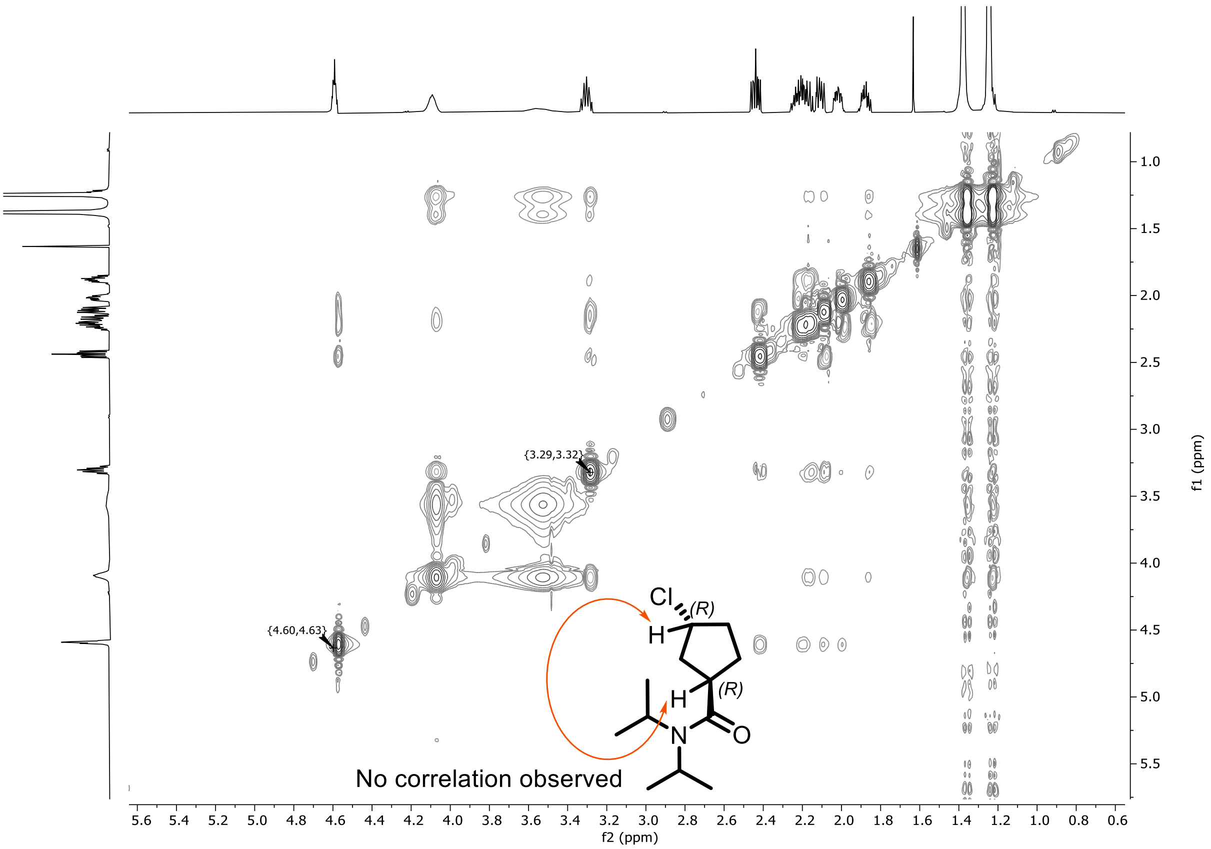

^{1}H NMR (400 MHz, $CDCl_3$) spectrum of **3'**

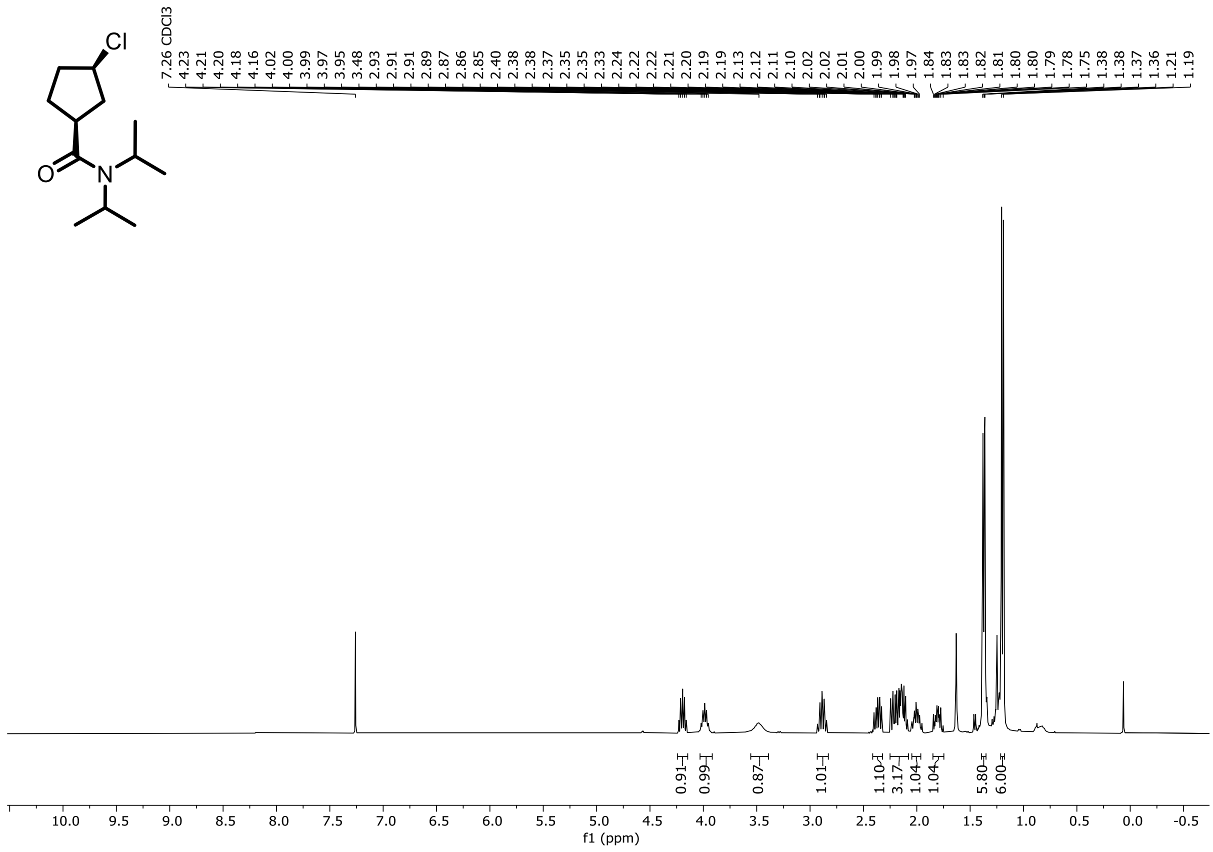

^{13}C NMR (101 MHz, $CDCl_3$) spectrum of **3'**

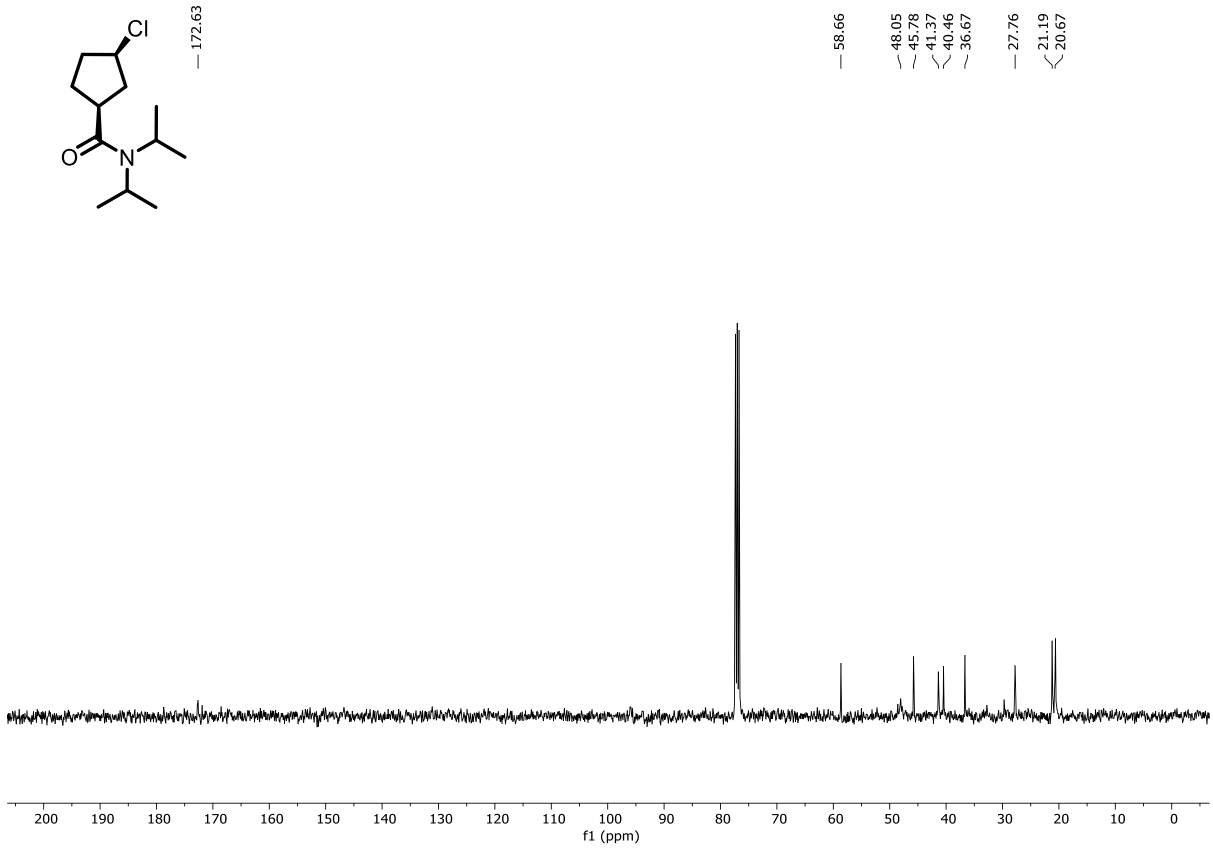

NOESY (600 MHz, $CDCl_3$) spectrum of **3'**

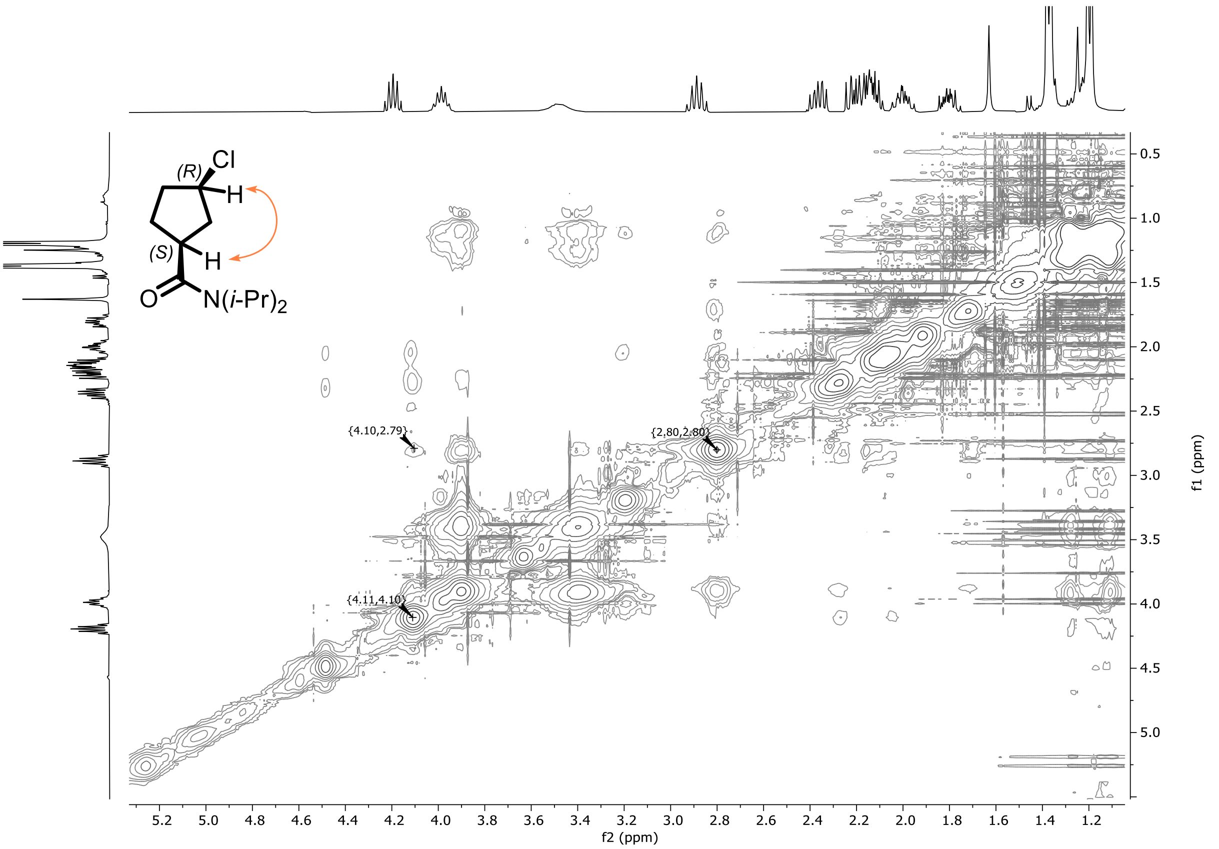

^{1}H NMR (500 MHz, $CDCl_3$) spectrum of **4a**

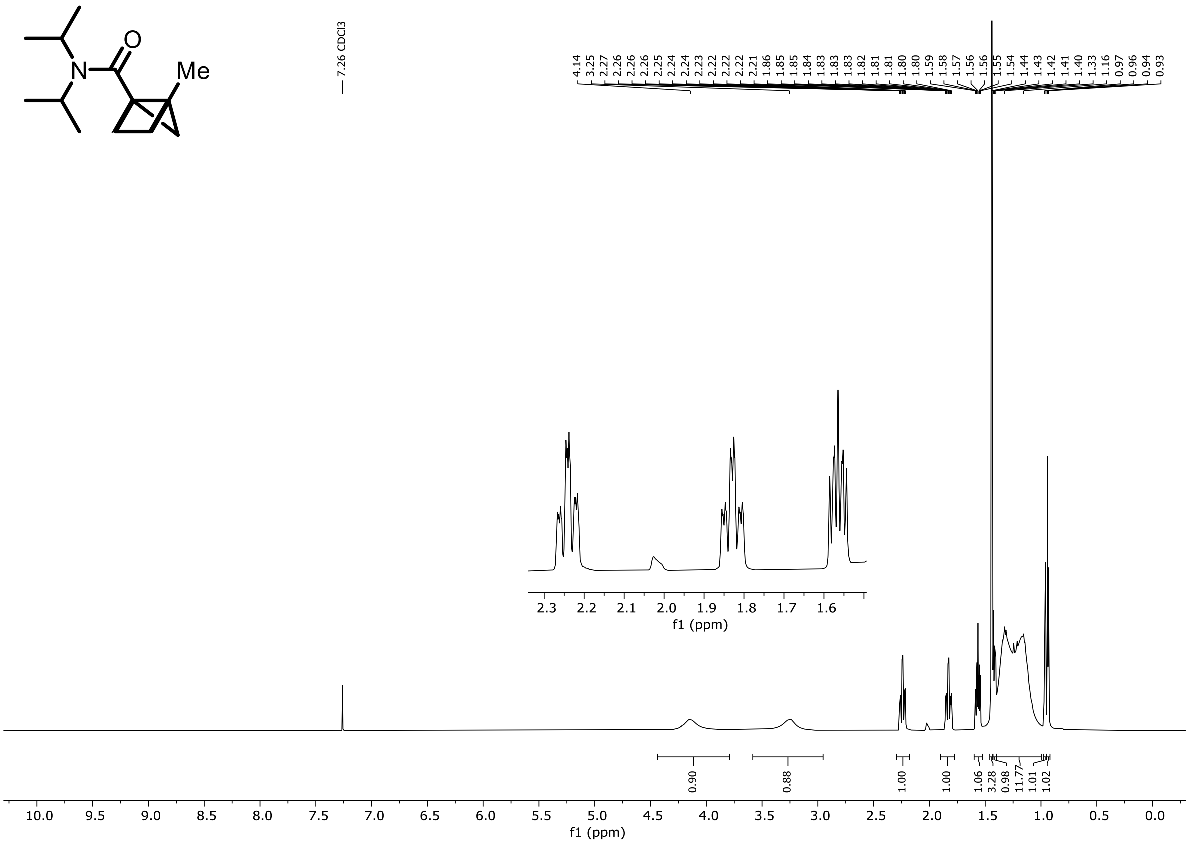

^{13}C NMR (126 MHz, $CDCl_3$) spectrum of **4a**

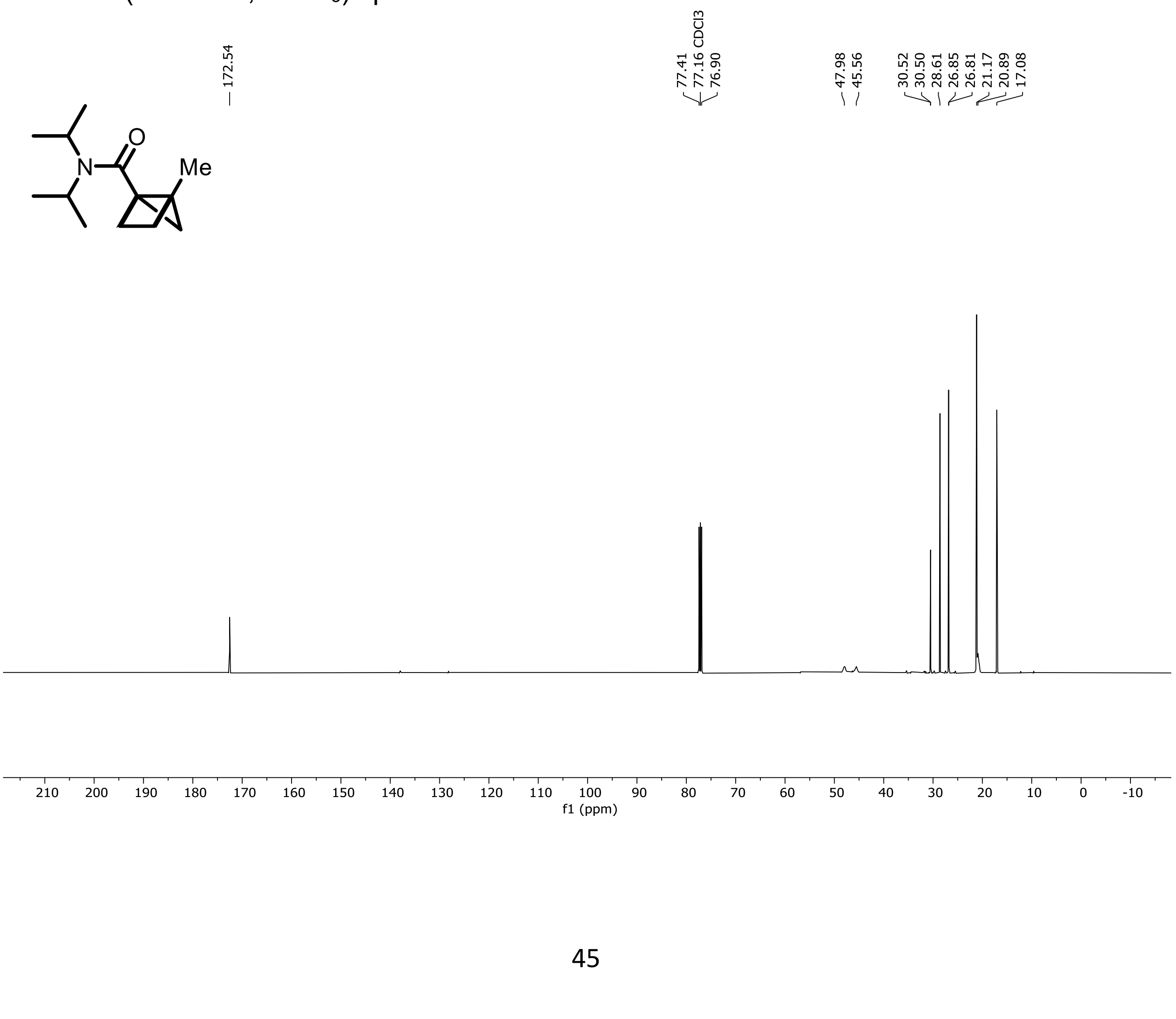

^1H NMR (400 MHz, C_6D_6) spectrum of **4b**

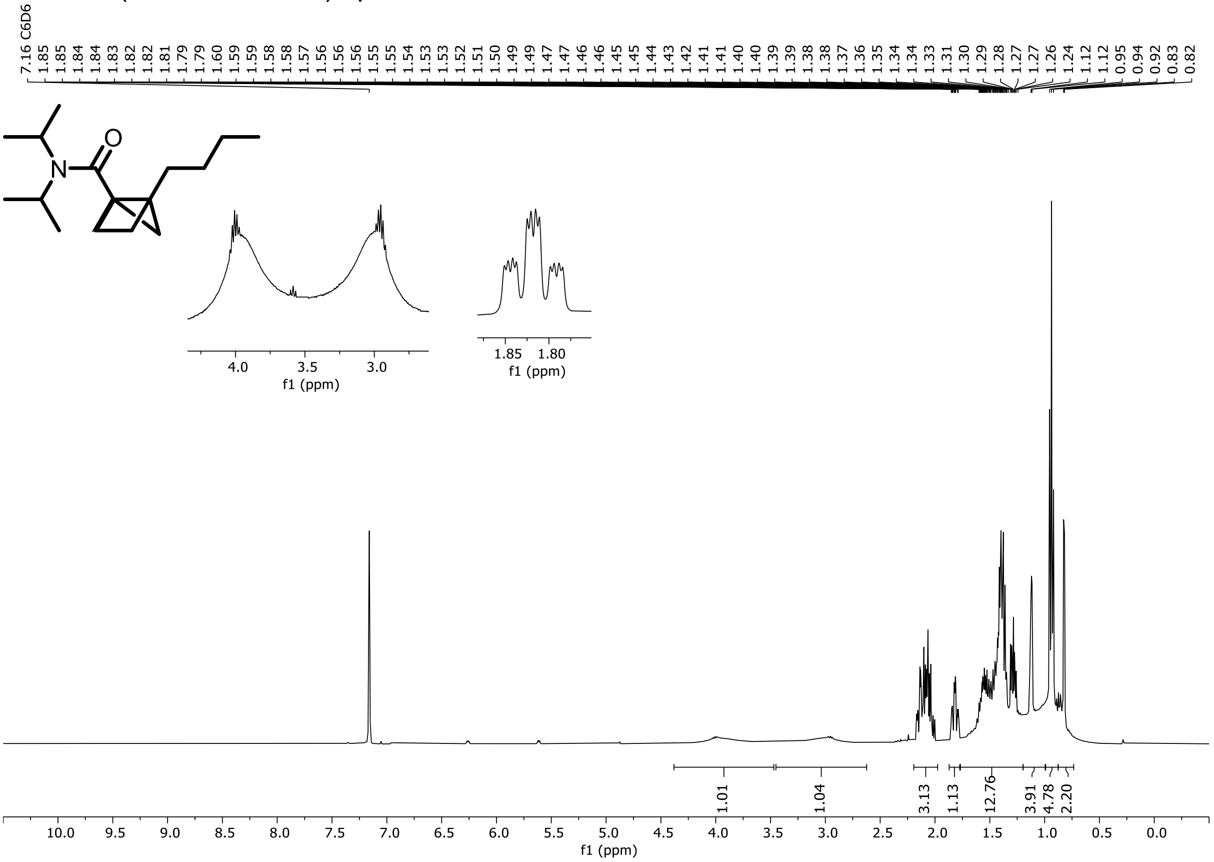

^{13}C NMR (101 MHz, C_6D_6) spectrum of **4b**

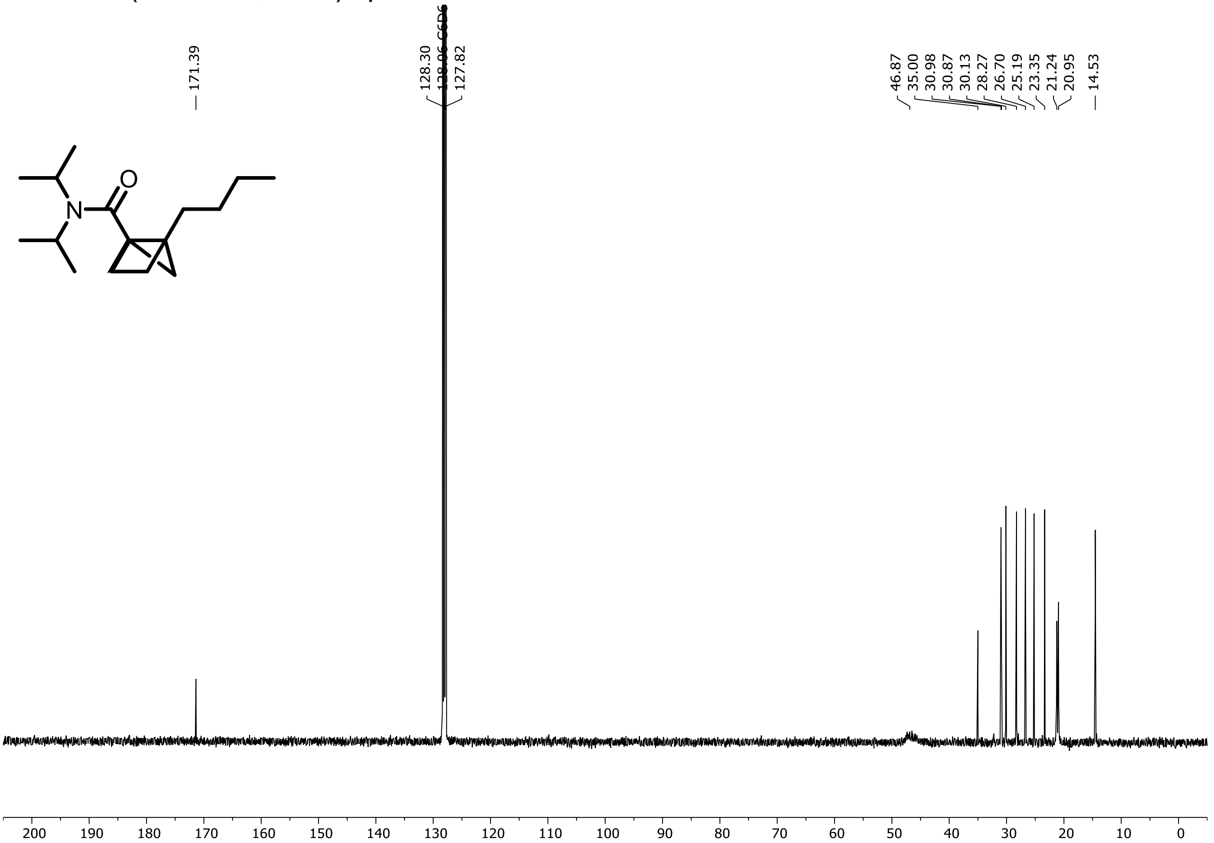

^{1}H NMR (500 MHz, $CDCl_3$) spectrum of **4c**

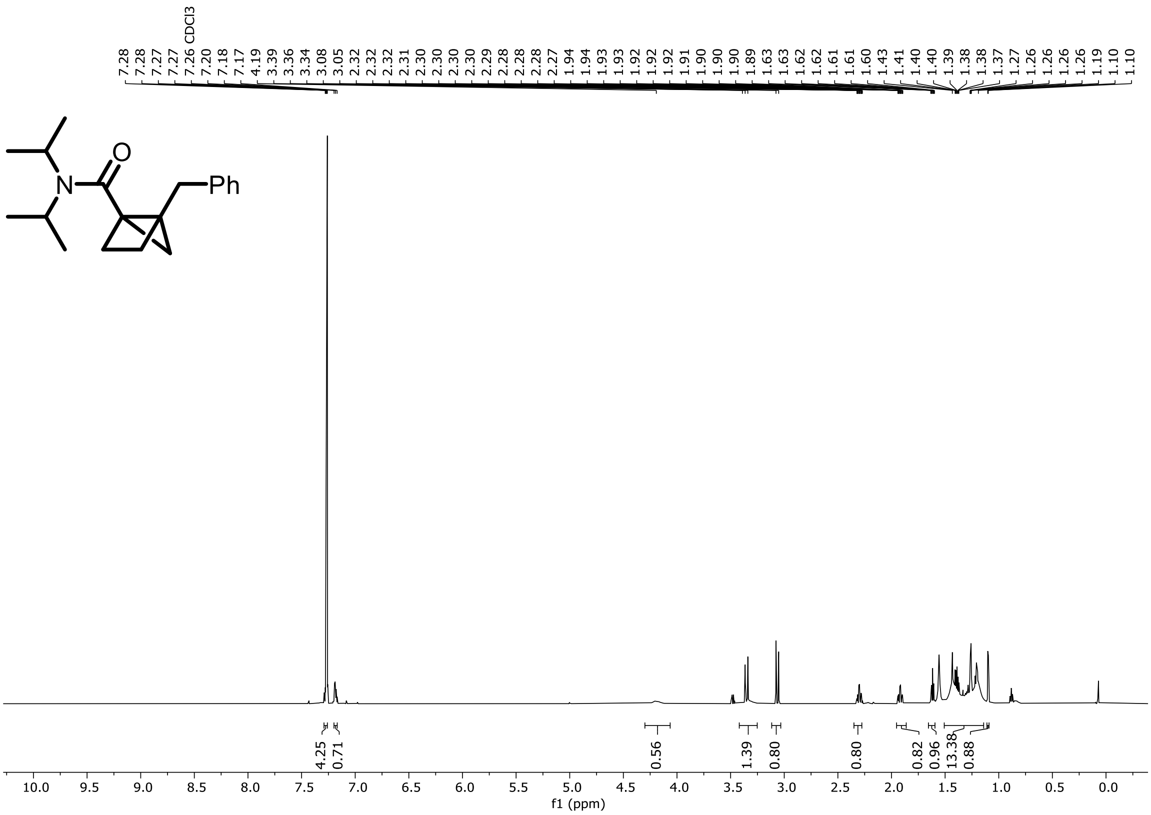

^{13}C NMR (126 MHz, $CDCl_3$) spectrum of **4c**

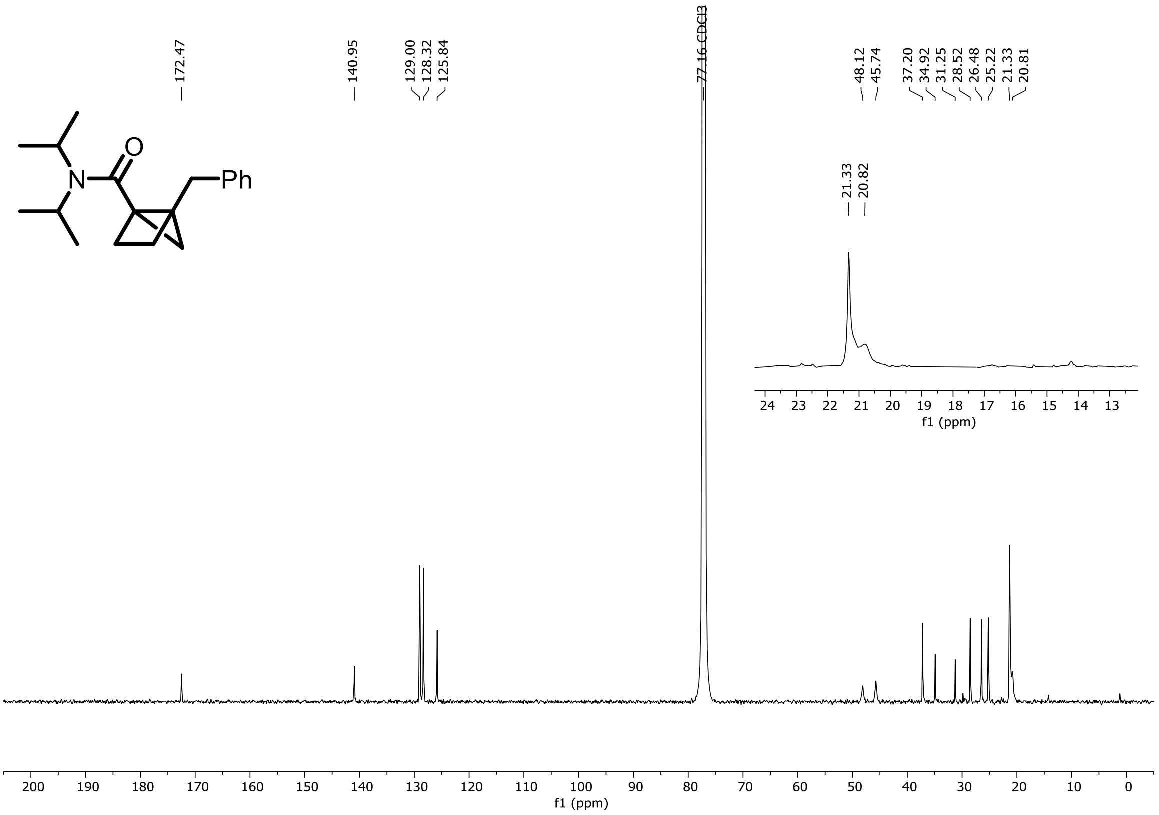

^{1}H NMR (500 MHz, $CDCl_3$) spectrum of **4d**

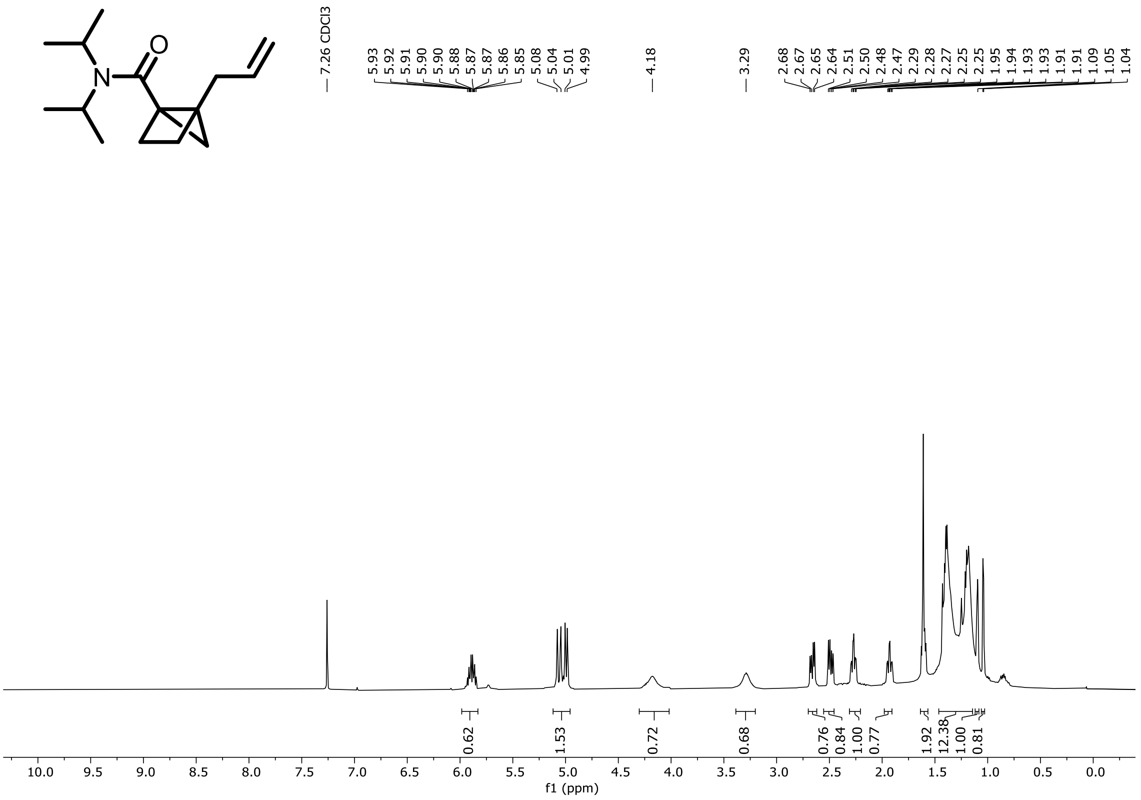

^{13}C NMR (126 MHz, $CDCl_3$) spectrum of **4d**

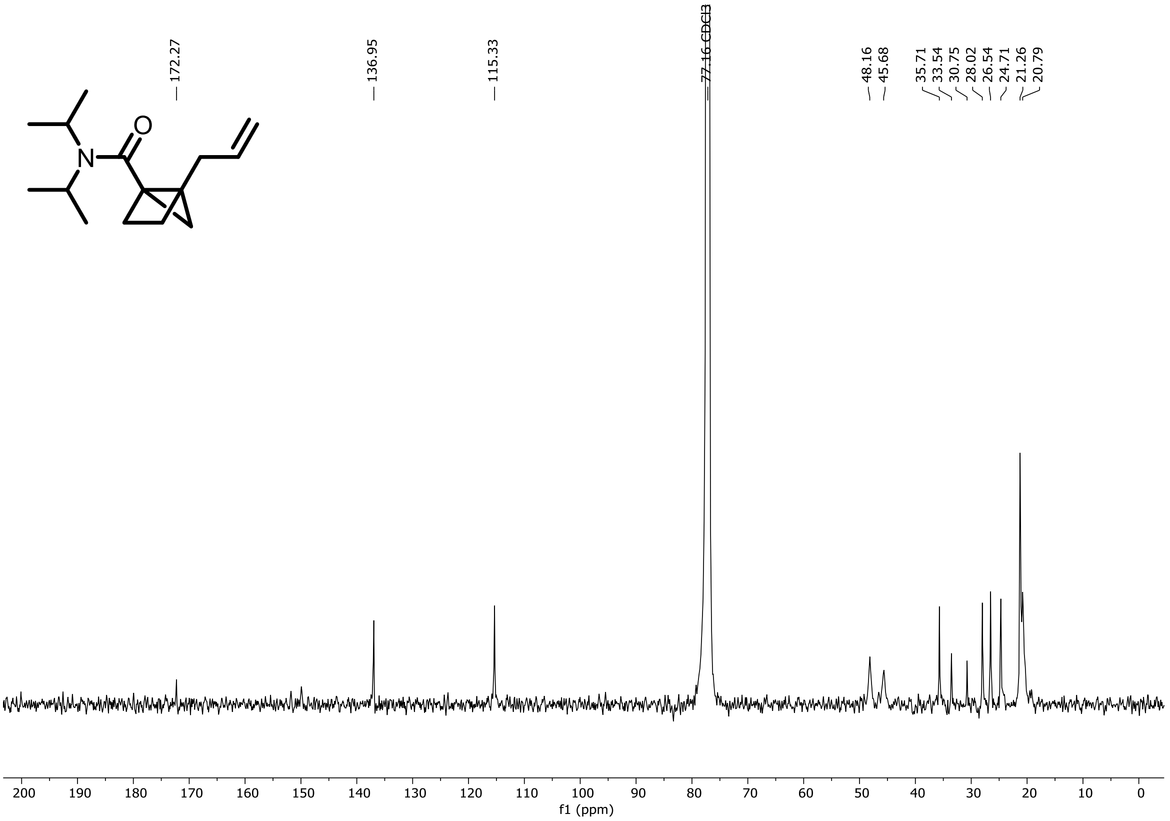

^{1}H NMR (500 MHz, $CDCl_3$) spectrum of **4e**

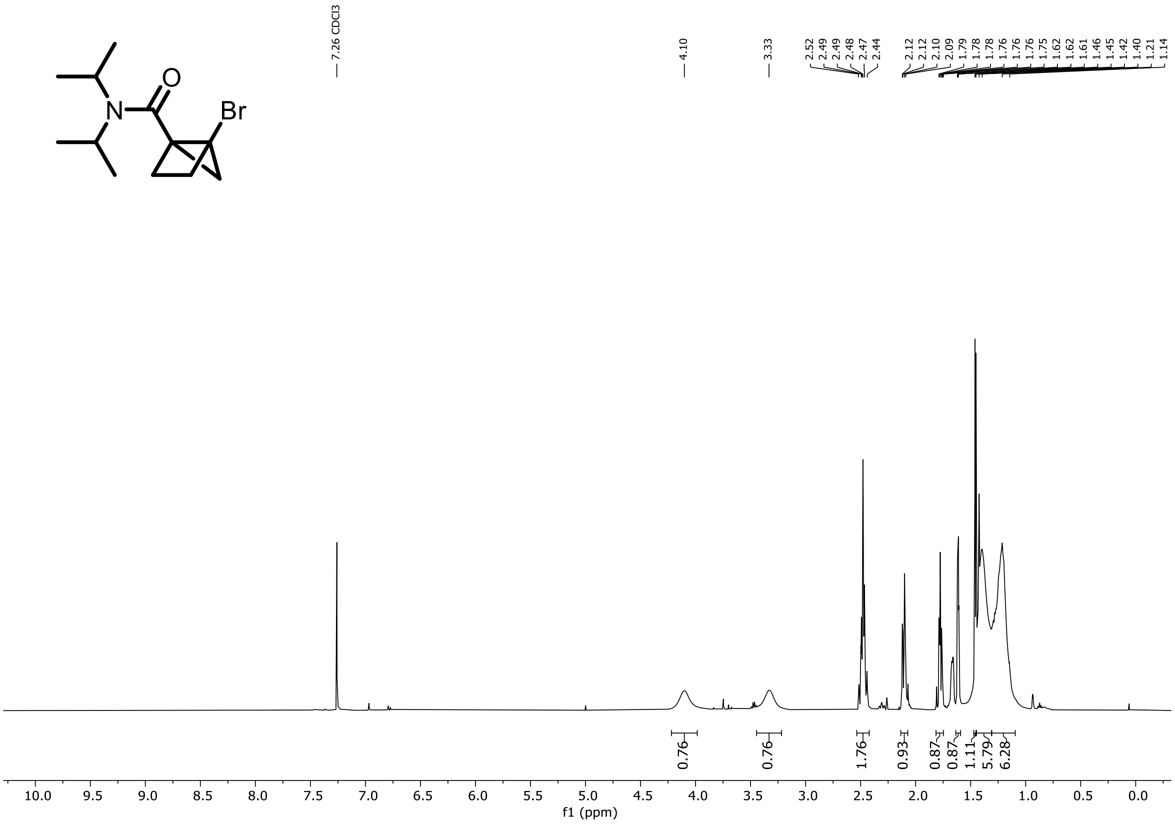

^{13}C NMR (126 MHz, $CDCl_3$) spectrum of **4e**

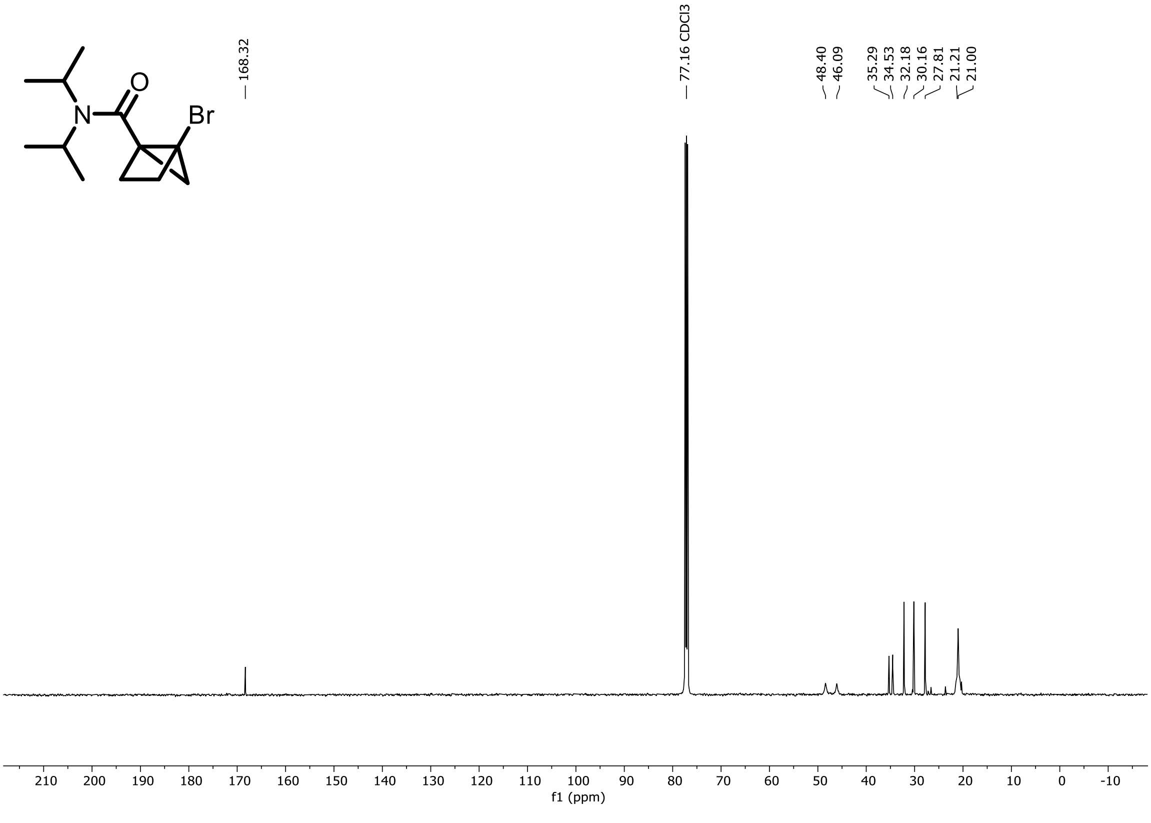

^{1}H NMR (500 MHz, $CDCl_3$) spectrum of **4f**

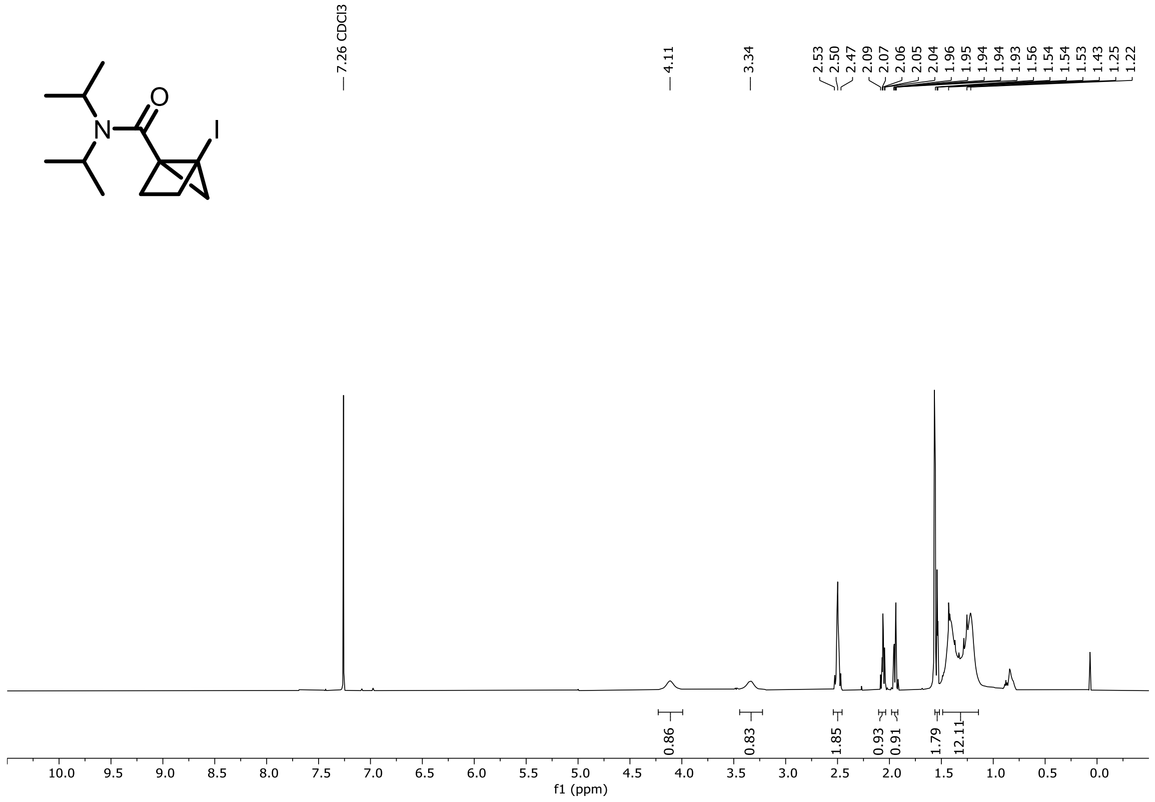

^{13}C NMR (126 MHz, $CDCl_3$) spectrum of **4f**

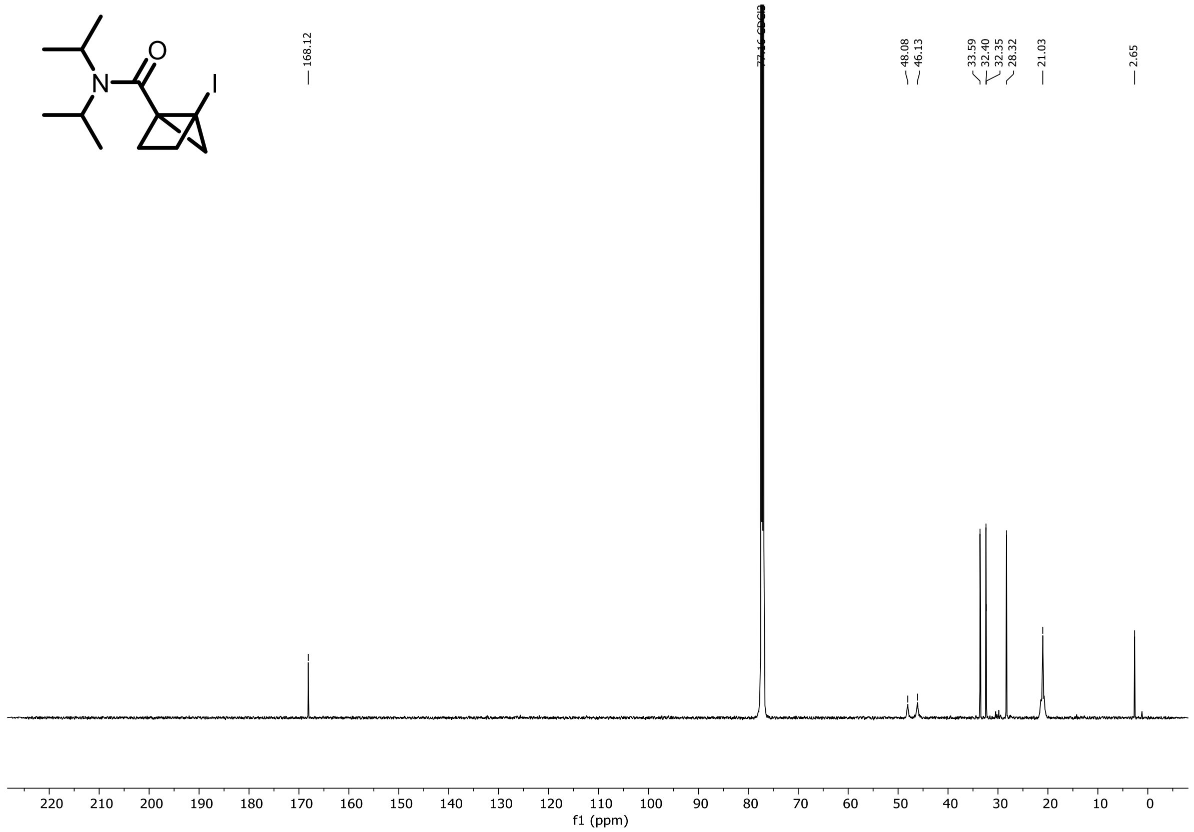

^{1}H NMR (500 MHz, CDCl$_3$) spectrum of **4g**

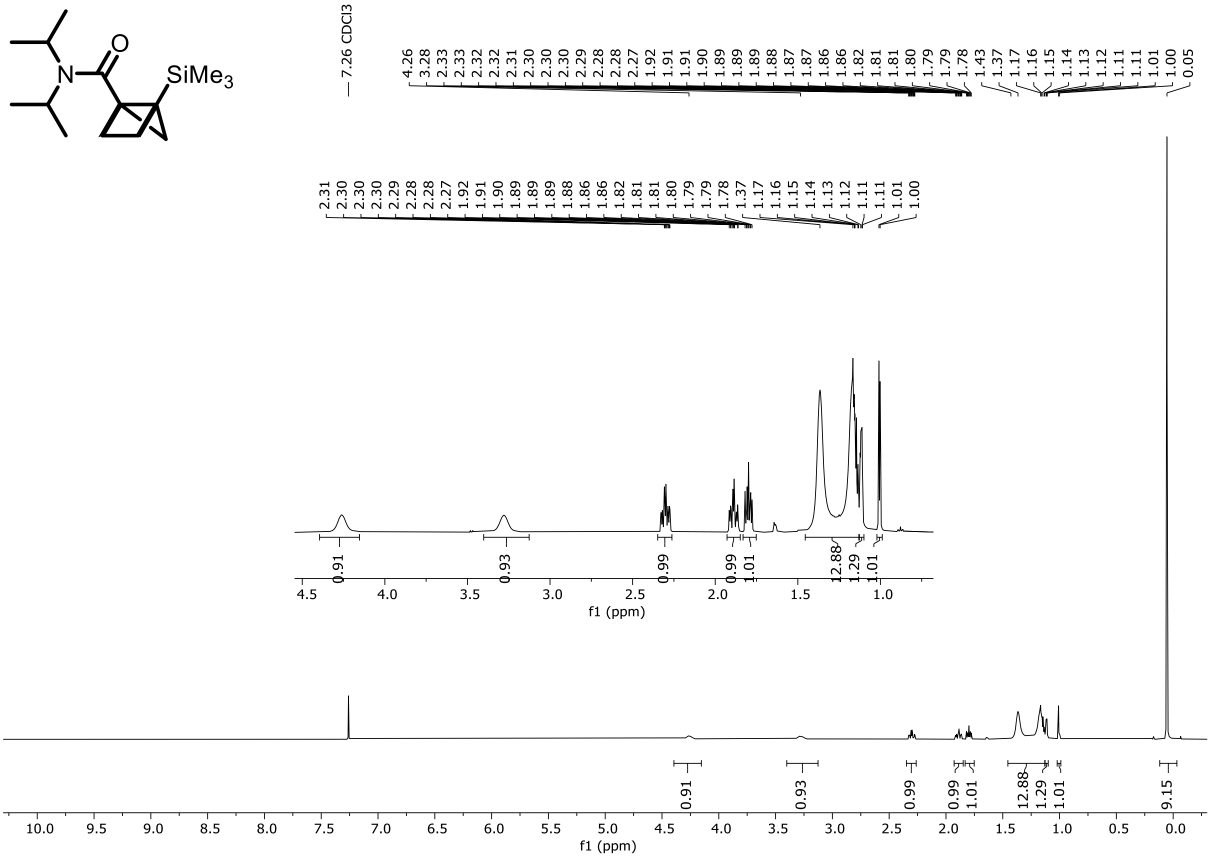

^{13}C NMR (126 MHz, CDCl$_3$) spectrum of **4g**

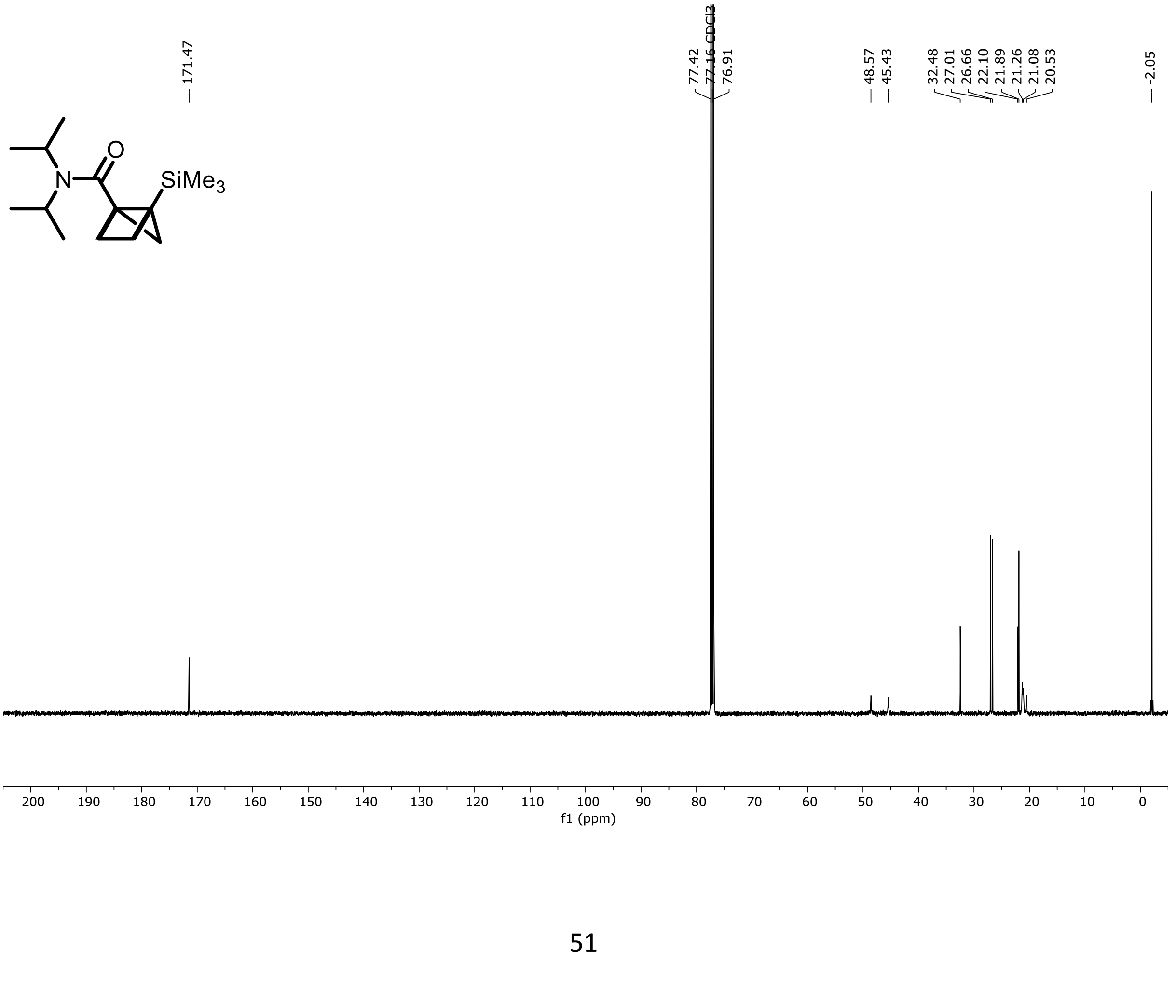

^{1}H NMR (400 MHz, C_6D_6) spectrum of **4h**

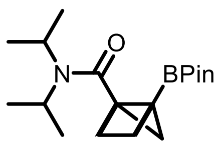

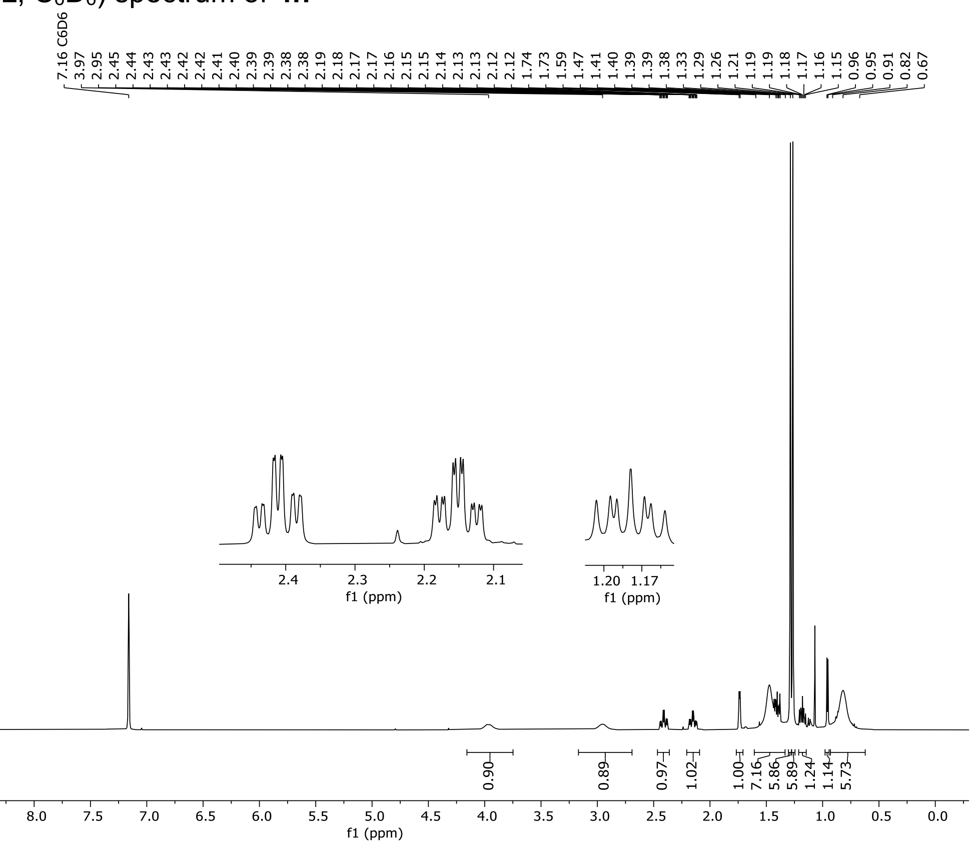

^{13}C NMR (101 MHz, C_6D_6) spectrum of **4h**

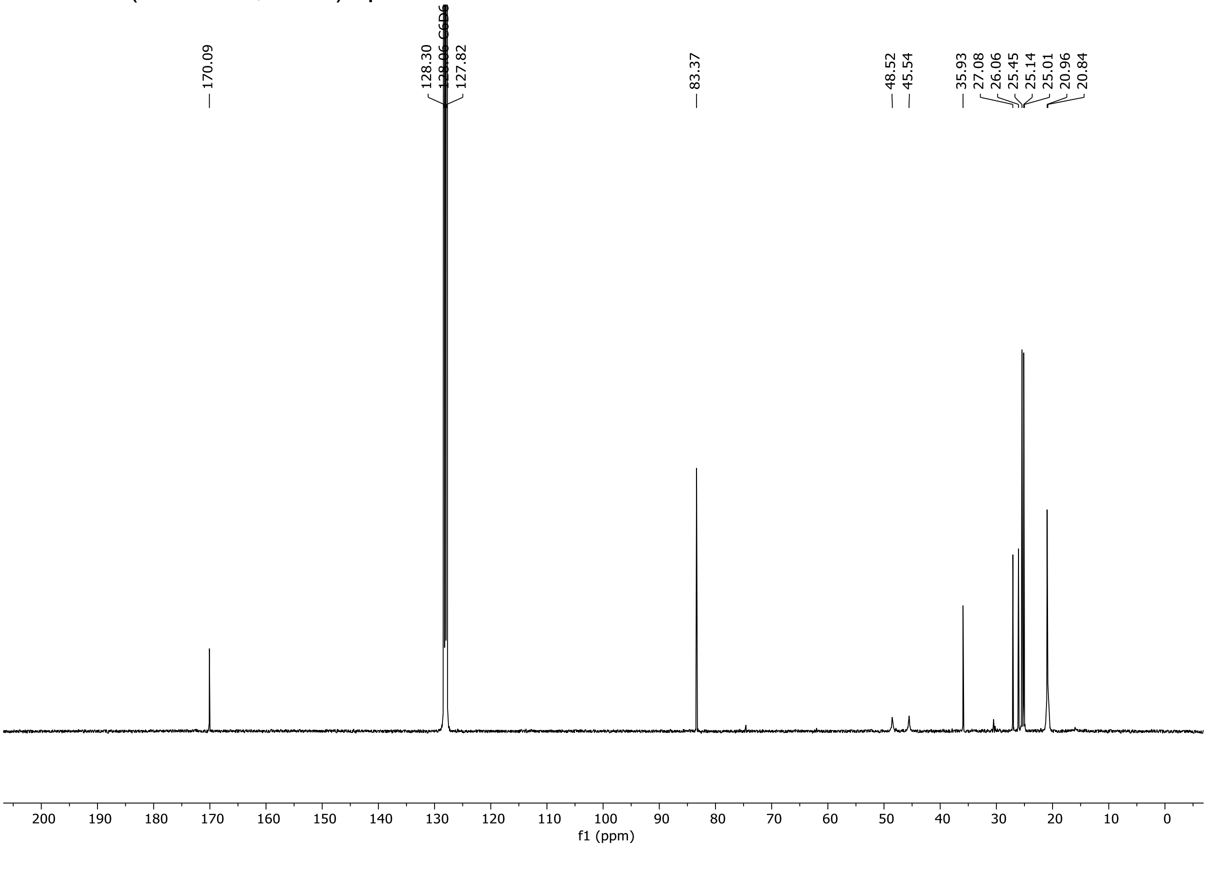

^{11}B NMR (128 MHz, C_6D_6) spectrum of **4h**

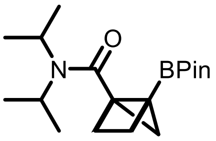

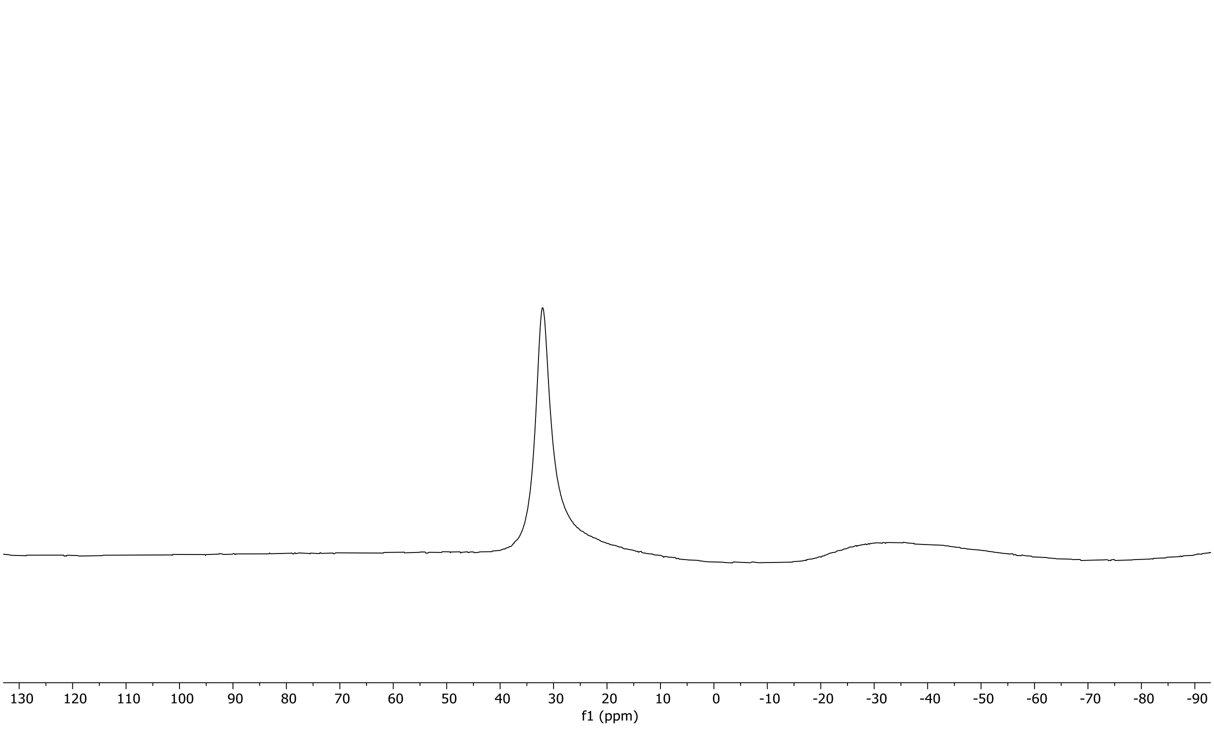

^{1}H NMR (500 MHz, C_6D_6) spectrum of **4i**

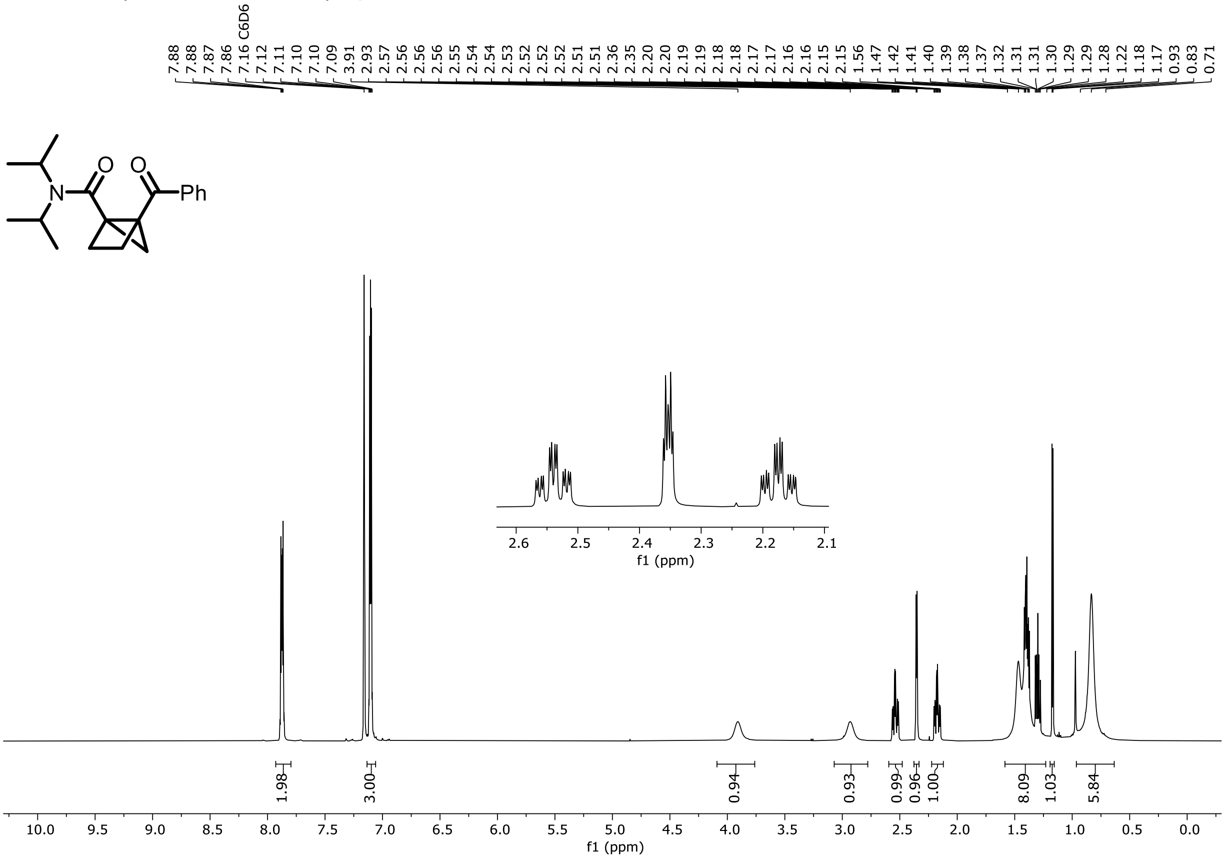

^{13}C NMR (126 MHz, C_6D_6) spectrum of **4i**

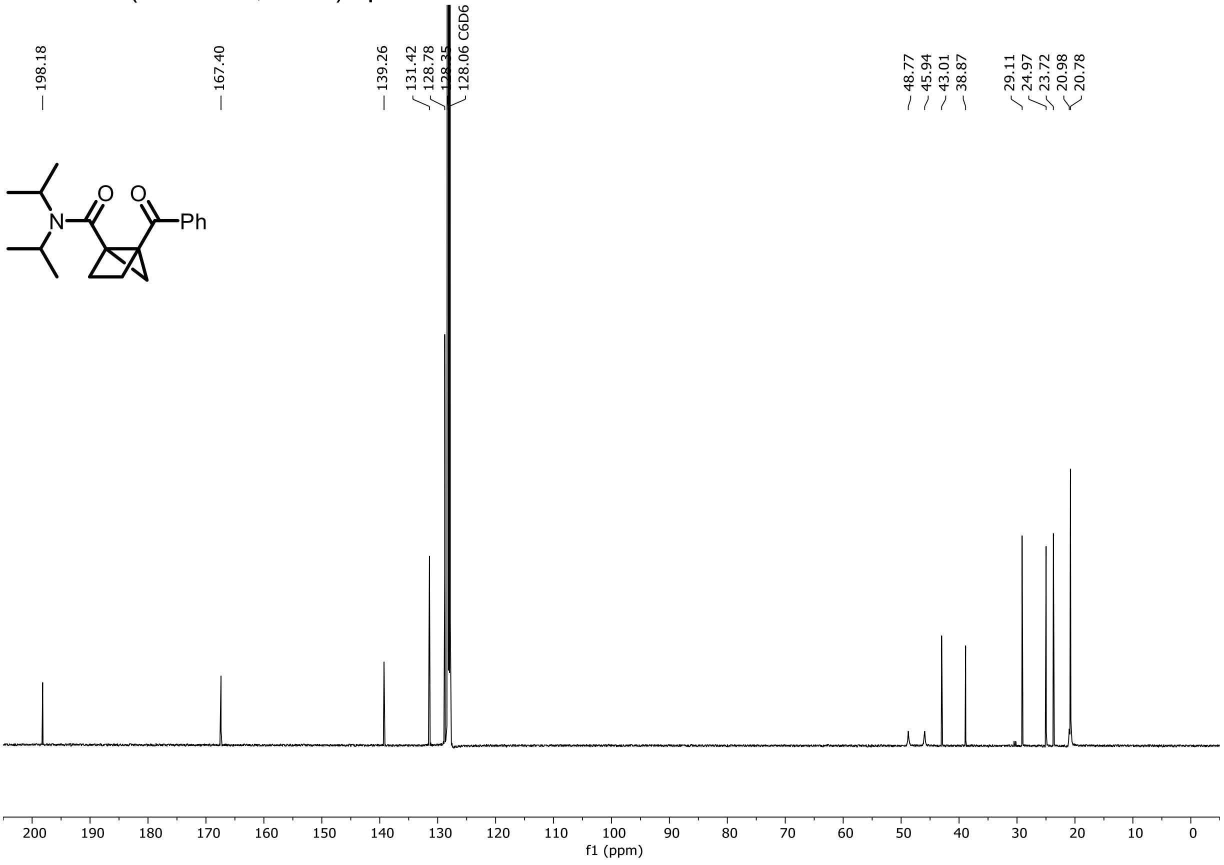

^{1}H NMR (500 MHz, $CDCl_3$) spectrum of **4j** (major isomer)

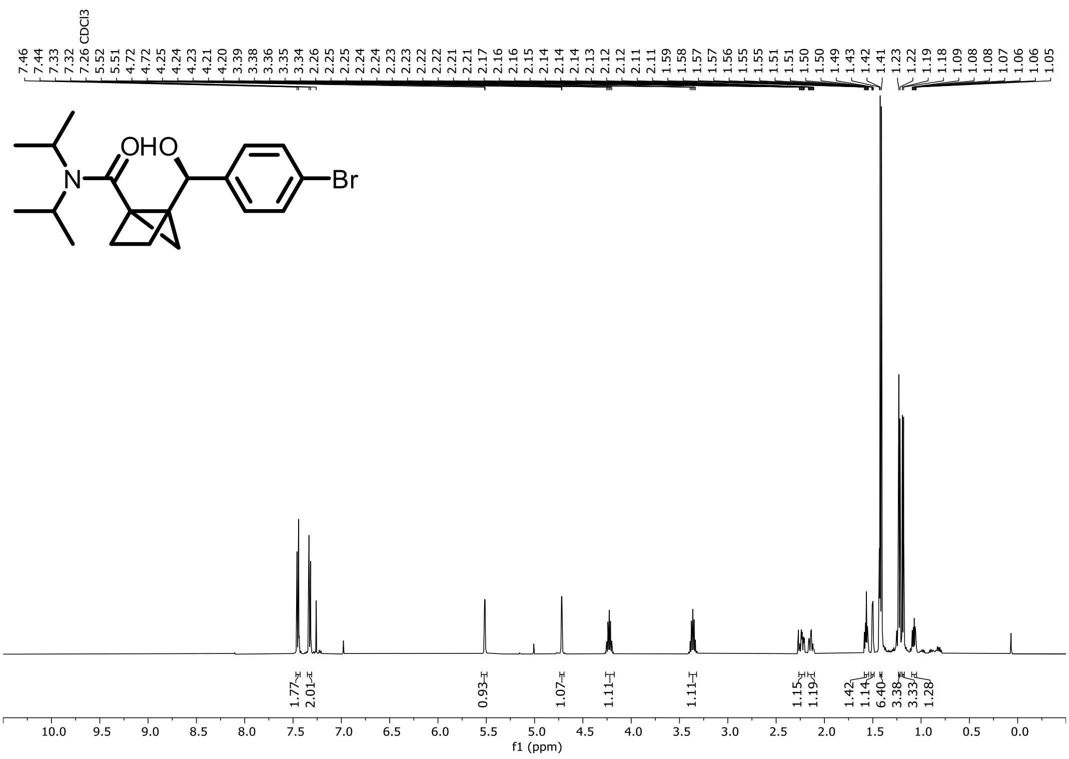

^{13}C NMR (126 MHz, $CDCl_3$) spectrum of **4j** (major isomer)

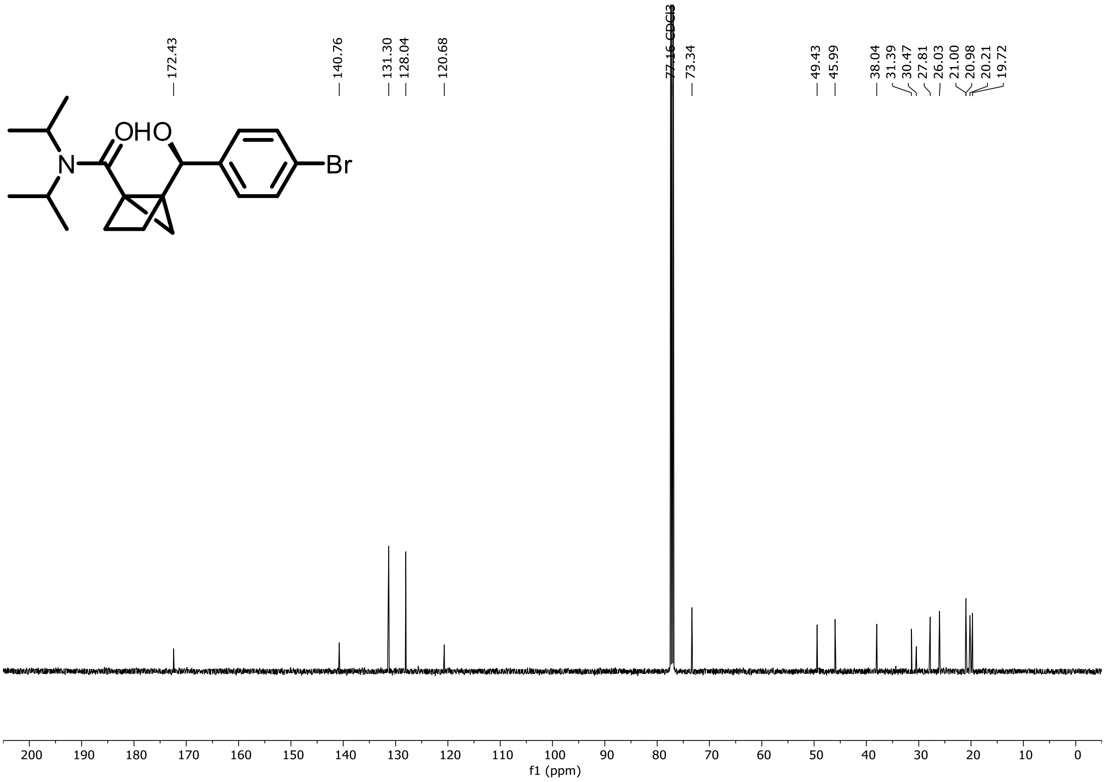

^{1}H NMR (500 MHz, $CDCl_3$) spectrum of **4j'** (minor isomer)

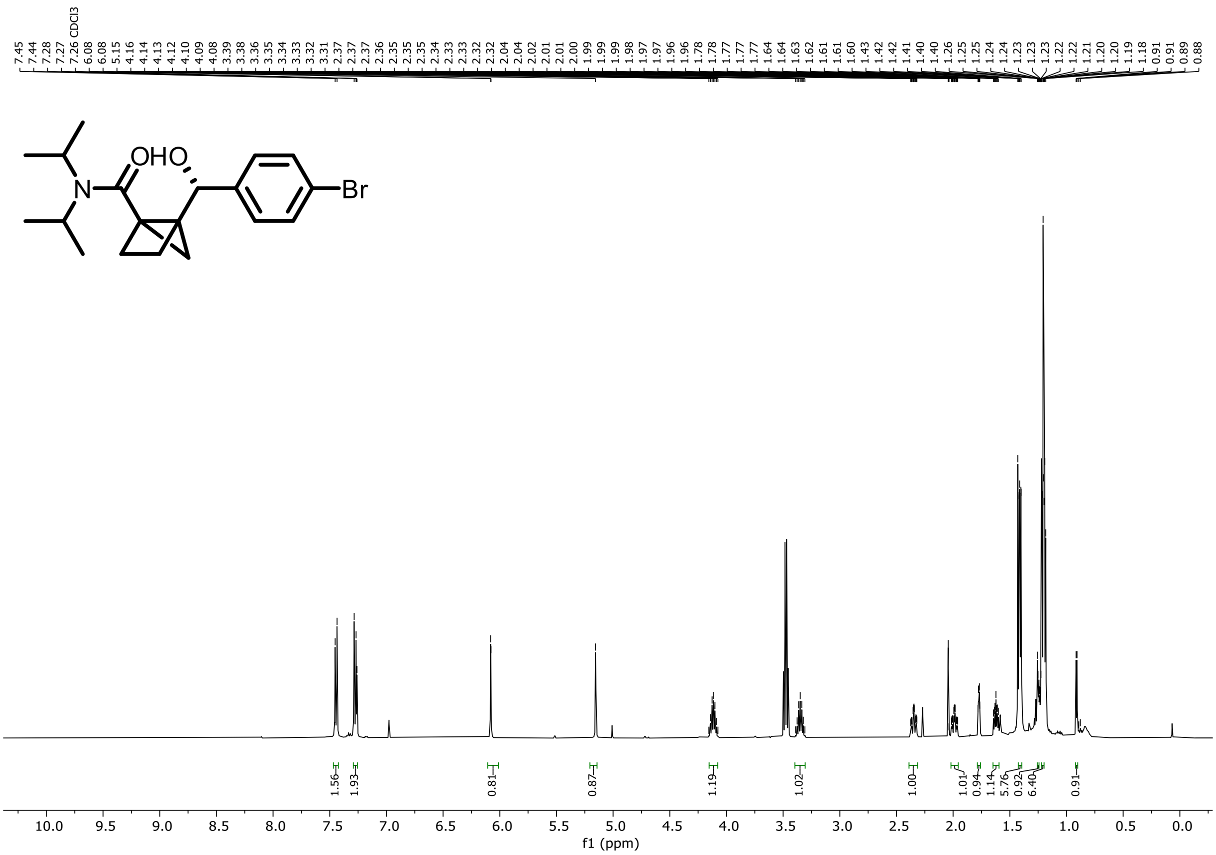

^{13}C NMR (126 MHz, $CDCl_3$) spectrum of **4j'** (minor isomer)

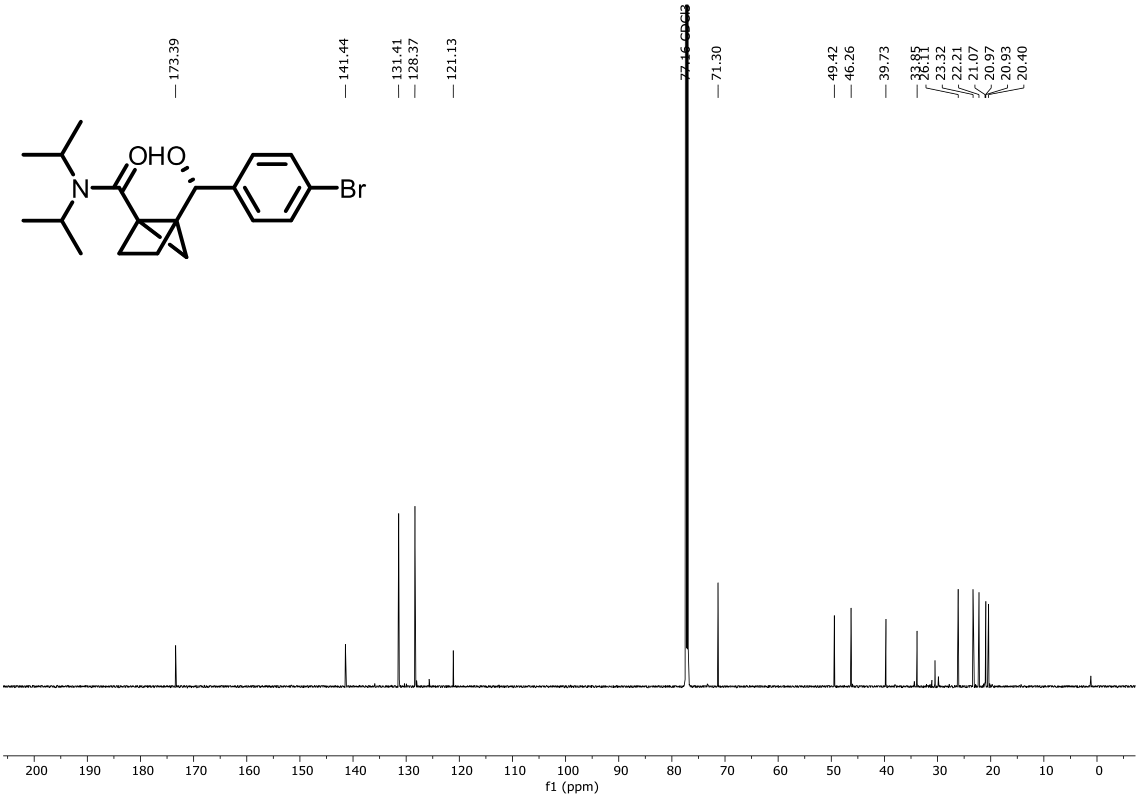

^{1}H NMR (500 MHz, $CDCl_3$) spectrum of **4k**

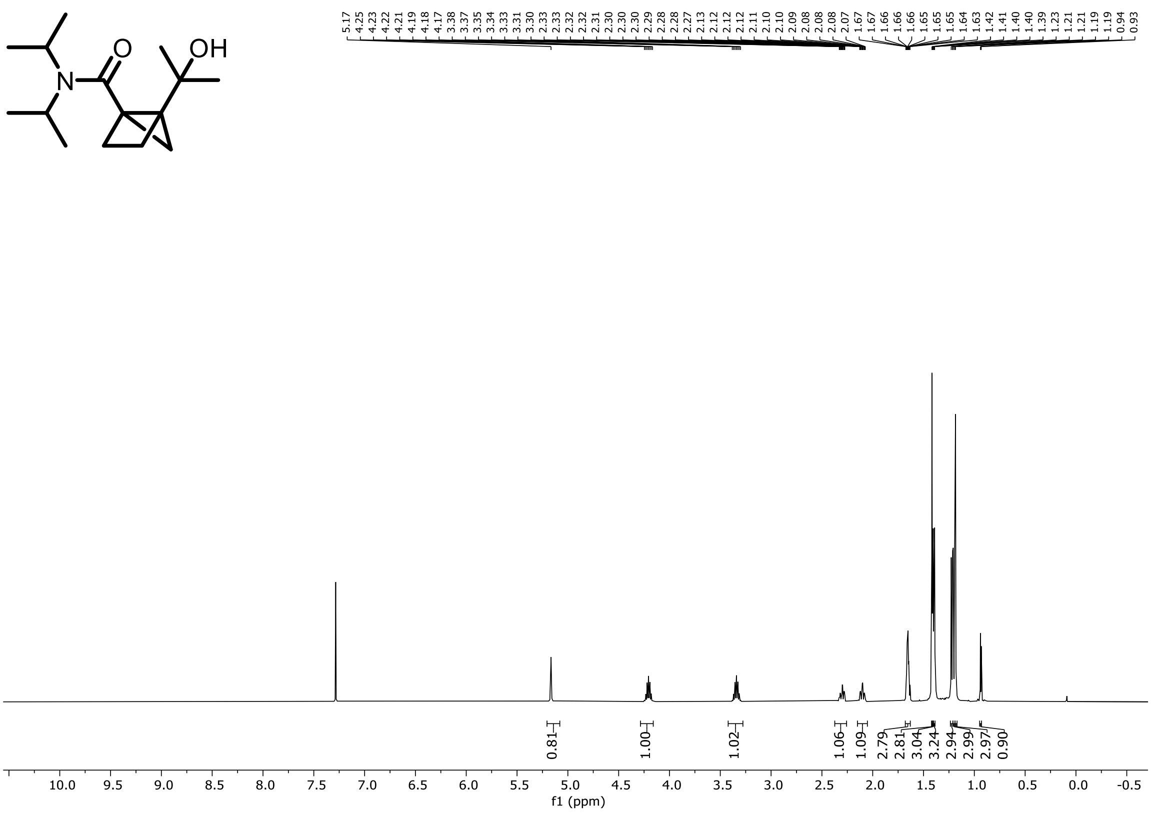

^{13}C NMR (126 MHz, $CDCl_3$) spectrum of **4k**

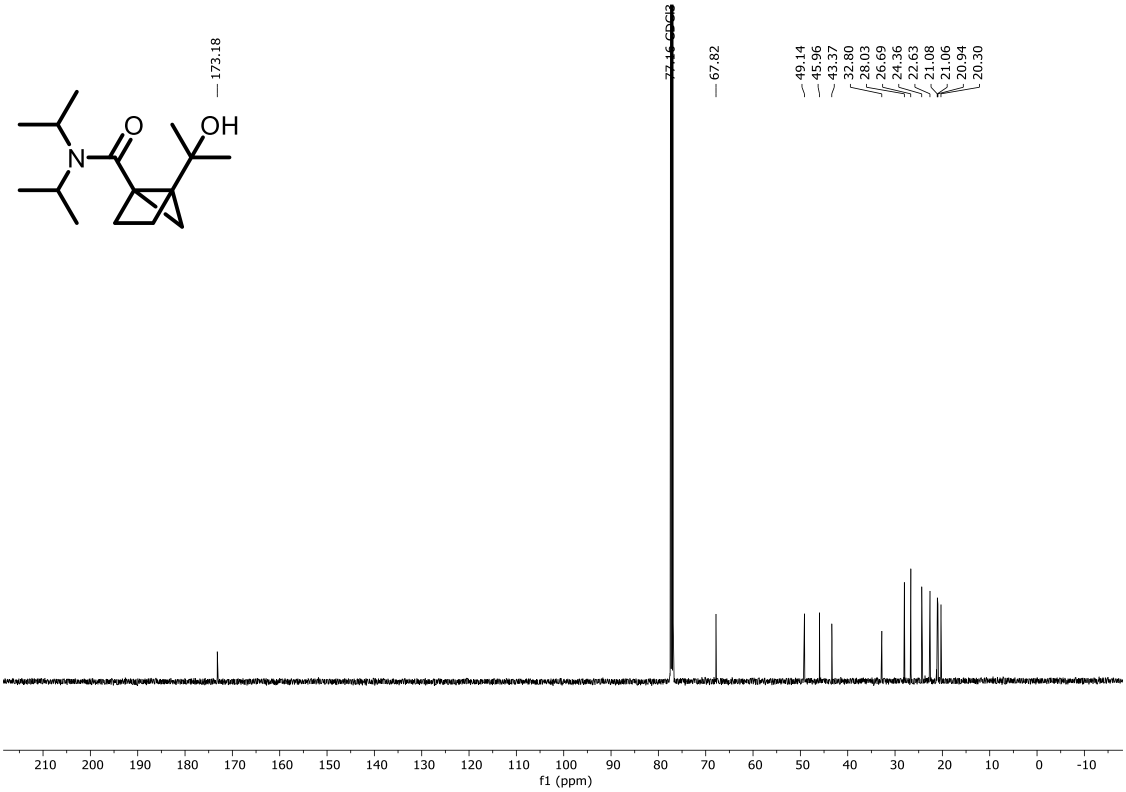

^{1}H NMR (500 MHz, C_6D_6) spectrum of **4l**

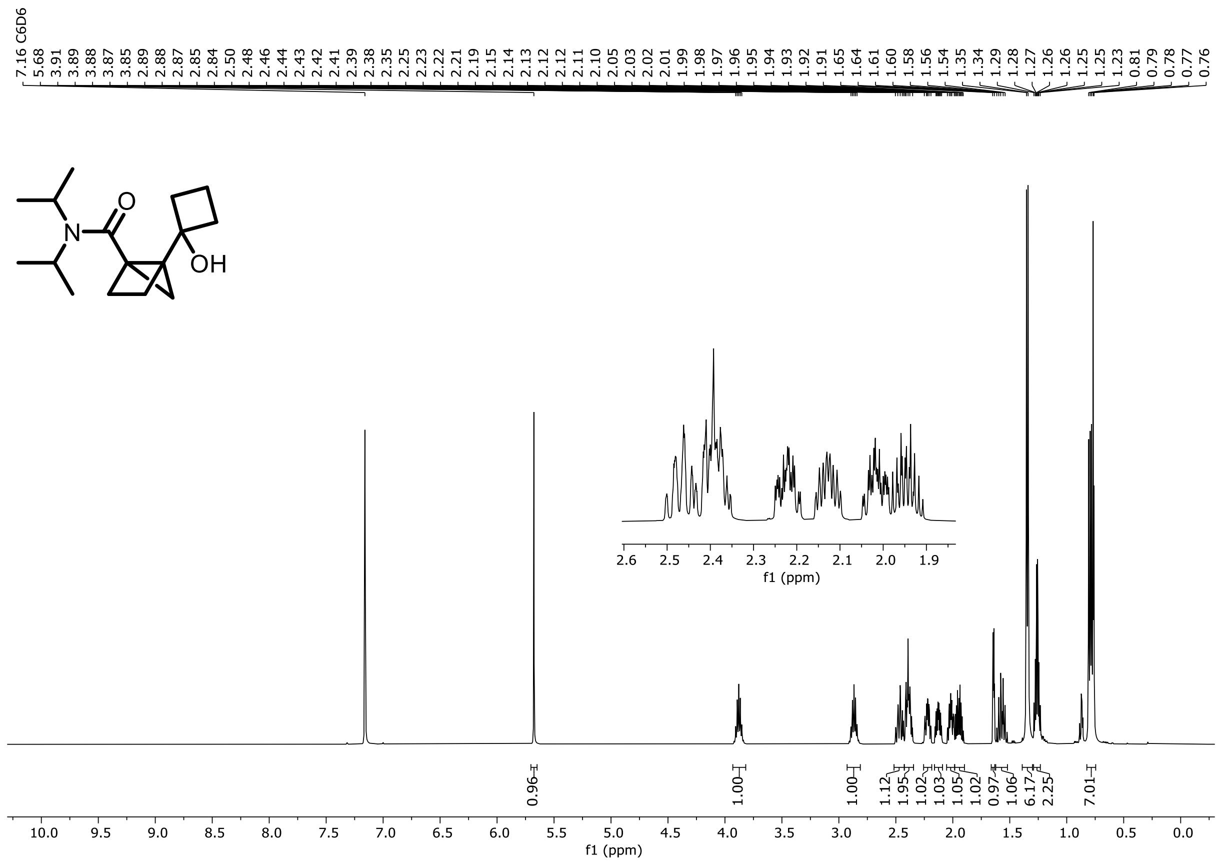

^{13}C NMR (126 MHz, C_6D_6) spectrum of **4l**

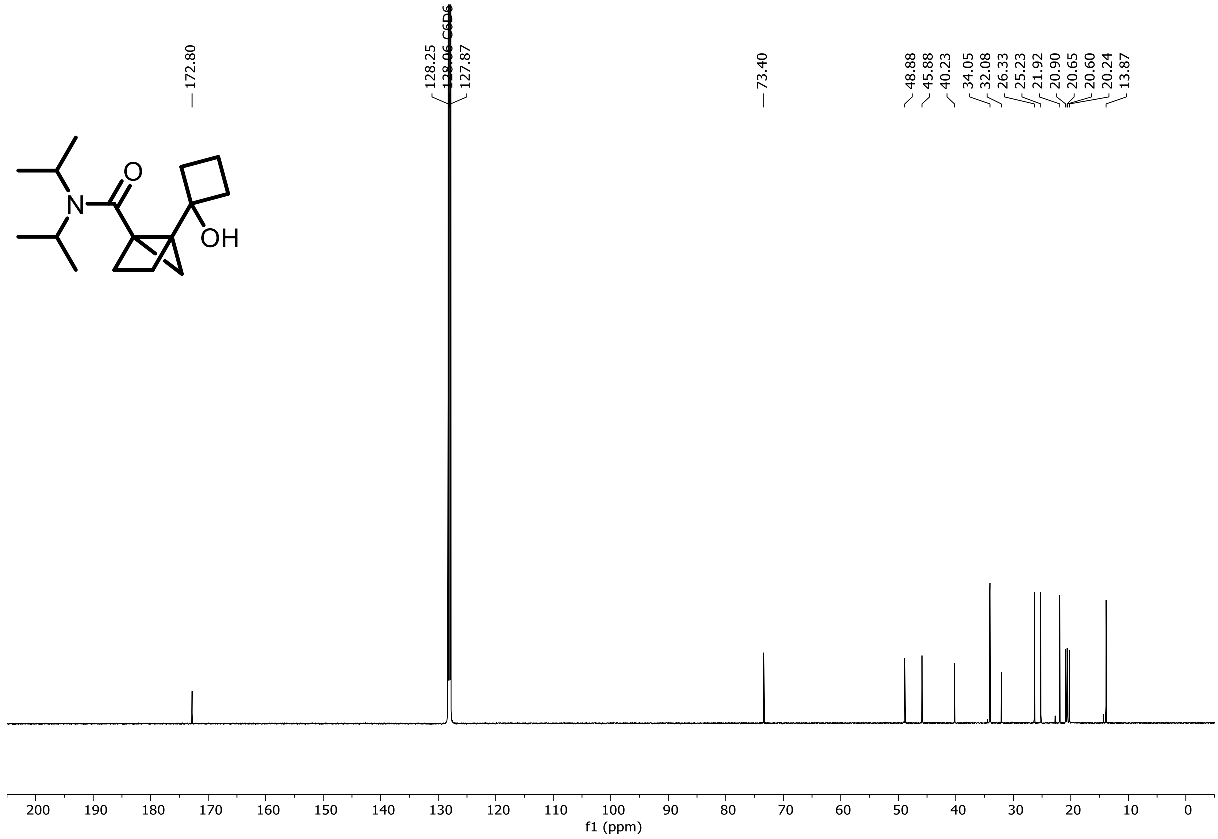

^{1}H NMR (500 MHz, C_6D_6) spectrum of **4m**

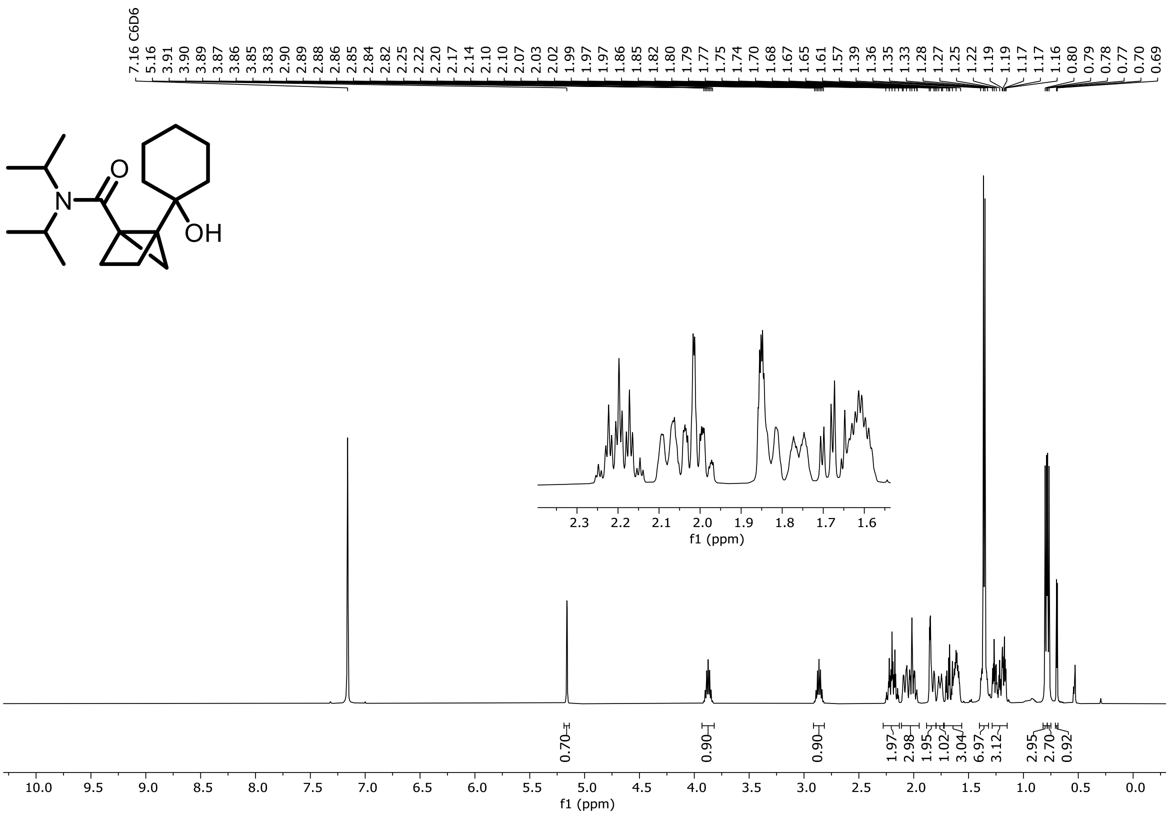

^{13}C NMR (126 MHz, C_6D_6) spectrum of **4m**

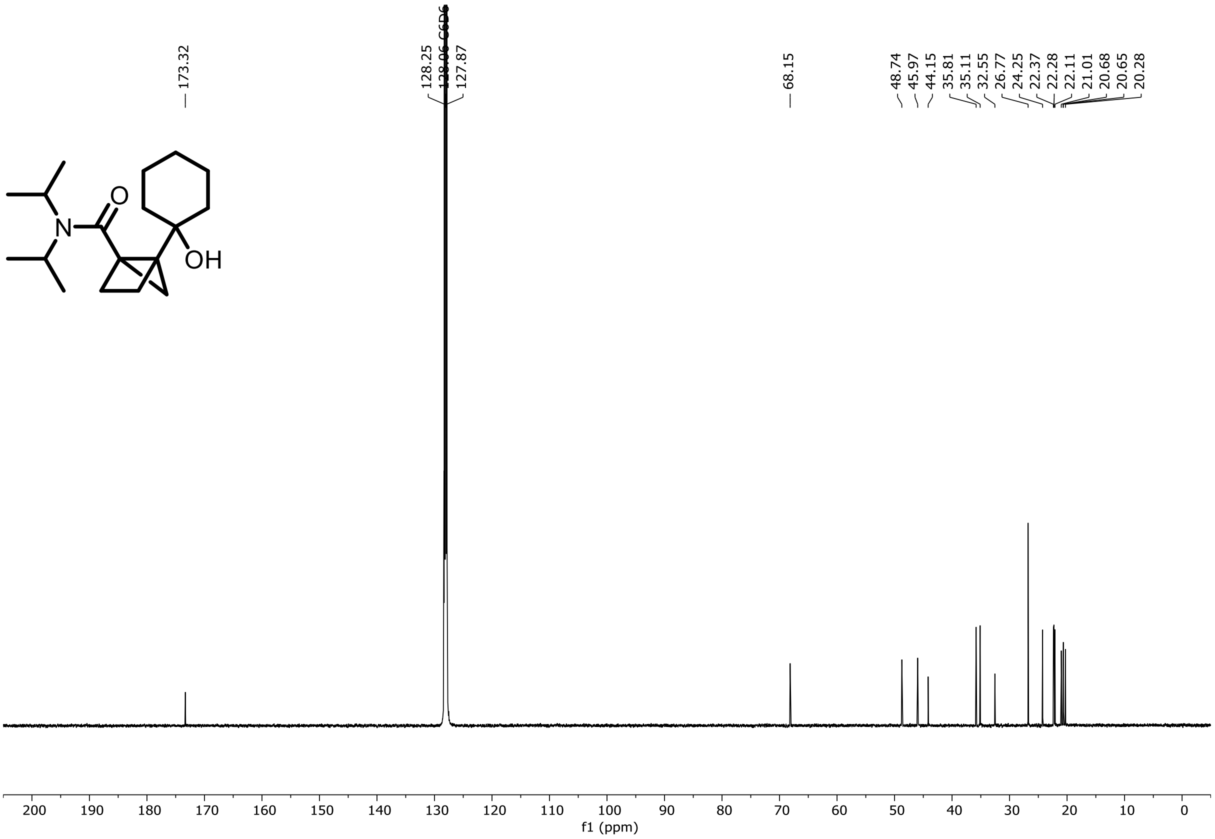

^{1}H NMR (500 MHz, $CDCl_3$) spectrum of **4n**

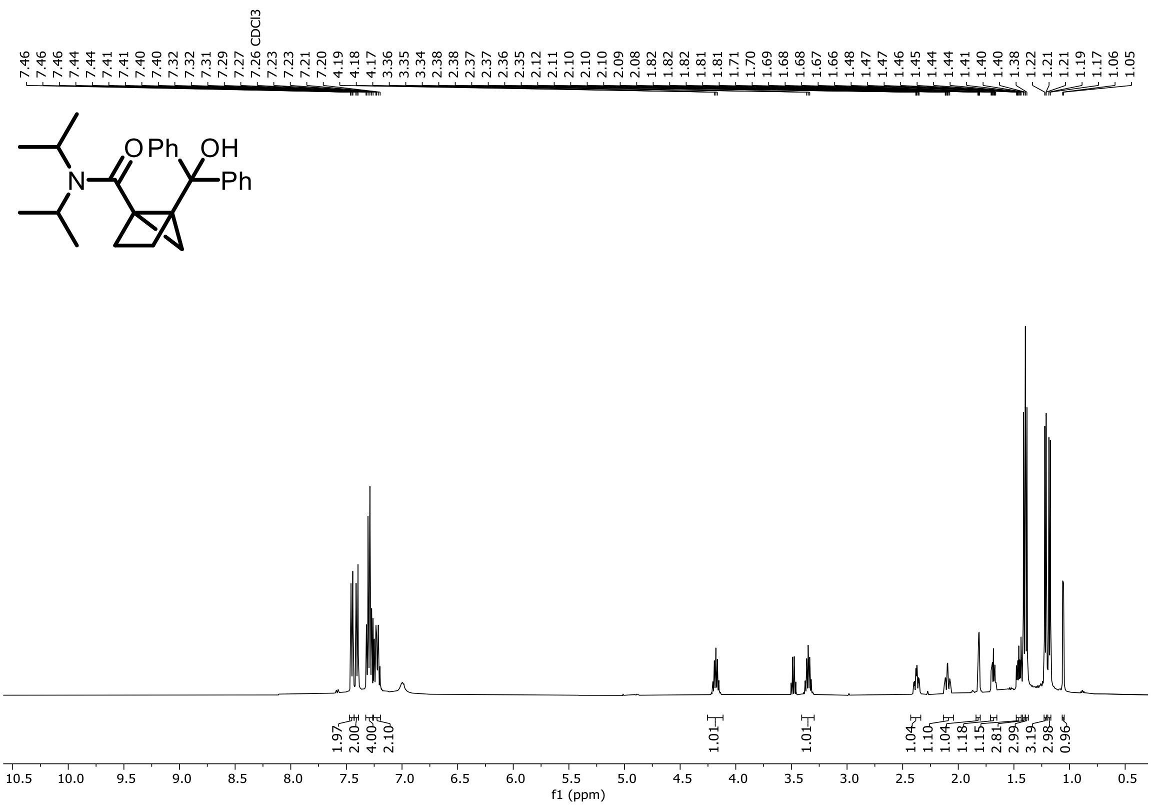

^{13}C NMR (126 MHz, $CDCl_3$) spectrum of **4n**

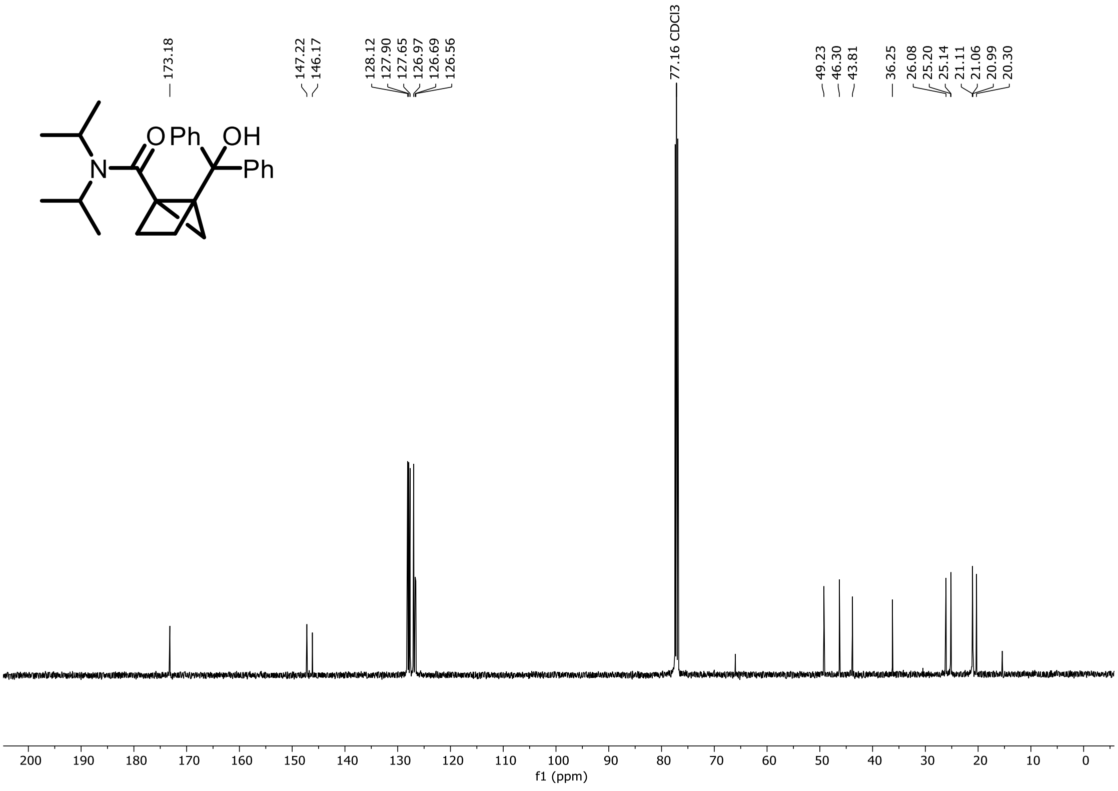

^{1}H NMR (500 MHz, $CDCl_3$) spectrum of **5a**

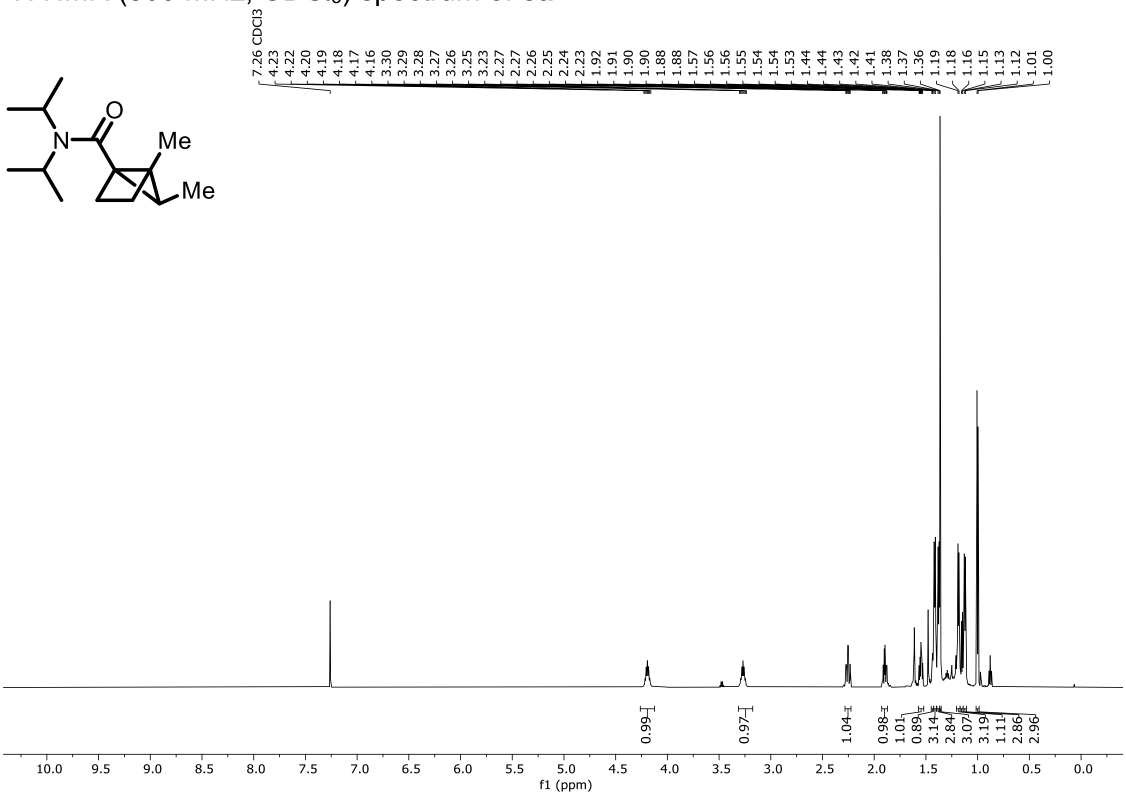

^{13}C NMR (126 MHz, $CDCl_3$) spectrum of **5a**

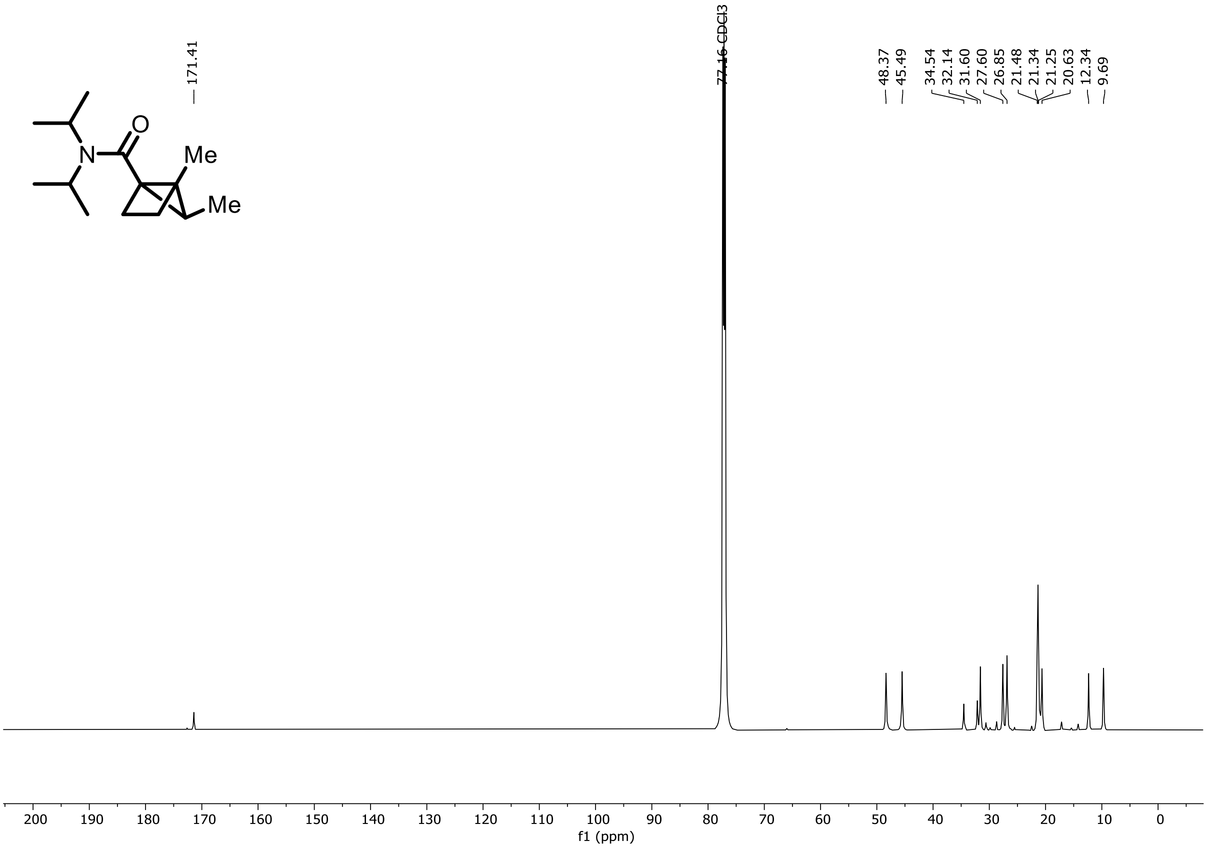

^{1}H NMR (500 MHz, C_6D_6, 298K) spectrum of **5b**

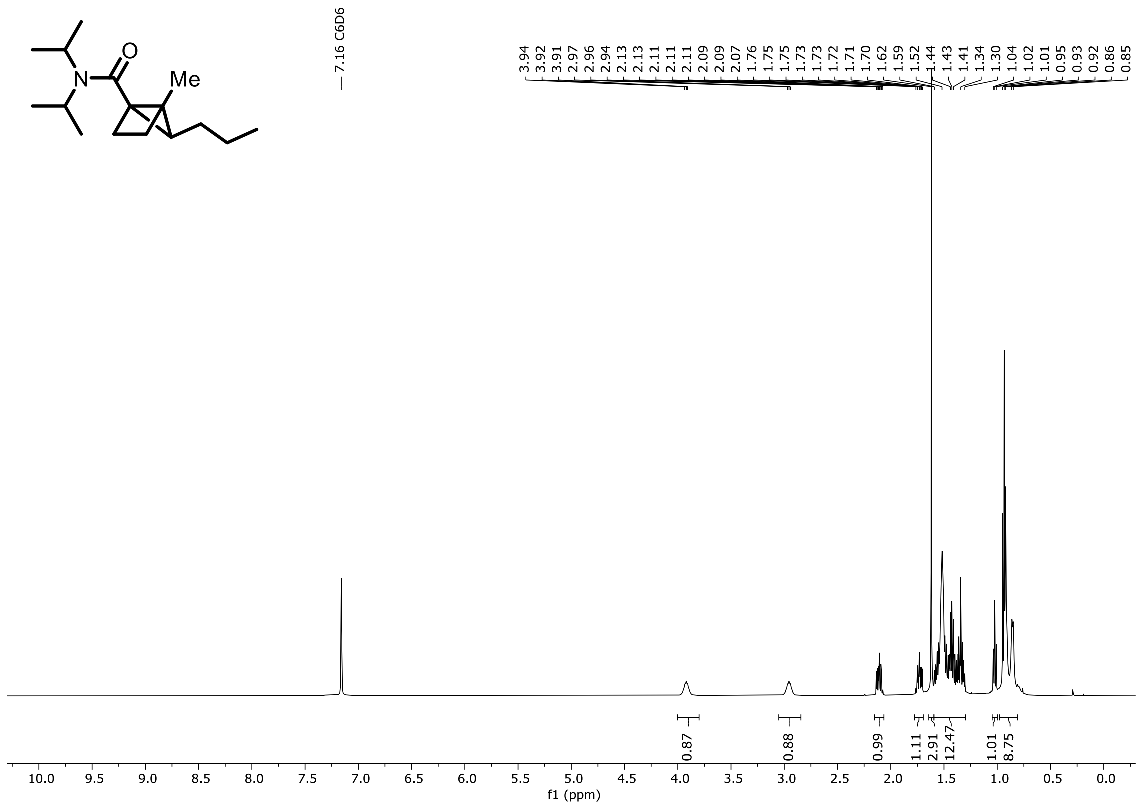

^{13}C NMR (126 MHz, C_6D_6) spectrum of **5b**

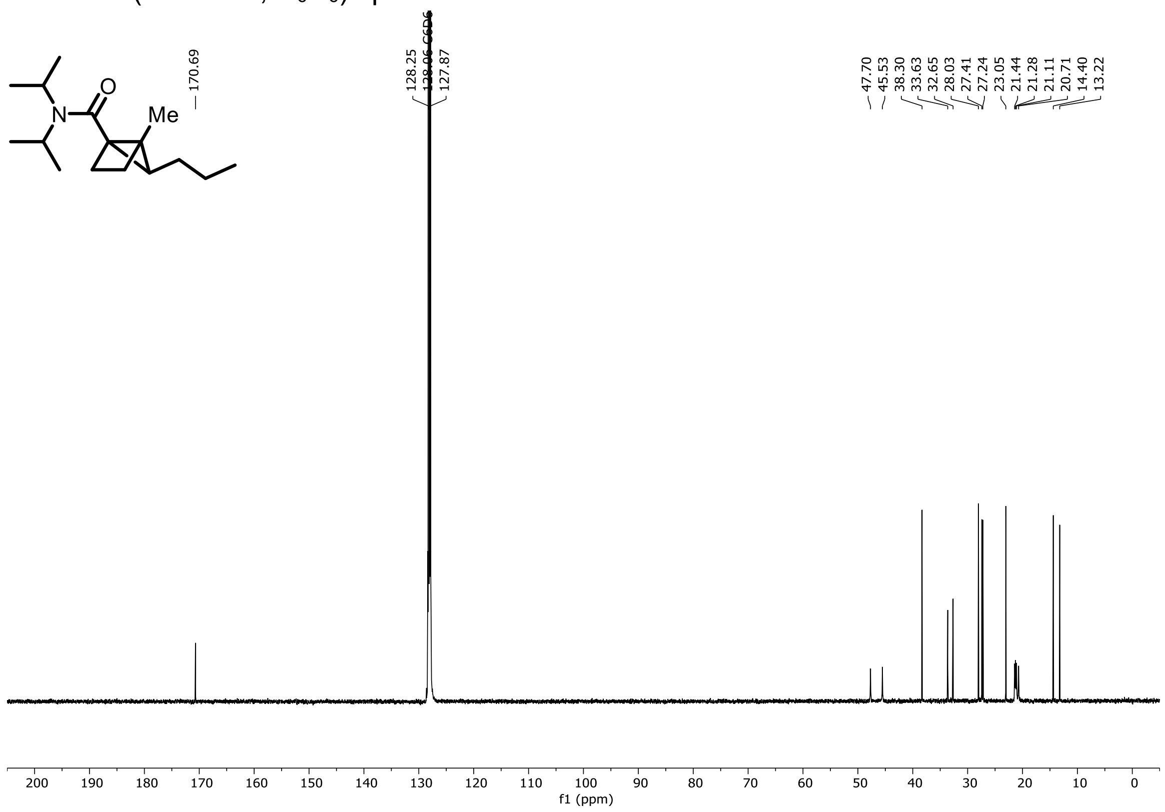

^{1}H NMR (500 MHz, C_6D_6) spectrum of **5c**

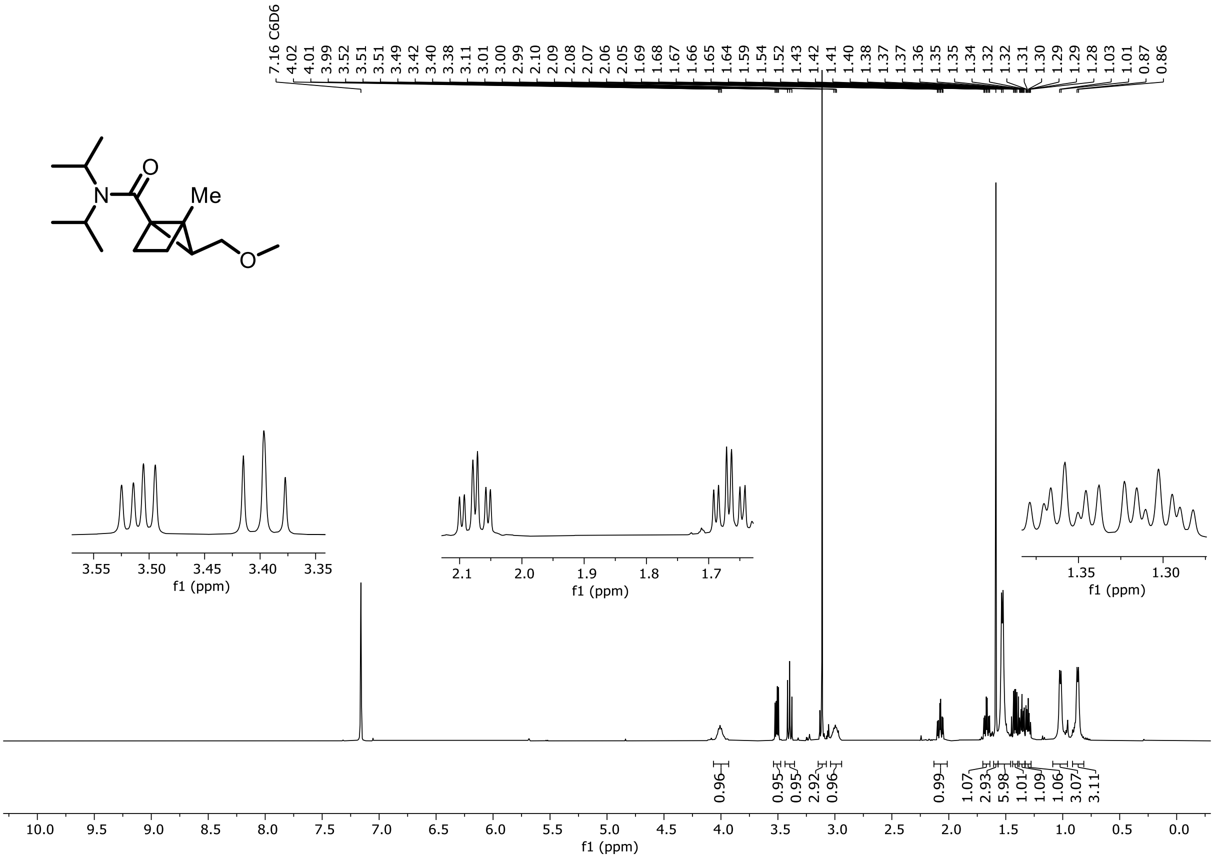

^{13}C NMR (126 MHz, C_6D_6) spectrum of **5c**

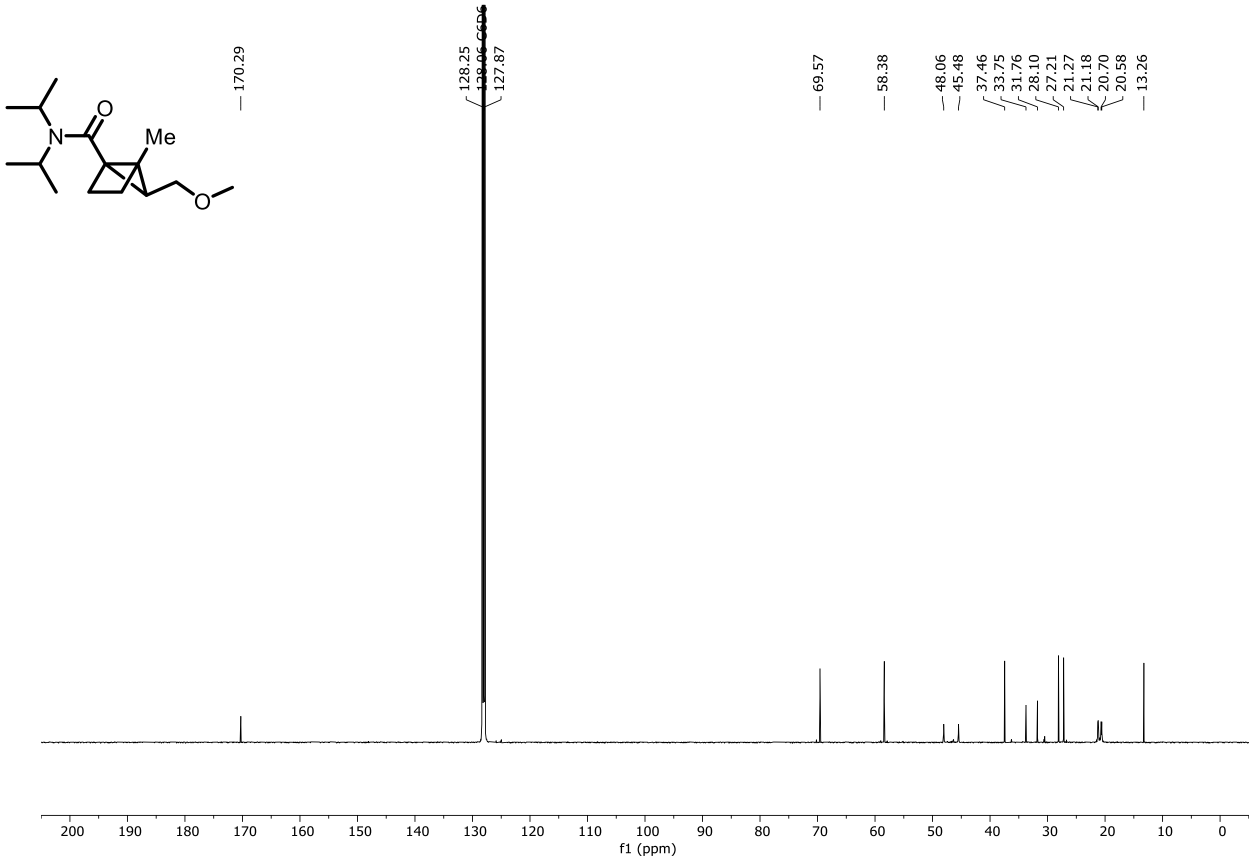

^{1}H NMR (500 MHz, C_6D_6) spectrum of **5d**

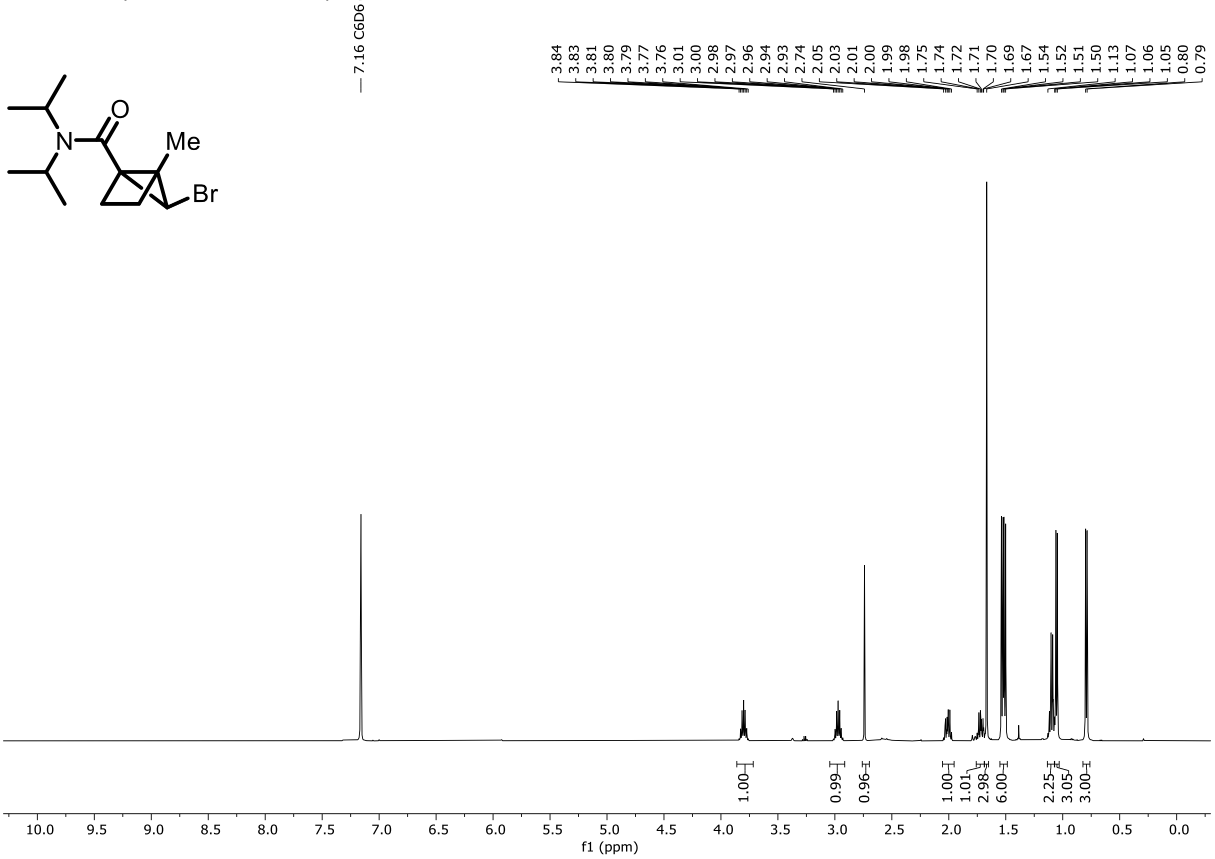

^{13}C NMR (126 MHz, C_6D_6) spectrum of **5d**

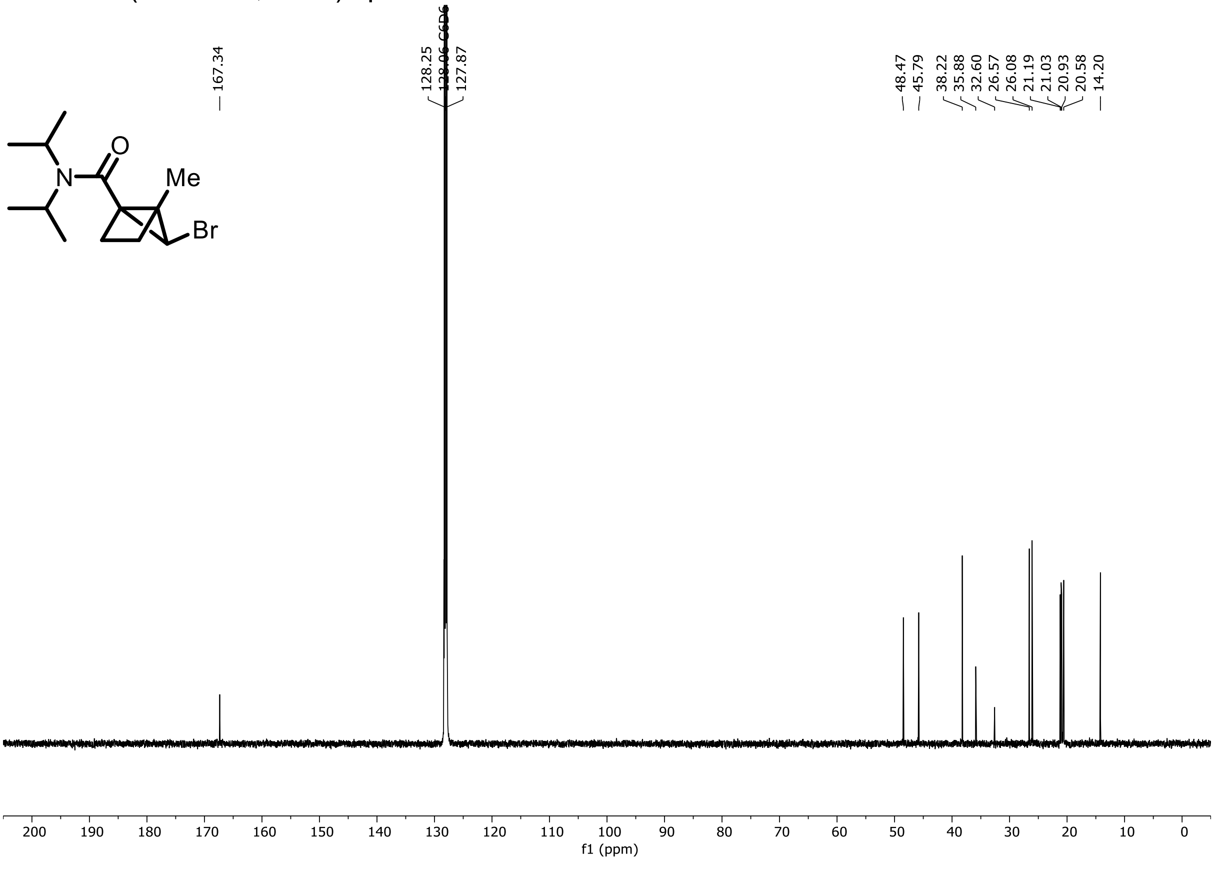

^{1}H NMR (500 MHz, $CDCl_3$) spectrum of **5e**

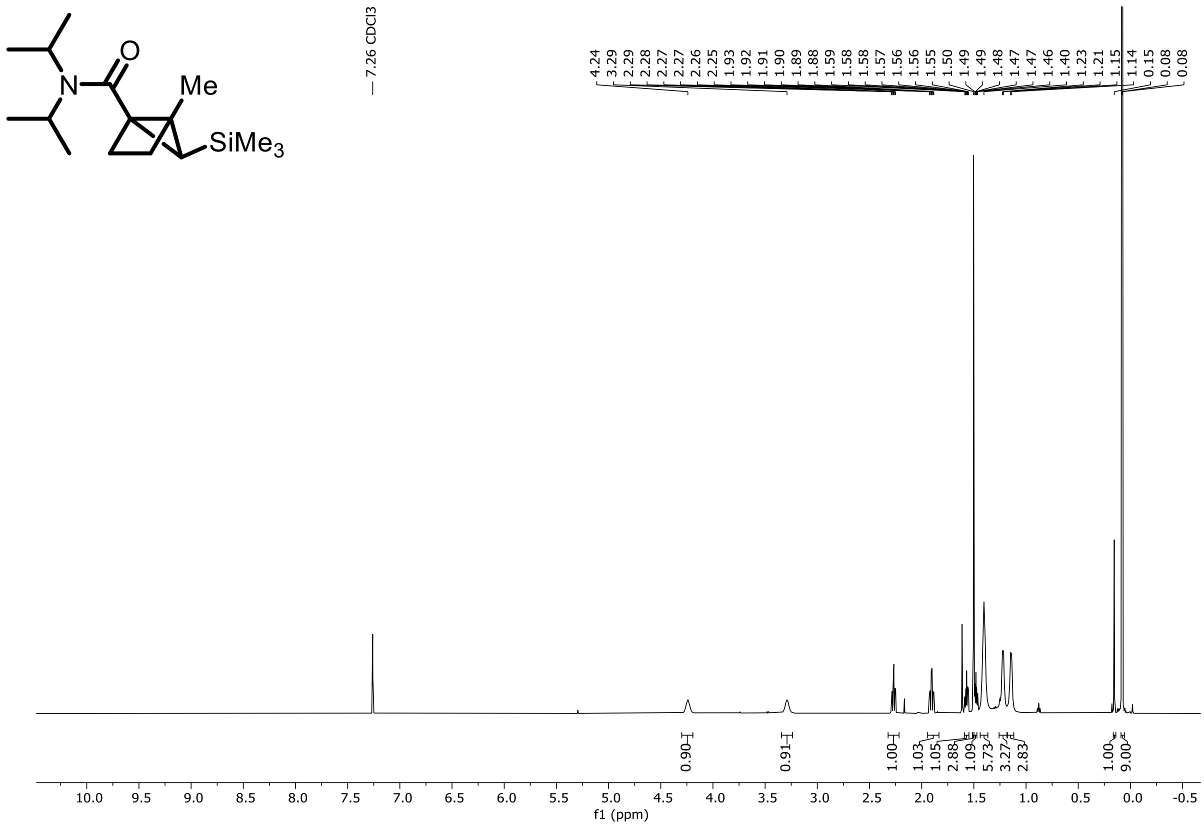

^{13}C NMR (126 MHz, $CDCl_3$) spectrum of **5e**

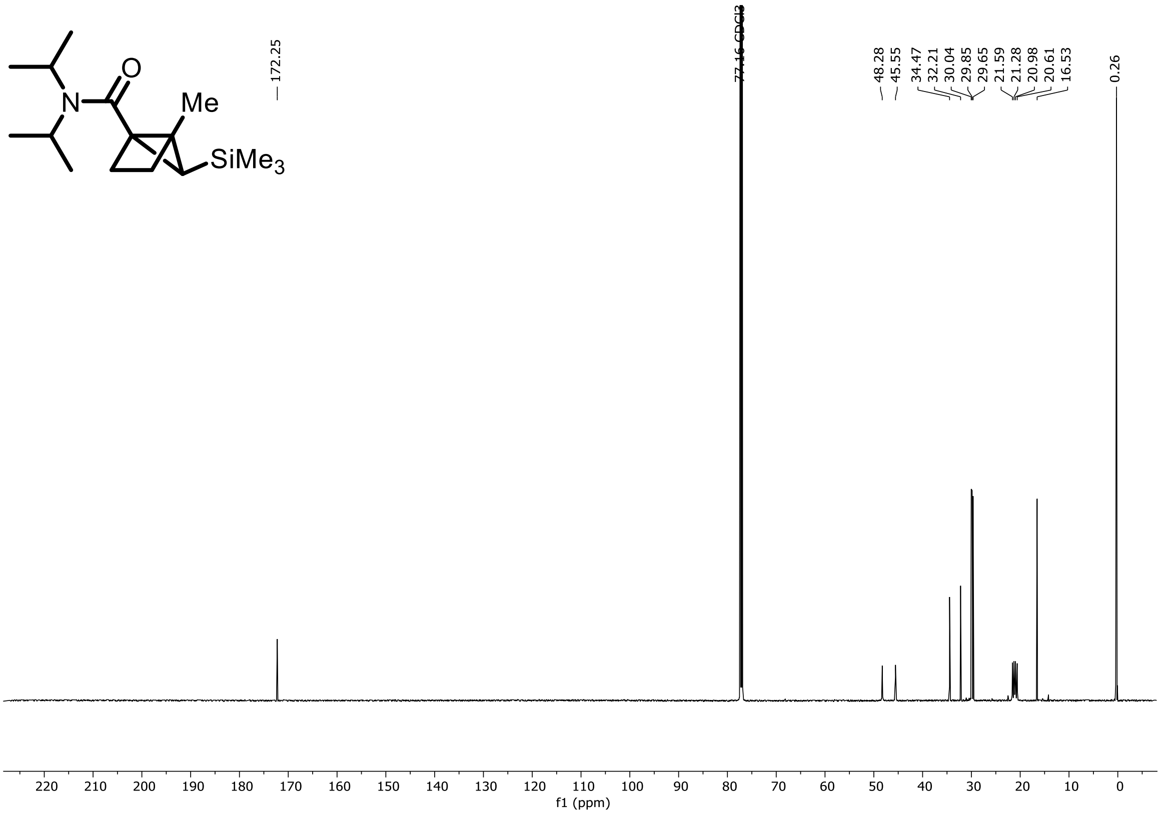

^{1}H NMR (500 MHz, C_6D_6) spectrum of **5f**

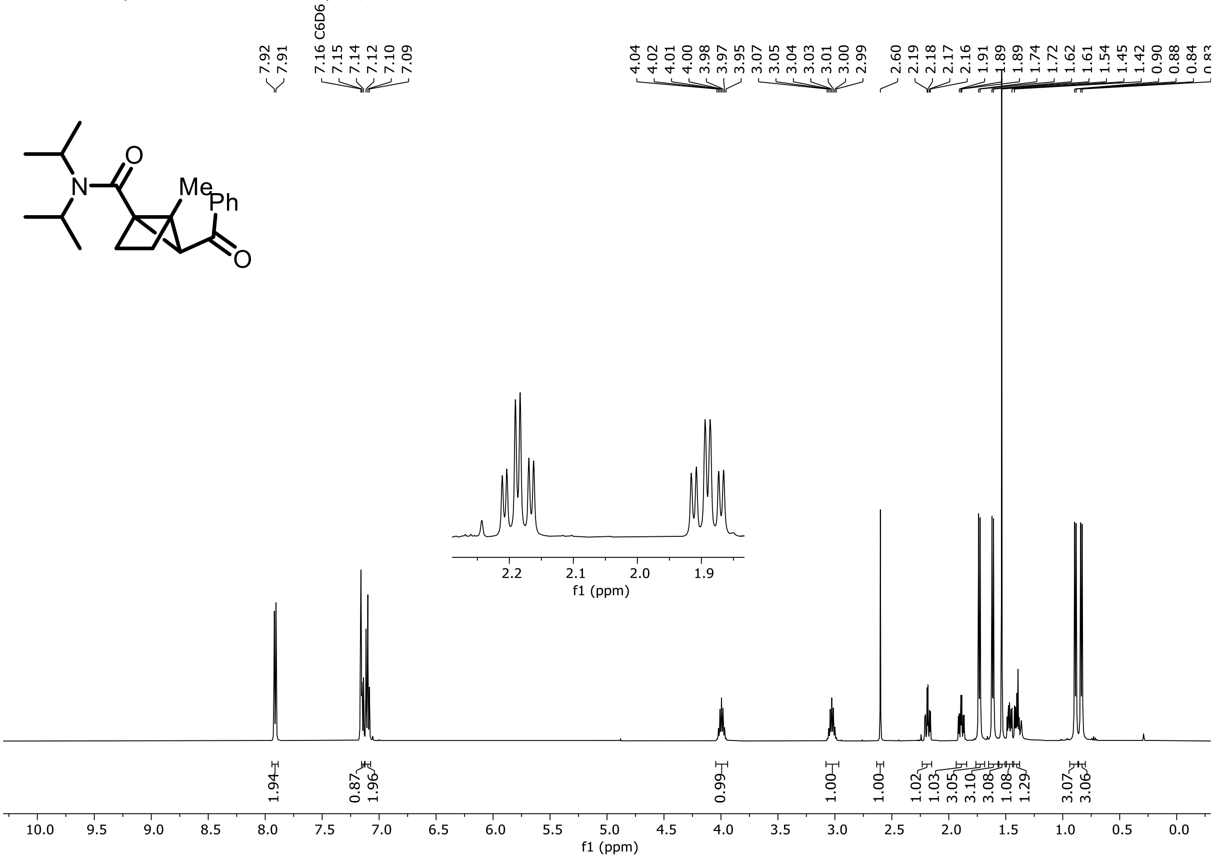

^{13}C NMR (126 MHz, C_6D_6) spectrum of **5f**

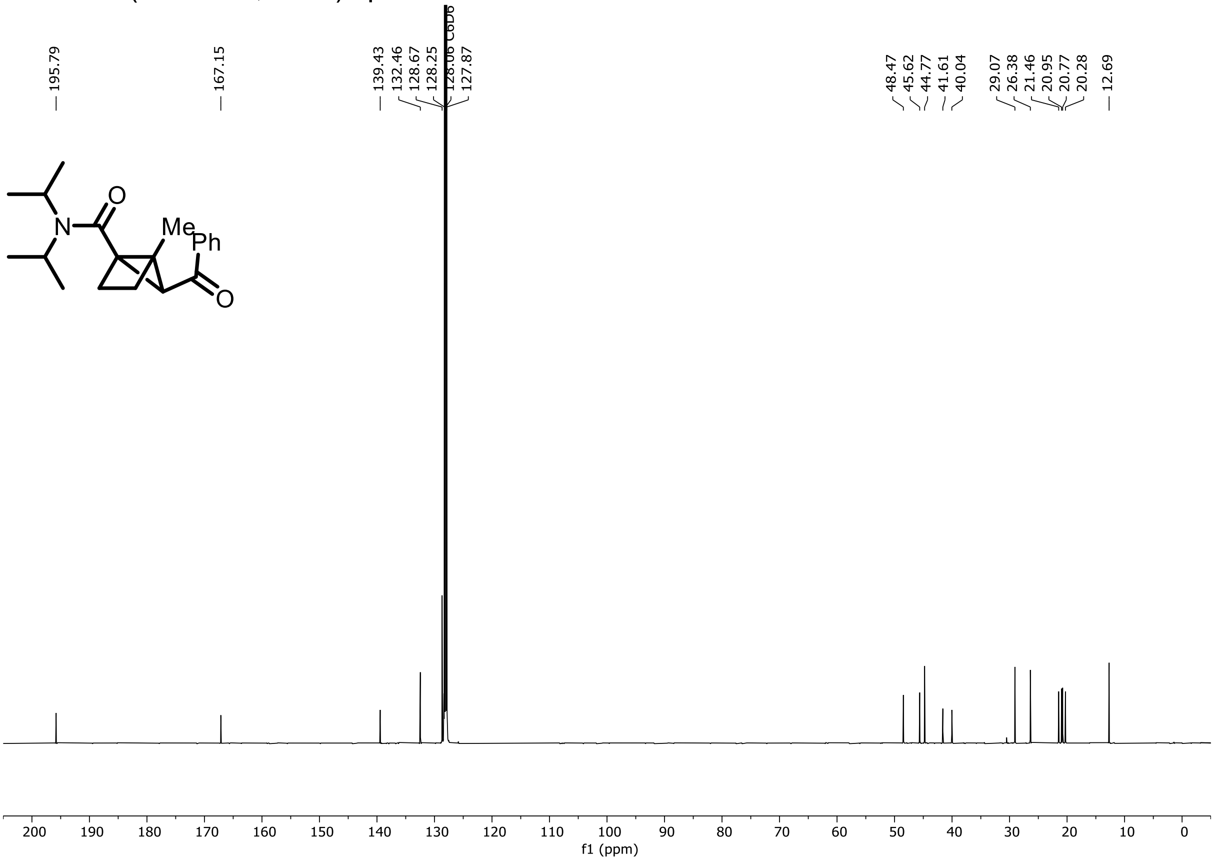

^{1}H NMR (600 MHz, $CDCl_3$) spectrum of **5g**

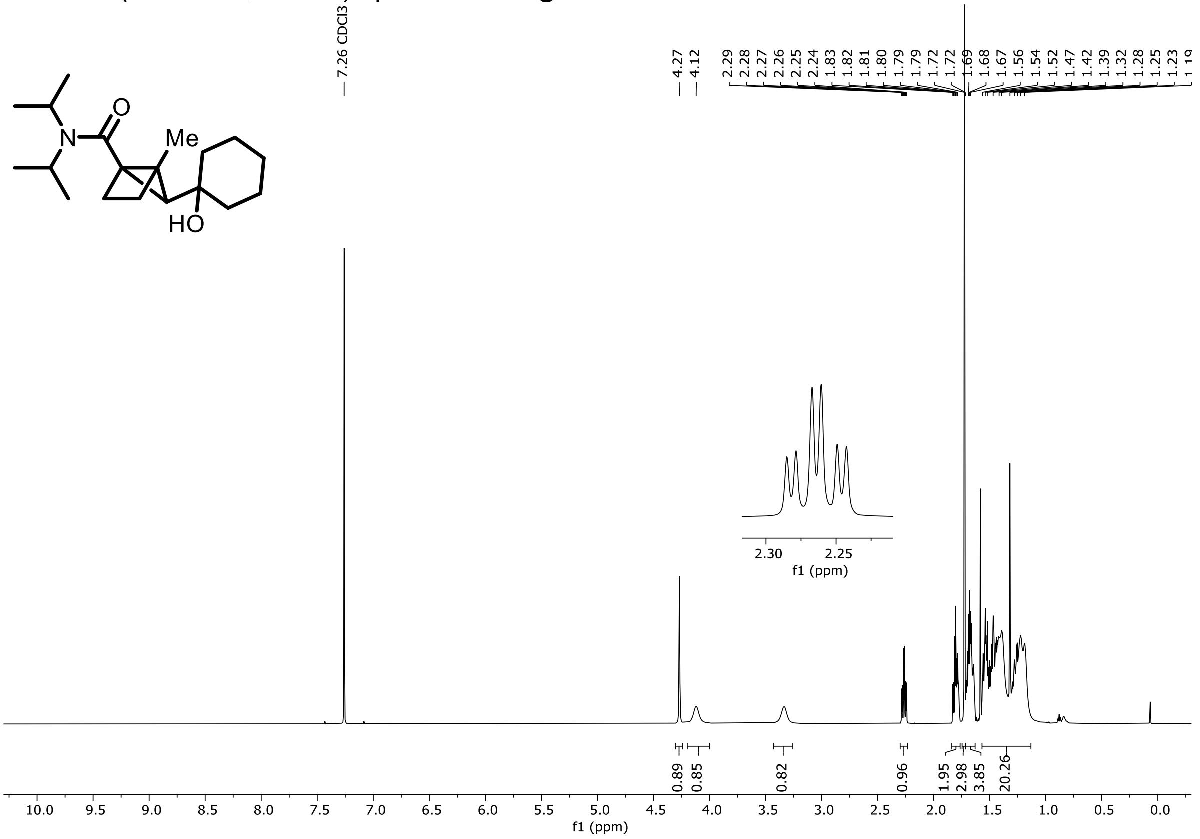

^{13}C NMR (151 MHz, $CDCl_3$) spectrum of **5g**

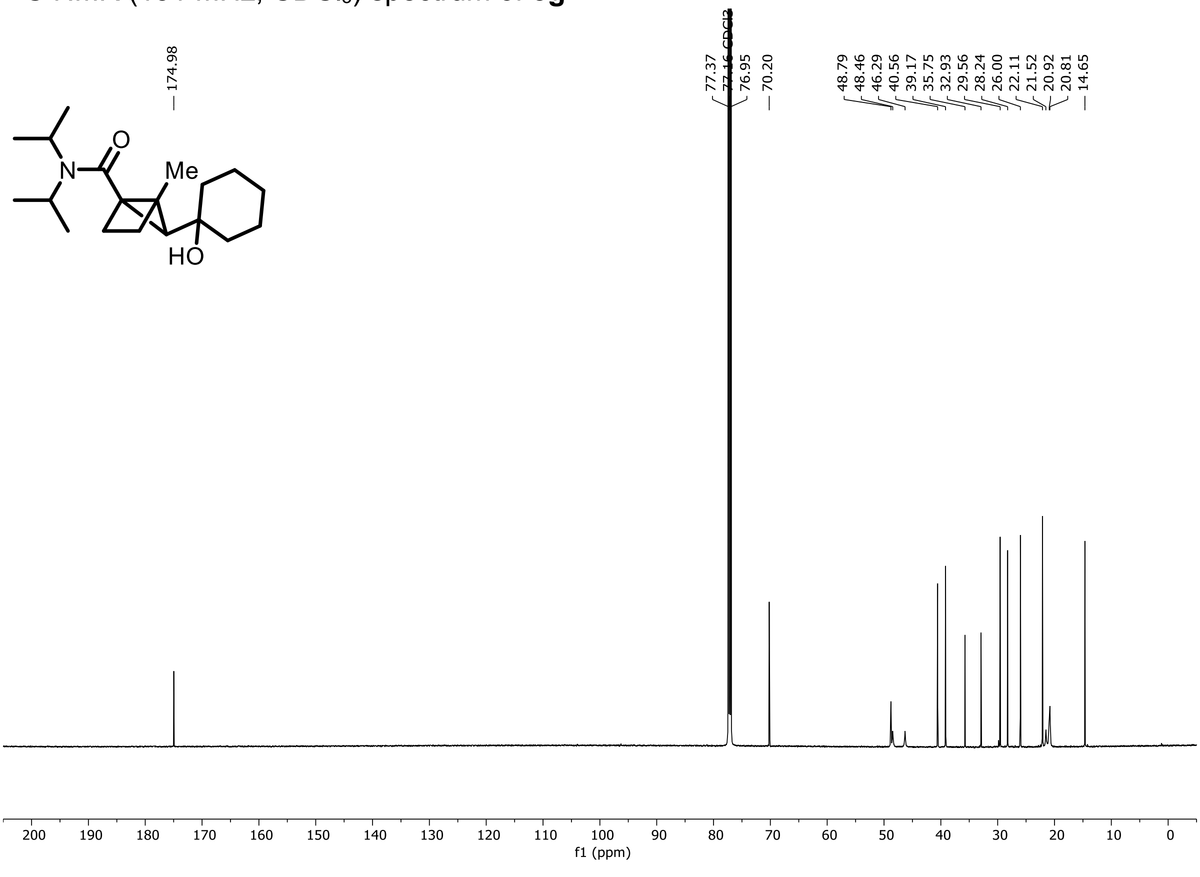

^{1}H NMR (500 MHz, C_6D_6) spectrum of **5h**

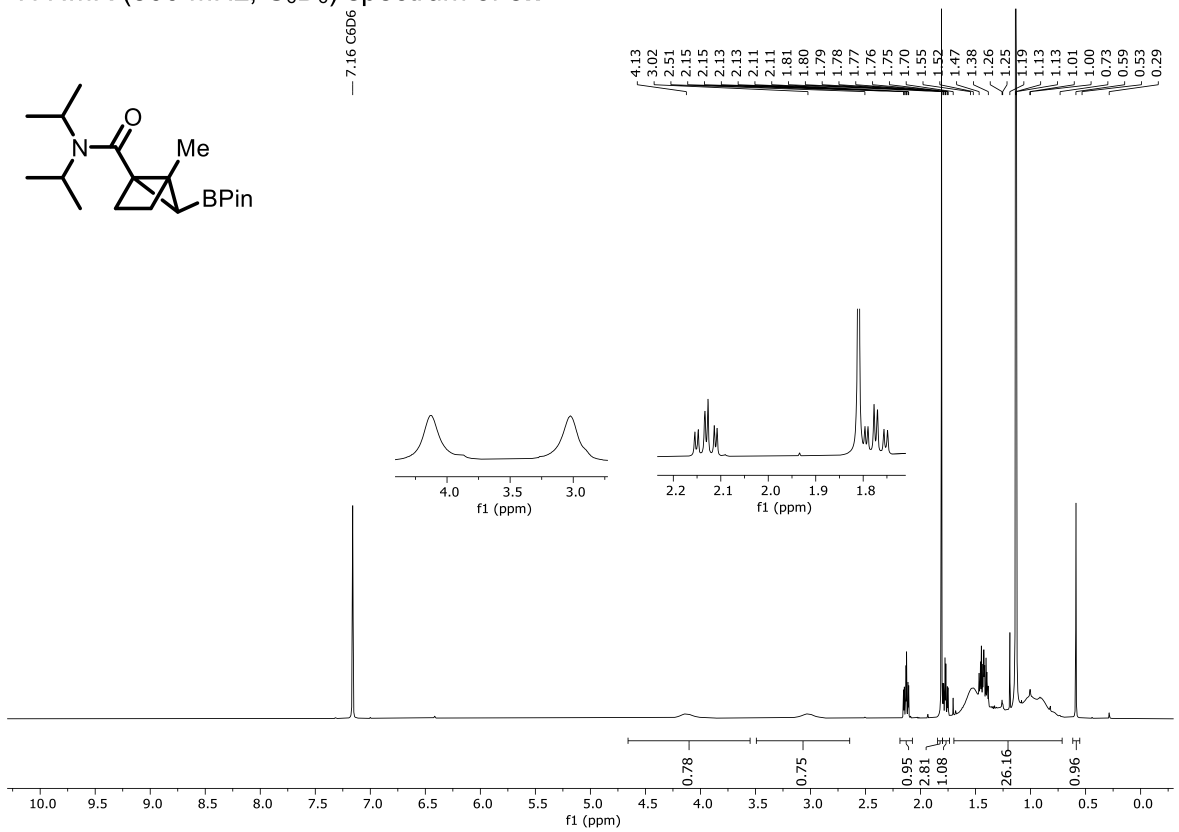

^{13}C NMR (126 MHz, C_6D_6) spectrum of **5h**

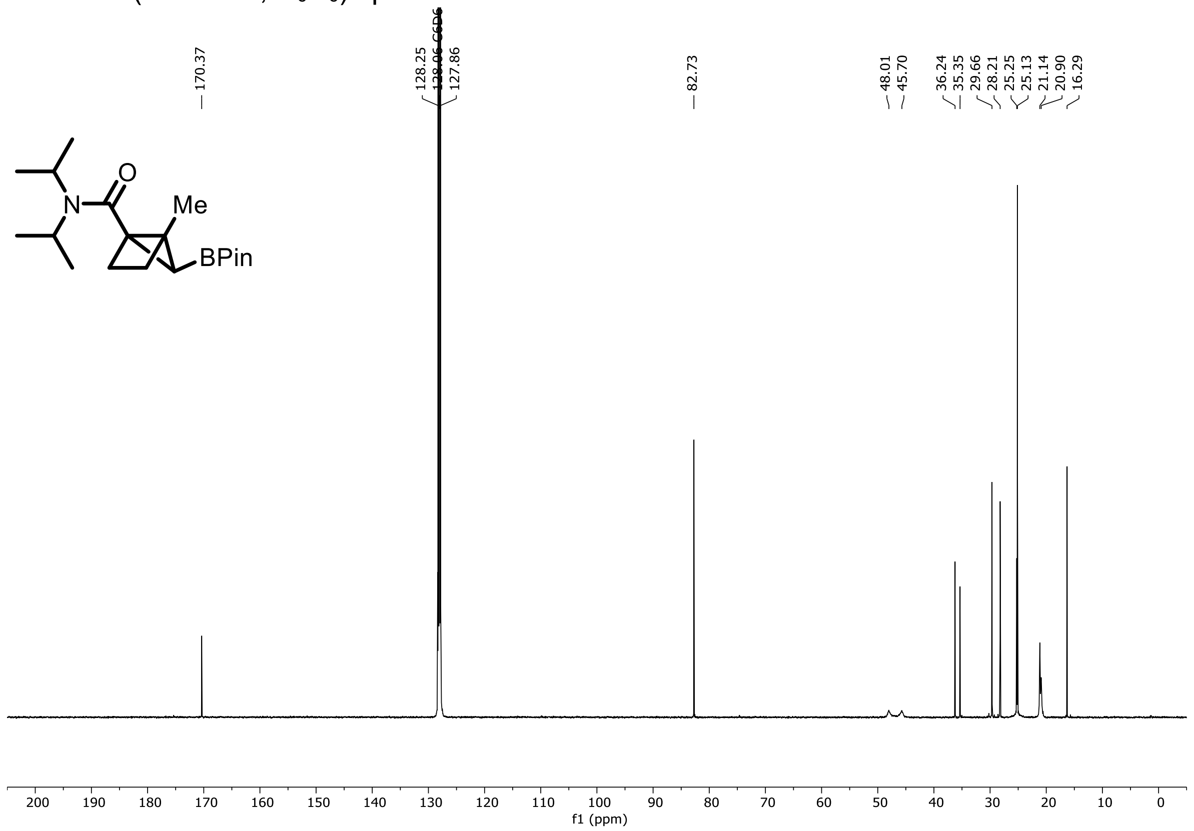

^{11}B NMR (161 MHz, C_6D_6) spectrum of **5h**

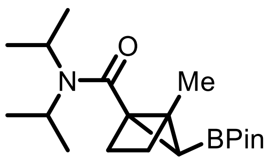

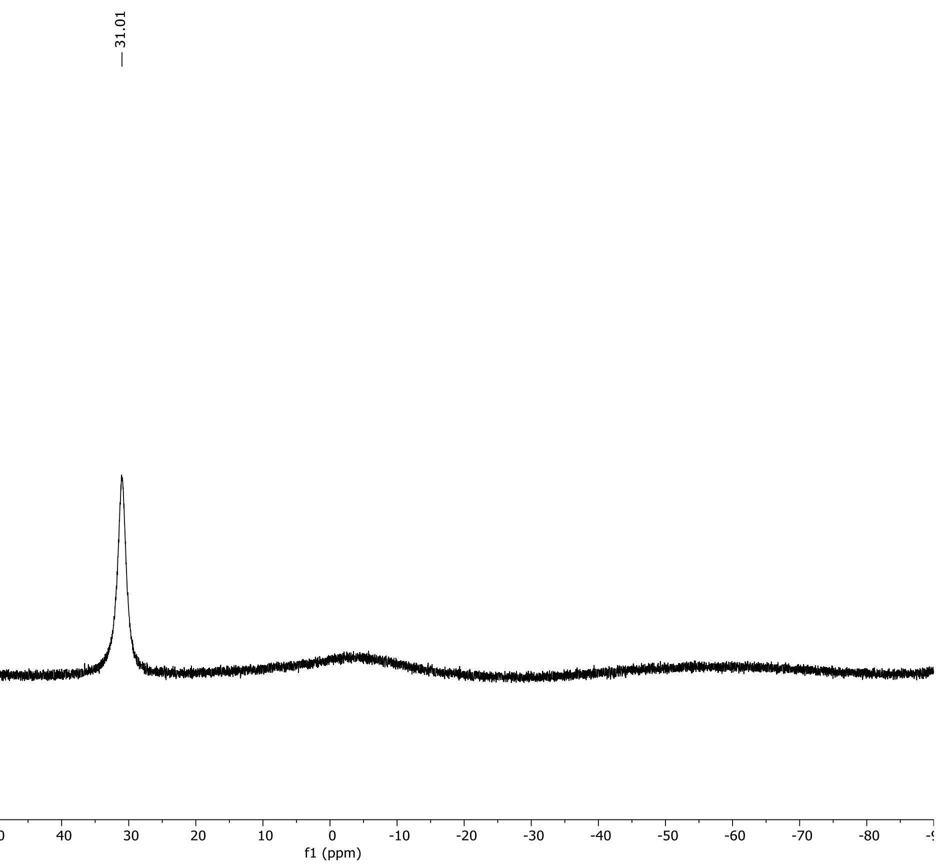

^{1}H NMR (500 MHz, $CDCl_3$) spectrum of **7**

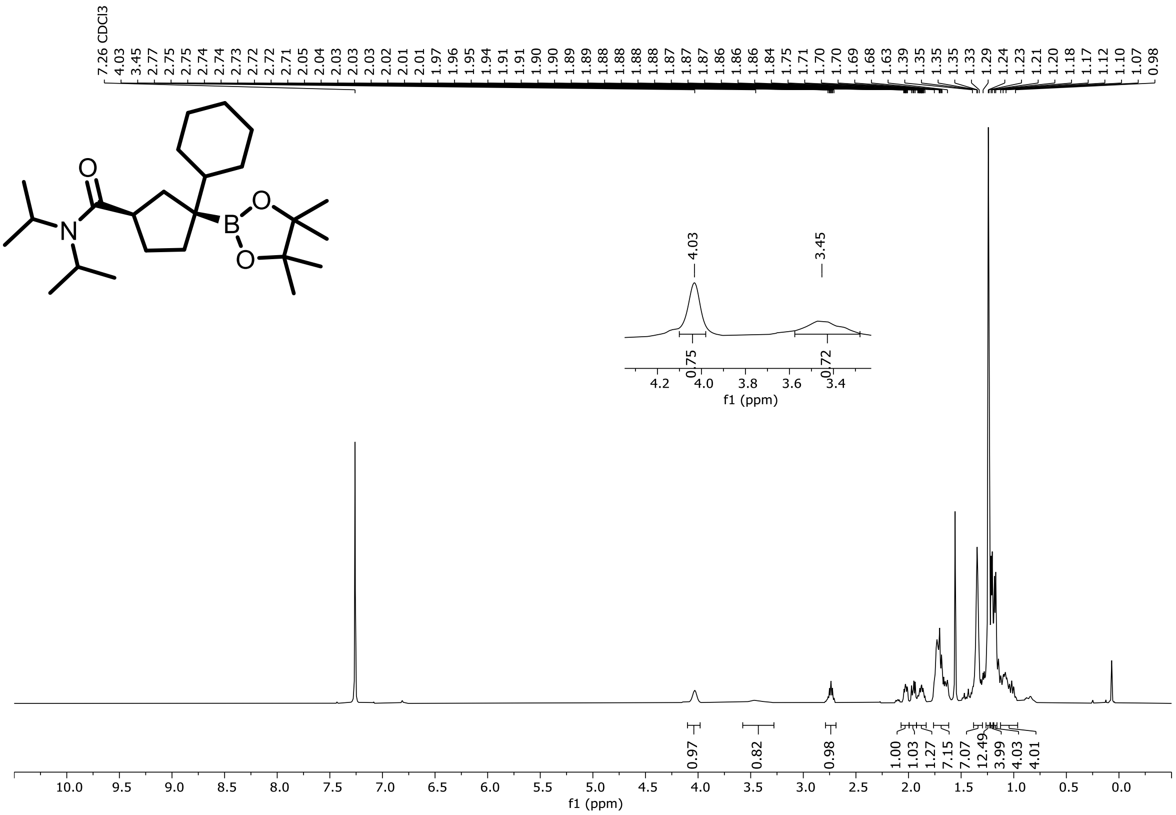

^{13}C NMR (126 MHz, $CDCl_3$) spectrum of **7**

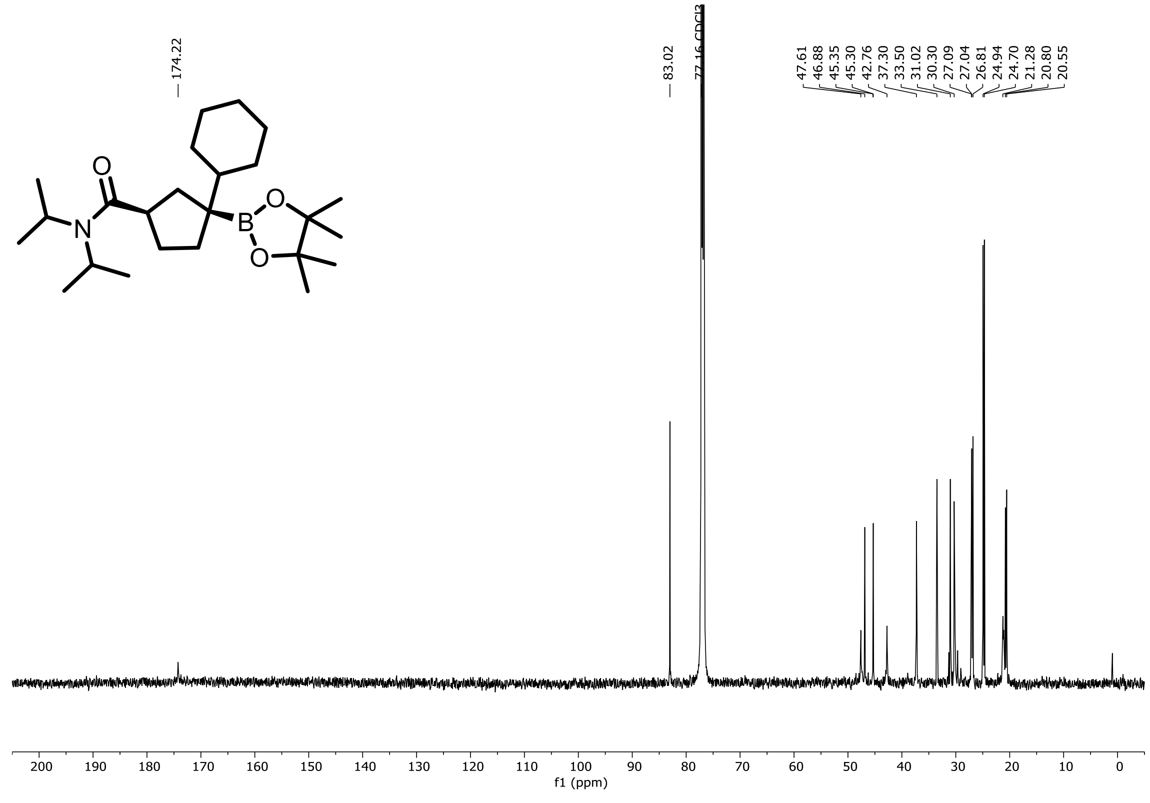

^{1}H NMR (400 MHz, $CDCl_3$) spectrum of **9**

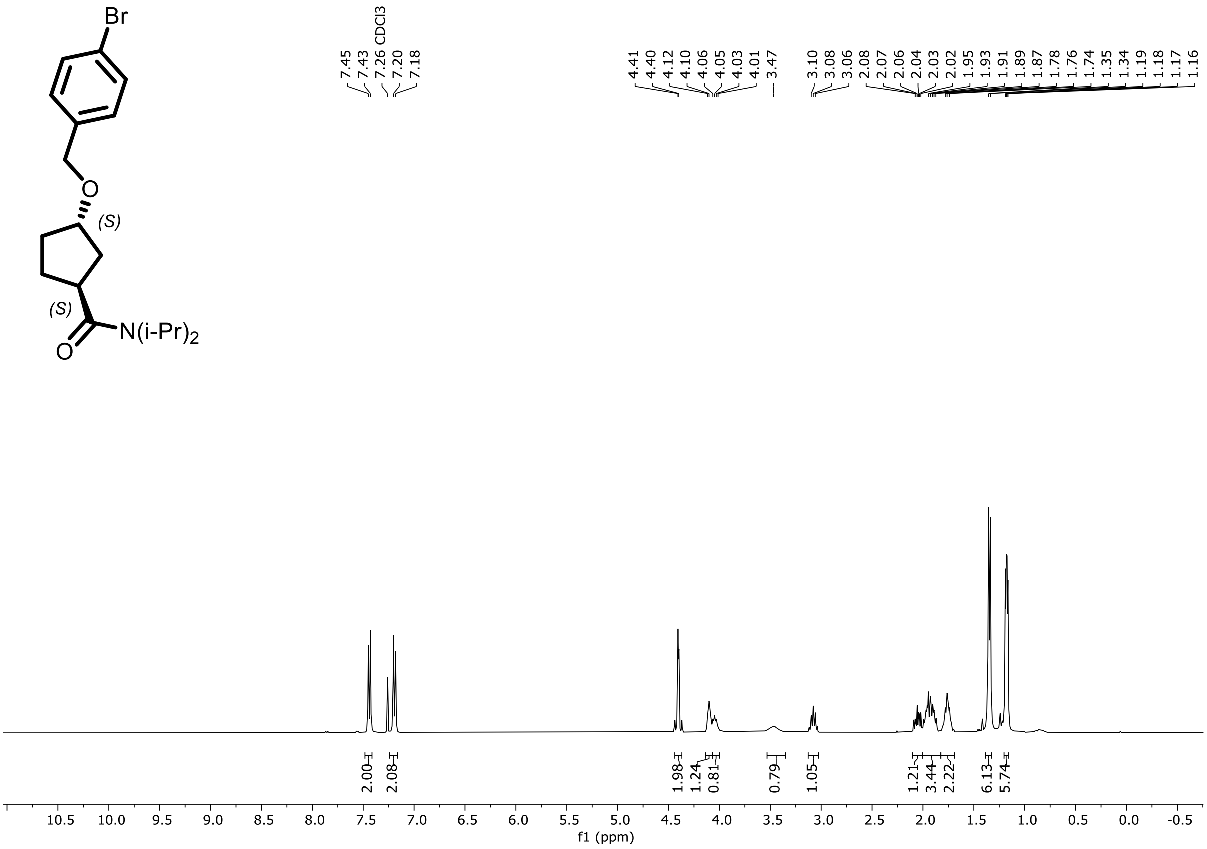

^{13}C NMR (101 MHz, $CDCl_3$) spectrum of **9**

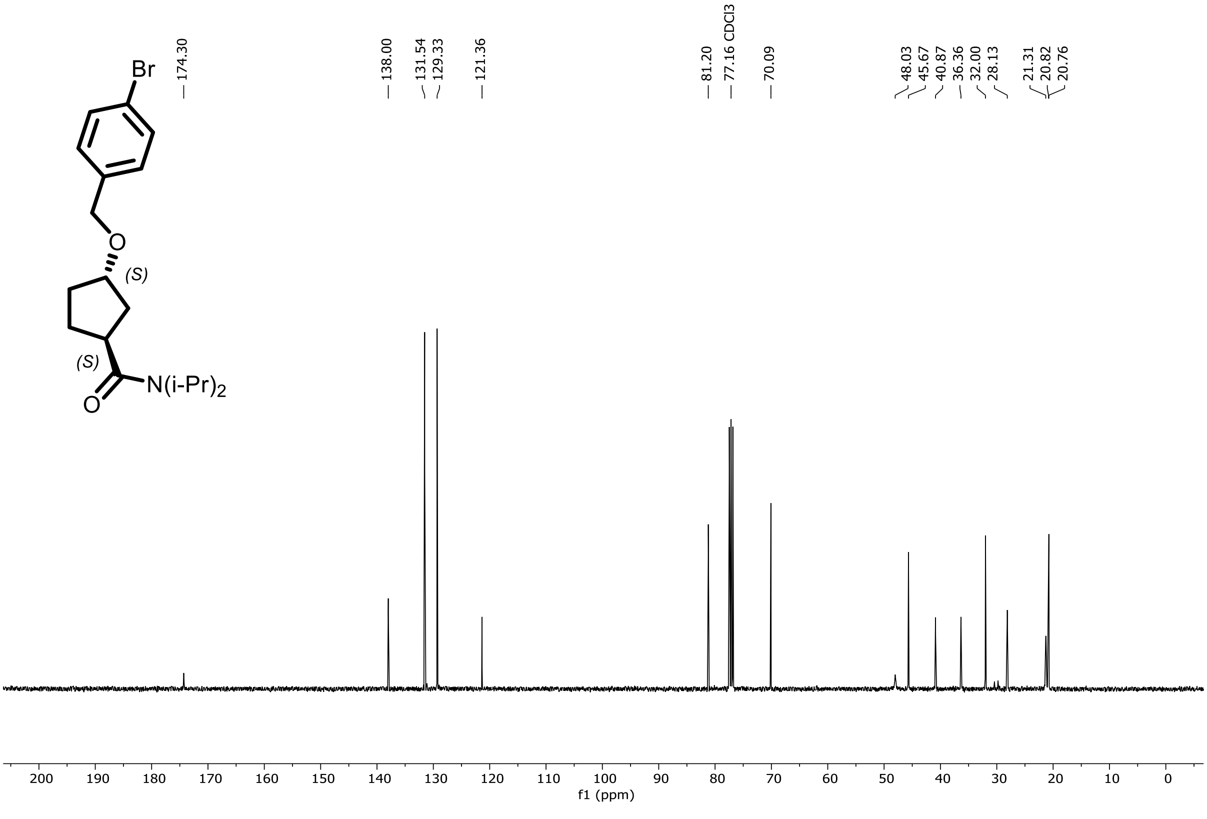

9. Computational details

Theoretical calculations were performed within the Density Functional Theory (DFT) framework.[3] Initial geometries were pre-optimised using the semi-empirical PM6 method. Subsequently, all geometry optimisations were carried out at dispersion corrected ωB97X-D/def2-TZVPP level of theory, using the GAUSSIAN 16[4] suite of programs. Potential energy surface scans were then performed by rotating the specific dihedral angles by 10° increments over 36 steps. Selected minima obtained from scans were further optimised at the same level of theory (ωB97X-D/def2-TZVPP). Vibrational frequency analyses confirmed that all minima exhibited no imaginary frequencies.

9.1 Comparative geometric analysis of computed and X-ray structures

The computationally optimized geometries of both **4i** and **5e** (model structures) and their *ortho*-phenyl analogues (reference structures) were compared with each other and with the structures obtained from single-crystal X-ray diffraction to assess structural features and the effect of substitution on key geometrical parameters. These include the exit vector angles (α), miscellaneous angles (ε_1 and ε_2), the dihedral angle (ϕ), and the distances r_1 and r_2. All geometries were optimized in the gas-phase at ωB97X-D/def2-TZVPP level of theory.

The angle α between the two leaving bonds $\vec{r}$ and $\vec{v}$ was calculated by using the normed scalar product.

$$\cos(\alpha) = \frac{\vec{x} \cdot \vec{y}}{\|\vec{x}\| \cdot \|\vec{y}\|}$$

$$\alpha = arccos\left(\frac{\vec{x} \cdot \vec{y}}{\|\vec{x}\| \cdot \|\vec{y}\|}\right)$$

In case of X-ray structures **4i** and **5e,** these values were obtained using the Platon software package.[5]

Definition of the herein used parameters.

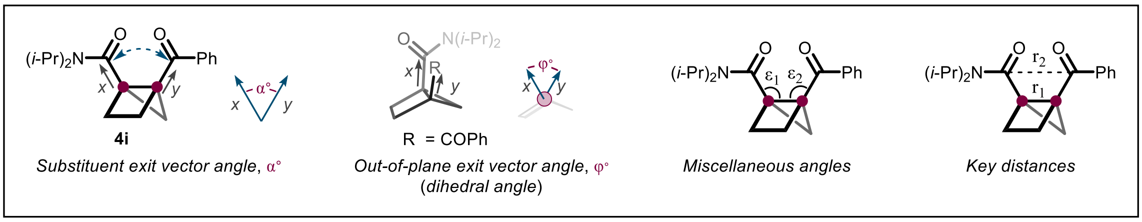

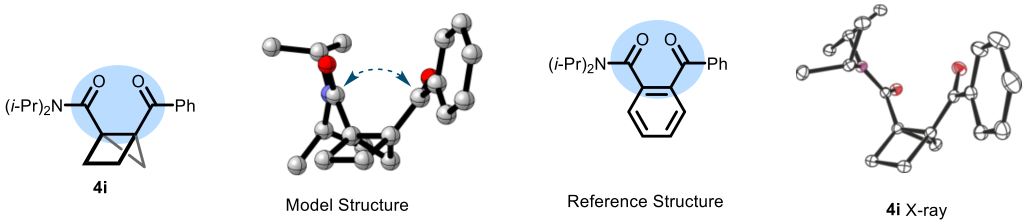

Compound	r_1 (Å)	r_2 (Å)	ε_1 (°)	ε_2 (°)	φ (°)	α (°)
Model Structure*calc*	1.55	3.15	122.2	122.0	13.1	65.3
Reference Structure *calc*	1.40	2.90	119.3	120.7	8.1	60.5
**4ix-ray**	1.57	3.28	128.2	121.8	1.7	70.0

Definition of the herein used parameters

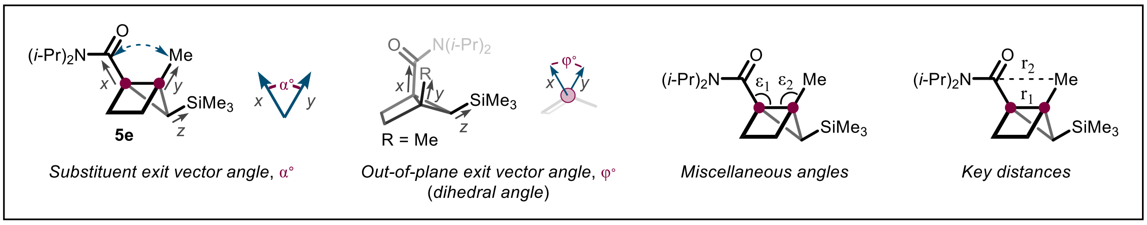

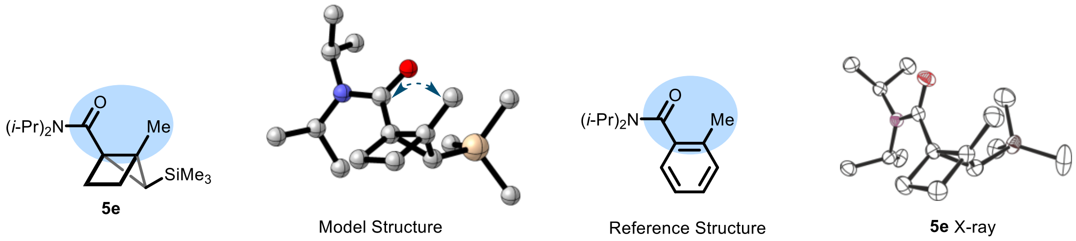

Compound	r_1 (Å)	r_2 (Å)	ε_1 (°)	ε_2 (°)	φ (°)	α (°)
Model Structure*calc*	1.54	3.34	124.2	130.0	7.2	74.6
Reference Structure *calc*	1.40	2.92	119.8	121.1	3.5	60.8
**5ex-ray**	1.53	3.42	127.2	131.0	0.1	78.2

Definition of the herein used parameters.

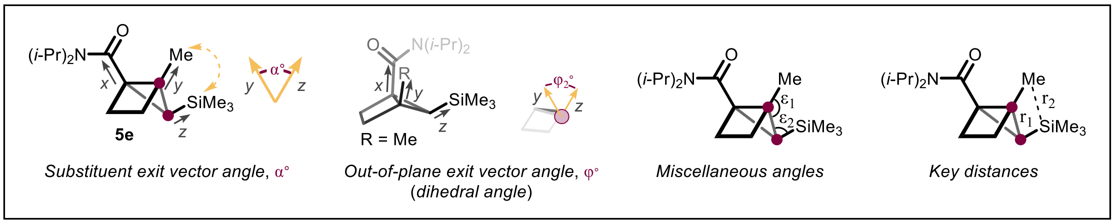

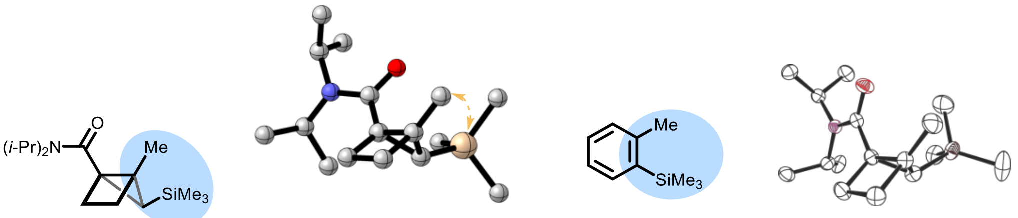

5e | Model Structure | Reference Structure | X-ray **3e**

Compound	r_1 (Å)	r_2 (Å)	ε_1 (°)	ε_2 (°)	φ (°)	α (°)
Model Structure$_{calc}$	1.50	3.52	126.5	126.4	4.4	73.0
Reference Structure $_{calc}$	1.41	3.28	122.1	124.0	2.9	66.1
**5e**$_{X\text{-}ray}$	1.48	3.52	123.6	130.1	1.4	73.8

9.2 Structural superposition of housanes and corresponding *ortho*-phenyl moieties

A structural superposition of the optimized housane scaffolds (model strauctture) and their corresponding *ortho*-phenyl moieties was carried out using *GaussView 6*. The resulting overlays were visualised with *CYLview20*, allowing a direct qualitative comparison of the relative substituent vector orientations.

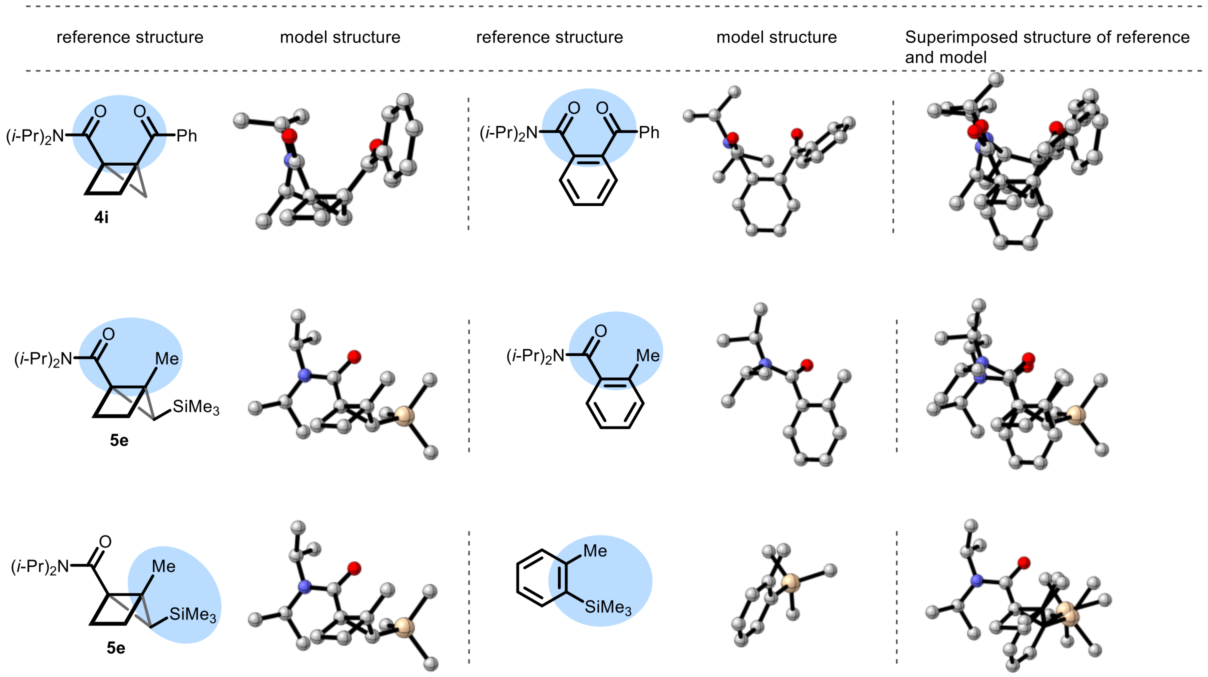

9.3 Cartesian coordinates

Cartesian coordinates (optimized at the ωB97xD/def2-TZVPP level of theory) of all minima discussed in the main text.

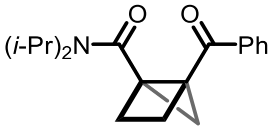

4i

Electronic energy: -944.3015029

Center Number	Atomic Number	Atomic Type	Coordinates (Angstroms) X	Y	Z
1	6	0	-1.312125	-2.138598	-0.849266
2	1	0	-2.001502	-1.704745	-1.568814
3	1	0	-1.709651	-3.094148	-0.503881
4	6	0	0.144361	-2.134115	-1.373940
5	1	0	0.678640	-3.084464	-1.352612
6	1	0	0.222876	-1.693010	-2.363823
7	6	0	0.243662	-1.691213	1.059037
8	1	0	0.486726	-1.051989	1.894808
9	1	0	0.307937	-2.759208	1.240565
10	6	0	-0.906712	-1.155946	0.244766
11	6	0	-1.583121	0.077748	0.716710
12	6	0	0.547815	-1.142853	-0.277614
13	6	0	1.266306	0.133406	-0.613026
14	6	0	-3.012529	0.310715	0.351709
15	6	0	-3.925389	-0.733135	0.244921
16	6	0	-3.443675	1.620805	0.167320
17	6	0	-5.256313	-0.470047	-0.038046
18	1	0	-3.603935	-1.753873	0.403695
19	6	0	-4.767628	1.881037	-0.138305
20	1	0	-2.724601	2.422506	0.264133
21	6	0	-5.676172	0.836028	-0.238365
22	1	0	-5.965056	-1.284684	-0.104396
23	1	0	-5.095011	2.900169	-0.294968
24	1	0	-6.712992	1.040678	-0.470845
25	8	0	-0.975167	0.895169	1.373893
26	8	0	0.705344	0.905967	-1.373611
27	7	0	2.490023	0.401098	-0.075900
28	6	0	3.026409	1.742802	-0.360099
29	1	0	2.152450	2.345599	-0.590361
30	6	0	3.450206	-0.595448	0.425627
31	1	0	4.418615	-0.125570	0.256119
32	6	0	3.480783	-1.891441	-0.379869
33	1	0	4.380258	-2.451875	-0.122970
34	1	0	2.625371	-2.527805	-0.163613
35	1	0	3.501279	-1.685546	-1.450194
36	6	0	3.363568	-0.864429	1.926693
37	1	0	4.279566	-1.353920	2.261717
38	1	0	3.247692	0.062185	2.485264
39	1	0	2.532025	-1.520313	2.171340
40	6	0	3.709050	2.353498	0.857373
41	1	0	3.989823	3.382109	0.630319
42	1	0	3.033796	2.367532	1.712354
43	1	0	4.620032	1.824049	1.142642

44	6	0	3.922135	1.741509	-1.595376
45	1	0	4.821226	1.138981	-1.447456
46	1	0	3.376181	1.351200	-2.453413
47	1	0	4.242889	2.757441	-1.828057

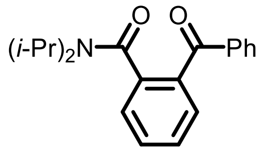

Electronic energy: -981.2504165

Center Number	Atomic Number	Atomic Type	Coordinates (Angstroms) X	Y	Z
1	6	0	1.336437	-0.227722	0.640657
2	6	0	-1.174567	-0.191121	-0.816934
3	6	0	2.754467	-0.493135	0.261150
4	6	0	3.487321	-1.405916	1.015532
5	6	0	3.337781	0.105675	-0.851306
6	6	0	4.794478	-1.699801	0.673822
7	1	0	3.013864	-1.877219	1.865991
8	6	0	4.641929	-0.203551	-1.202801
9	1	0	2.762922	0.793564	-1.455998
10	6	0	5.372507	-1.098974	-0.437022
11	1	0	5.364251	-2.401218	1.268487
12	1	0	5.086604	0.252440	-2.077029
13	1	0	6.393325	-1.334256	-0.708356
14	8	0	0.704203	-1.027019	1.292611
15	8	0	-0.586343	-0.852016	-1.659442
16	7	0	-2.371918	-0.552307	-0.288011
17	6	0	-3.048345	0.051598	0.877359
18	1	0	-3.690117	-0.751106	1.237253
19	6	0	-2.910471	-1.841243	-0.758570
20	1	0	-2.370559	-2.046761	-1.678234
21	6	0	-4.394766	-1.766675	-1.093974
22	1	0	-4.710137	-2.716370	-1.526498
23	1	0	-5.018803	-1.586246	-0.216968
24	1	0	-4.591338	-0.981681	-1.824185
25	6	0	-2.583961	-2.958187	0.228098
26	1	0	-1.511491	-2.990467	0.416046
27	1	0	-3.097232	-2.822759	1.182541
28	1	0	-2.895040	-3.920545	-0.179771
29	6	0	-2.145685	0.405881	2.055722
30	1	0	-2.771931	0.547685	2.937665
31	1	0	-1.428131	-0.386975	2.253413
32	1	0	-1.595403	1.330826	1.892680
33	6	0	-3.972352	1.211720	0.511231
34	1	0	-3.407895	2.119025	0.304511
35	1	0	-4.583520	0.974557	-0.358158
36	1	0	-4.638342	1.424075	1.349039
37	6	0	1.379184	2.267854	0.485957
38	6	0	0.811942	3.483056	0.134507
39	6	0	-0.400887	3.505274	-0.532790
40	6	0	-1.061603	2.317555	-0.808843
41	6	0	-0.527334	1.102891	-0.408047

42	6	0	0.721224	1.075406	0.215169
43	1	0	2.343483	2.243609	0.976890
44	1	0	1.327685	4.406065	0.362020
45	1	0	-0.831794	4.446307	-0.847730
46	1	0	-1.993654	2.332370	-1.356396

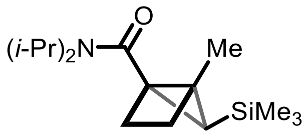

5e

Electronic energy: -1047.9310777

Center Number	Atomic Number	Atomic Type	Coordinates (Angstroms) X	Y	Z
1	6	0	-0.997139	3.028552	0.595910
2	1	0	-0.704103	3.614535	1.465676
3	1	0	-1.763819	3.573727	0.044154
4	6	0	0.219607	2.532269	-0.218473
5	1	0	0.255789	2.818034	-1.270796
6	1	0	1.155428	2.817237	0.257011
7	6	0	-1.607565	0.827592	-0.319388
8	1	0	-1.917110	1.443971	-1.164956
9	6	0	-1.348743	1.583014	0.946306
10	6	0	-0.165446	1.084998	0.098519
11	6	0	0.717166	-0.049987	0.478565
12	8	0	0.307288	-0.860560	1.302472
13	7	0	1.982873	-0.142411	-0.030673
14	6	0	2.935637	-1.056764	0.629344
15	1	0	3.900363	-0.796356	0.188298
16	6	0	2.419674	0.273232	-1.377163
17	1	0	3.098705	-0.527593	-1.679865
18	6	0	3.249390	1.554441	-1.367917
19	1	0	3.756004	1.676247	-2.326453
20	1	0	2.634993	2.435148	-1.199726
21	1	0	4.009241	1.513223	-0.586820
22	6	0	1.330766	0.272128	-2.441900
23	1	0	0.818432	-0.687527	-2.459110
24	1	0	0.587944	1.051716	-2.304068
25	1	0	1.797626	0.426069	-3.415305
26	6	0	3.075363	-0.803860	2.125510
27	1	0	3.940425	-1.353168	2.499539
28	1	0	3.237608	0.257194	2.319195
29	1	0	2.194270	-1.126959	2.672235
30	6	0	2.654811	-2.521834	0.303884
31	1	0	1.719316	-2.836369	0.760853
32	1	0	2.584624	-2.674828	-0.774816
33	1	0	3.461542	-3.152408	0.680152
34	6	0	-1.780601	1.186736	2.323500
35	1	0	-2.838322	1.415381	2.473994
36	1	0	-1.616878	0.130100	2.505948
37	1	0	-1.208948	1.745880	3.066238
38	6	0	-4.037300	-0.496207	-1.381923
39	1	0	-4.610699	-1.410737	-1.545297
40	1	0	-4.666076	0.192808	-0.814249

41	1	0	-3.845315	-0.044633	-2.357217
42	6	0	-2.875047	-1.727871	1.132850
43	1	0	-3.531328	-1.126596	1.762389
44	1	0	-3.403259	-2.653350	0.891056
45	1	0	-1.980237	-1.980073	1.699900
46	6	0	-1.403172	-2.019536	-1.512397
47	1	0	-1.921356	-2.973642	-1.629622
48	1	0	-1.231463	-1.612660	-2.509972
49	1	0	-0.438577	-2.219124	-1.043712
50	14	0	-2.438404	-0.861094	-0.464364

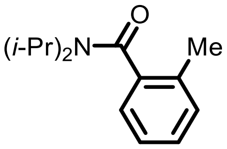

Electronic energy: -676.1799704

Center Number	Atomic Number	Atomic Type	Coordinates (Angstroms) X	Y	Z
1	6	0	0.257367	-0.569011	-0.780992
2	8	0	0.408783	-1.457300	-1.605146
3	7	0	1.308351	0.043951	-0.163714
4	6	0	1.246240	0.937857	1.006855
5	1	0	2.279331	0.979303	1.347486
6	6	0	2.651174	-0.389647	-0.582011
7	1	0	2.485729	-0.958258	-1.492543
8	6	0	3.277061	-1.330724	0.442511
9	1	0	4.235712	-1.699780	0.076865
10	1	0	3.458347	-0.835038	1.398730
11	1	0	2.625396	-2.187176	0.614450
12	6	0	3.552731	0.791825	-0.921823
13	1	0	3.084719	1.432492	-1.669058
14	1	0	3.797086	1.402136	-0.050457
15	1	0	4.492415	0.422573	-1.333100
16	6	0	0.851857	2.376812	0.681916
17	1	0	1.116245	3.022557	1.520697
18	1	0	1.378977	2.734045	-0.202118
19	1	0	-0.218278	2.472769	0.513328
20	6	0	0.450981	0.382232	2.184392
21	1	0	-0.620615	0.386977	1.996583
22	1	0	0.763810	-0.635943	2.415257
23	1	0	0.637372	1.000415	3.063475
24	6	0	-1.619000	1.061176	-1.023753
25	6	0	-2.941432	1.440941	-0.853797
26	6	0	-3.795863	0.623971	-0.134390
27	6	0	-3.330895	-0.575297	0.385305
28	6	0	-1.148225	-0.125720	-0.482437
29	1	0	-0.944970	1.689584	-1.590910
30	1	0	-3.298541	2.367957	-1.281773
31	1	0	-4.828326	0.911422	0.014374
32	1	0	-4.008311	-1.218157	0.933874
33	6	0	-2.014092	-0.979886	0.207476
34	6	0	-1.541875	-2.307743	0.727847
35	1	0	-2.291592	-2.764234	1.372056
36	1	0	-1.332195	-2.984584	-0.100651

37	1	0	-0.617208	-2.209041	1.298004

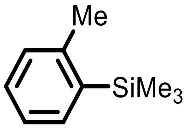

Electronic energy: -680.2694921

Center Number	Atomic Number	Atomic Type	Coordinates (Angstroms) X	Y	Z
1	6	0	0.626748	2.336852	-0.018888
2	1	0	0.285031	2.600074	-1.021606
3	1	0	-0.225559	2.440088	0.651953
4	1	0	1.373520	3.071920	0.278240
5	6	0	-2.186559	0.952432	-1.349309
6	1	0	-1.732439	0.720206	-2.314131
7	1	0	-3.263629	0.797864	-1.442035
8	1	0	-2.021815	2.010091	-1.145716
9	6	0	-2.119924	0.430337	1.676053
10	1	0	-1.797990	1.444703	1.913266
11	1	0	-3.211558	0.414700	1.703688
12	1	0	-1.755250	-0.226262	2.467746
13	6	0	-2.160137	-1.886473	-0.283344
14	1	0	-3.251296	-1.859477	-0.310529
15	1	0	-1.814801	-2.300351	-1.232133
16	1	0	-1.869110	-2.573902	0.512395
17	14	0	-1.501854	-0.153919	0.002974
18	6	0	2.585971	0.800902	0.017665
19	6	0	3.185052	-0.448620	0.007158
20	6	0	2.394863	-1.583147	-0.018141
21	6	0	1.014649	-1.449345	-0.029932
22	6	0	0.386646	-0.201507	-0.018233
23	6	0	1.202611	0.944424	0.002728
24	1	0	3.207741	1.688068	0.038276
25	1	0	4.264124	-0.531281	0.019917
26	1	0	2.845678	-2.566821	-0.026617
27	1	0	0.413783	-2.349892	-0.048891

10. Crystallographic data

Low temperature single crystal X-ray diffraction data for **4i**, **4j**, **4k, 5e** and **9** were collected using a Rigaku Synergy-DW diffractometer (EP/V028995/1) at 100 K. The single crystal was affixed to a cryoloop using Fomblin® Y oil. In all cases, Cu-Kα (λ = 1.54184 Å) radiation was used and the instrument was equipped with a nitrogen gas Oxford Cryosystems Cryostream unit. Raw frame data were reduced using CrysAlisPro and the structure was solved using 'Superflip'[6] before refinement with CRYSTALS.[7] Refinement was by full-matrix least-squares with anisotropic atomic displacement parameters freely refined for all non-hydrogen atoms. In general, hydrogen atoms were visible in the difference Fourier map. Hydrogens bound to carbon were positioned at calculated positions and refined separately with restraints before being included in the refinement using a riding model.[8] Final refinement statistics are given in the table below and further details, are documented in the CIF; Crystallographic data have been deposited with the Cambridge Crystallographic Data Centre CCDC 2440671 (**4i**), 2440673 (**4j**), 2440672 (**4k**) 2440674 (**5e**) and 2532699 (**9**) can be obtained via www.ccdc.cam.ac.uk/data_request/cif.

10.1 Solid-state structures

Solid-state structure of compound **4i** (CCDC 2440671), Thermal ellipsoids drawn at 50% probability. Carbon: black; oxygen: red; nitrogen purple.

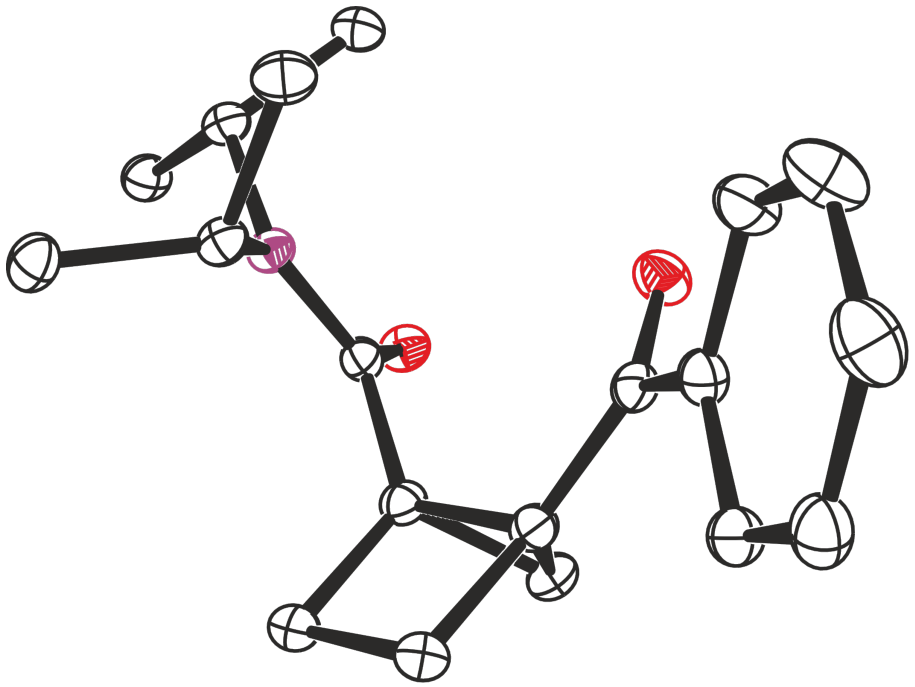

Solid-state structure of compound **4j** (CCDC 244067), Thermal ellipsoids drawn at 50% probability. Carbon: black; oxygen: red; nitrogen: purple and bromine: dark red.

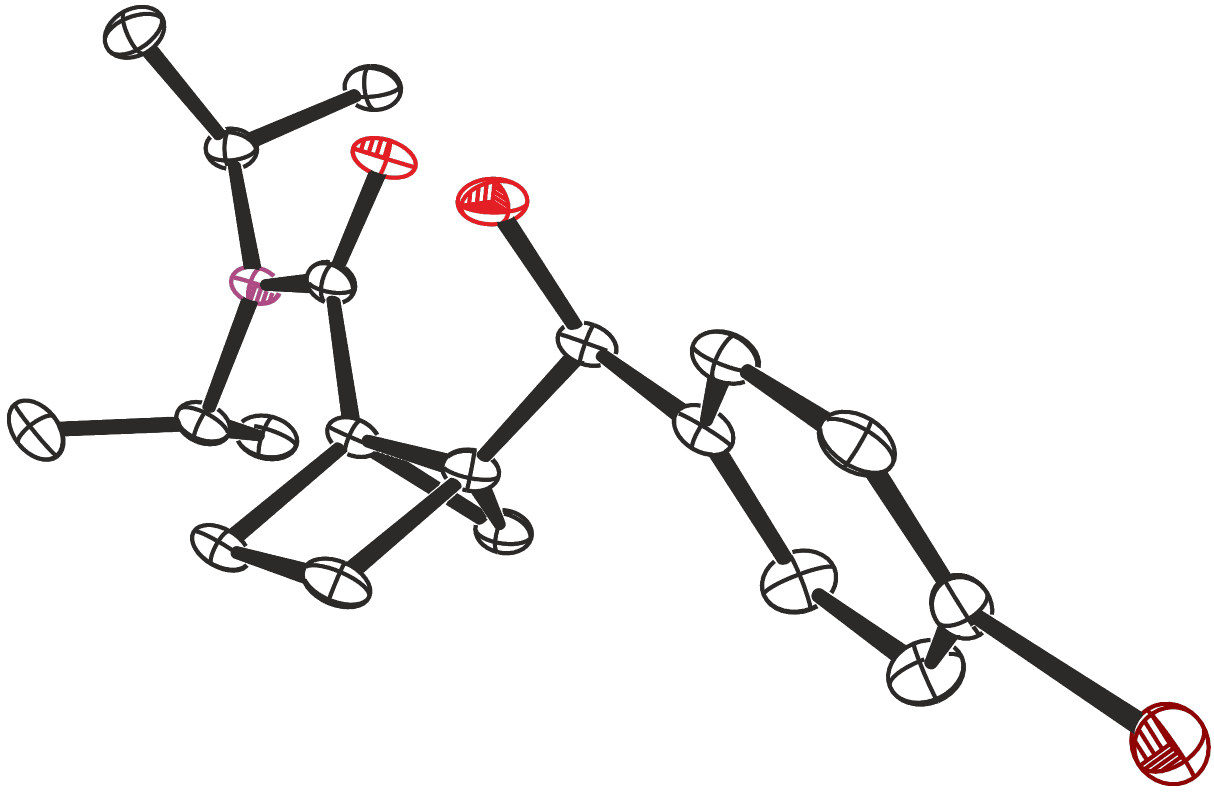

Solid-state structure of compound **4k** (CCDC 2440672), Thermal ellipsoids drawn at 50% probability. Carbon: black; oxygen: red; nitrogen: purple.

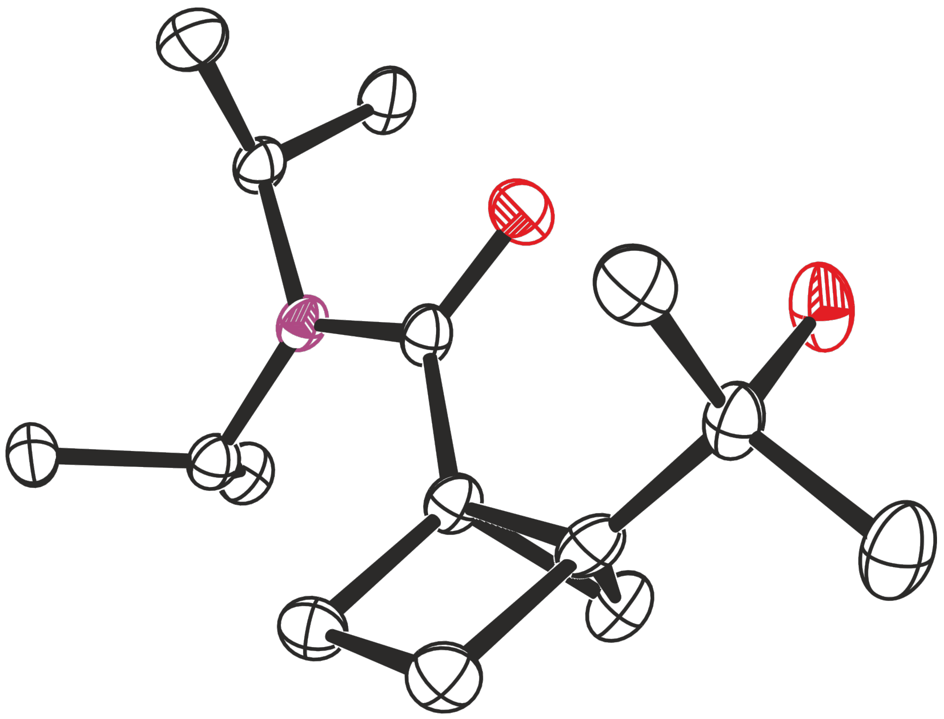

Solid-state structure of compound **5e** (CCDC 2440674), Thermal ellipsoids drawn at 50% probability. Carbon: black; oxygen: red; nitrogen: purple and silicon: wine red.

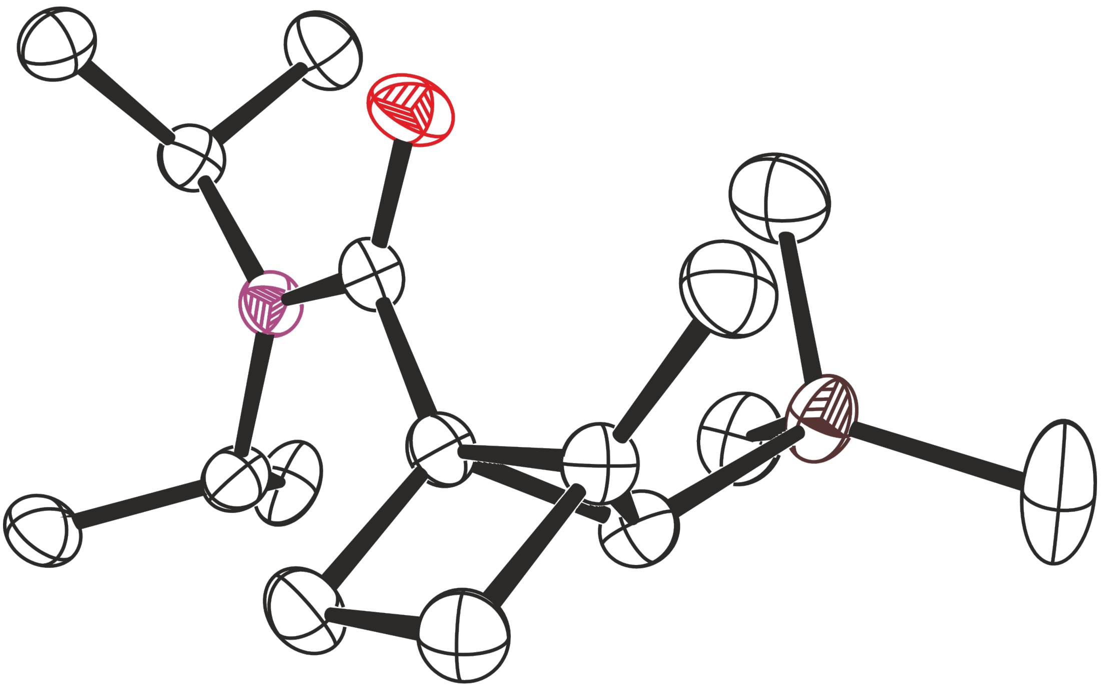

Solid-state structure of compound (+)-**9** (CCDC 2532699), Thermal ellipsoids drawn at 50% probability. Carbon: black; oxygen: red; nitrogen: purple and bromine: yellow.

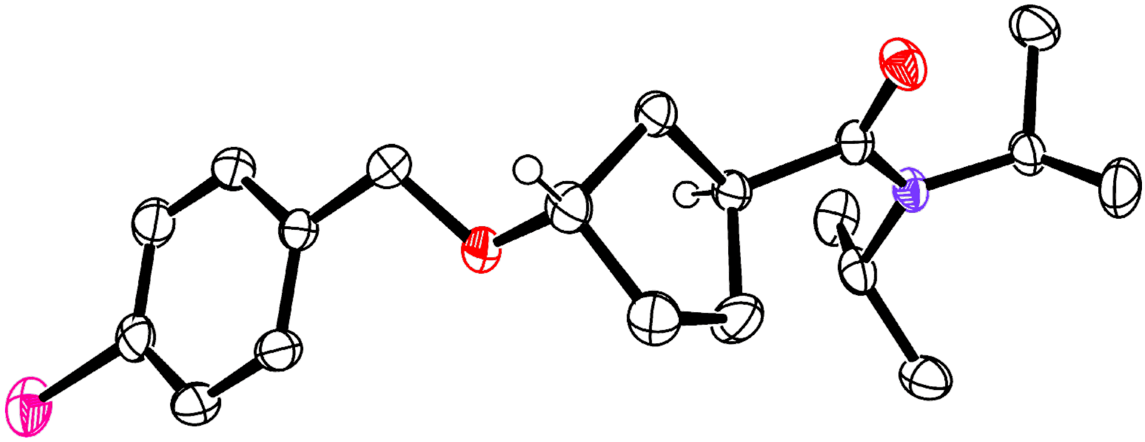

10.2 X-ray refinement data

Crystal data and structure refinement for **4i**

CCDC number	2440671	
Empirical formula	$C_{19}H_{25}NO_2$	
Formula weight	299.41	
Temperature	100 K	
Wavelength	1.54184 Å	
Crystal system / Space group	Monoclinic / P 2_1/n	
Unit cell dimensions	a = 8.24820(10) Å	a = 90°.
	b = 11.35060(10) Å	b = 100.5705(11)°.
	c = 17.9132(2) Å	g = 90°.
Volume	1648.61(3) $Å^3$	
Z	4	
Density (calculated)	1.206 Mg/m^3	
Absorption coefficient	0.608 mm^{-1}	
F(000)	648	
Crystal size	0.05 x 0.05 x 0.03 mm^3	
Theta range for data collection	4.635 to 75.706°.	
Index ranges	-10<=h<=10, -14<=k<=14, -19<=l<=22	
Reflections collected	69489	
Independent reflections	3401 [R(int) = 0.032]	
Completeness to theta = 74.191°	99.7 %	
Absorption correction	Semi-empirical from equivalents	
Max. and min. transmission	0.98 and 0.85	
Refinement method	Full-matrix least-squares on F^2	
Data / restraints / parameters	3401 / 0 / 200	
Goodness-of-fit on F^2	0.9962	
Final R indices [I>2sigma(I)]	R1 = 0.0312, wR2 = 0.0784	
R indices (all data)	R1 = 0.0343, wR2 = 0.0807	
Extinction coefficient	21(4)	
Largest diff. peak and hole	0.26 and -0.19 e.$Å^{-3}$	

Crystal data and structure refinement for **4j**

CCDC number	244067	
Empirical formula	$C_{19}H_{26}BrNO_2$	
Formula weight	380.32	
Temperature	100 K	
Wavelength	0.71073 Å	
Crystal system /Space group	Monoclinic / P 2_1/c	
Unit cell dimensions	a = 13.8214(2) Å	a = 90°.
	b = 11.6960(2) Å	b = 93.6069(18)°.
	c = 11.4377(2) Å	g = 90°.
Volume	1845.30(5) $Å^3$	
Z	4	
Density (calculated)	1.369 Mg/m^3	
Absorption coefficient	2.237 mm^{-1}	
F(000)	792	
Crystal size	0.15 x 0.10 x 0.05 mm^3	
Theta range for data collection	3.429 to 27.479°.	
Index ranges	-17<=h<=17, -15<=k<=15, -14<=l<=14	
Reflections collected	64613	
Independent reflections	4219 [R(int) = 0.055]	
Completeness to theta = 27.479°	99.9 %	
Absorption correction	Semi-empirical from equivalents	
Max. and min. transmission	0.89 and 0.65	
Refinement method	Full-matrix least-squares on F^2	
Data / restraints / parameters	4213 / 0 / 208	
Goodness-of-fit on F^2	1.0101	
Final R indices [I>2sigma(I)]	R1 = 0.0411, wR2 = 0.1034	
R indices (all data)	R1 = 0.0510, wR2 = 0.1115	
Largest diff. peak and hole	2.01 and -0.43 e.$Å^{-3}$	

Crystal data and structure refinement for **4k**

CCDC	2440672	
Empirical formula	$C_{15}H_{27}NO_2$	
Formula weight	253.38	
Temperature	100 K	
Wavelength	1.54184 Å	
Crystal system / Space group	Monoclinic / P 2_1/c	
Unit cell dimensions	a = 20.1975(3) Å	a = 90°.
	b = 10.78280(10) Å	b = 111.0847(15)°.
	c = 14.8934(2) Å	g = 90°.
Volume	3026.41(7) $Å^3$	
Z	8	
Density (calculated)	1.112 Mg/m^3	
Absorption coefficient	0.568 mm^{-1}	
F(000)	1120	
Crystal size	0.08 x 0.05 x 0.02 mm^3	
Theta range for data collection	4.693 to 76.405°.	
Index ranges	-25<=h<=25, -13<=k<=13, -18<=l<=18	
Reflections collected	122445	
Independent reflections	6250 [R(int) = 0.046]	
Completeness to theta = 74.113°	99.8 %	
Absorption correction	Semi-empirical from equivalents	
Max. and min. transmission	0.99 and 0.80	
Refinement method	Full-matrix least-squares on F^2	
Data / restraints / parameters	6246 / 0 / 325	
Goodness-of-fit on F^2	0.9981	
Final R indices [I>2sigma(I)]	R1 = 0.0808, wR2 = 0.2226	
R indices (all data)	R1 = 0.0866, wR2 = 0.2254	
Largest diff. peak and hole	0.60 and -0.39 e.$Å^{-3}$	

Crystal data and structure refinement for **5e**

CCDC	2440674	
Empirical formula	$C_{16}H_{31}NOSi$	
Formula weight	281.51	
Temperature	100 K	
Wavelength	1.54184 Å	
Crystal system / Space group	Monoclinic / P 2_1/c	
Unit cell dimensions	a = 14.6025(3) Å	a = 90°.
	b = 9.4979(2) Å	b = 104.989(2)°.
	c = 13.0686(3) Å	g = 90°.
Volume	1750.85(7) $Å^3$	
Z	4	
Density (calculated)	1.068 Mg/m^3	
Absorption coefficient	1.121 mm^{-1}	
F(000)	624.000	
Crystal size	0.10 x 0.05 x 0.03 mm^3	
Theta range for data collection	3.133 to 76.295°.	
Index ranges	-18<=h<=18, -11<=k<=11, -16<=l<=16	
Reflections collected	72238	
Independent reflections	3617 [R(int) = 0.049]	
Completeness to theta = 74.769°	99.5 %	
Absorption correction	Semi-empirical from equivalents	
Max. and min. transmission	0.97 and 0.84	
Refinement method	Full-matrix least-squares on F^2	
Data / restraints / parameters	3616 / 228 / 185	
Goodness-of-fit on F^2	0.9975	
Final R indices [I>2sigma(I)]	R1 = 0.0686, wR2 = 0.1725	
R indices (all data)	R1 = 0.0742, wR2 = 0.1767	
Largest diff. peak and hole	0.94 and -0.93 e.$Å^{-3}$	

Crystal data and structure refinement for (+)-**9**

CCDC number	2532699
Empirical Formula	$C_{19}H_{28}BrNO_2$
Formula weight	382.34
Temperature	150 K
Wavelength	λ = 1.54180 Å (Cu Kα)
Crystal System	Orthorhombic
Space Group	P212121
Unit Cell Dimensions	a = 6.36000(15)Å α = 90°
	b = 12.0451(3) Å β = 90°
	c = 24.6018(5) Å γ = 90°
Volume	1884.66(7)$Å^3$
Z	4
Density (Calculated)	1.347 Mg m^{-3}
Absorption Coefficient	3.037 mm^{-1}
Crystal Size	0.13 x 0.17 x 0.17 mm^3
Theta range for data collection	3.593 to 76.284°
Index Ranges	-7<=h<=5, -13<=k<=15, 30<=l<=30
Reflections Collected	9796
Independent Reflections	3871 [R(int) = 0.022]
Completeness to theta = 74.758°	99.7%
Absorption Correction	multi-scan
Refinement method	Full-matrix least-squares on F^2
Goodness-of-fit on F2	1.0065
Final R indices [I>2σ(I)]	R1 = 0.0256, wR2 = 0.0641
R indices (all data)	R1 = 0.0359, wR2 = 0.0901
Flack x parameter	-0.041(13)

11 References

(1) Semeno, V. V.; Vasylchenko, V. O.; Vashchenko, B. V.; Lutsenko, D. O.; Iminov, R. T.; Volovenko, O. B.; Grygorenko, O. O. Building the Housane: Diastereoselective Synthesis and Characterization of Bicyclo[2.1.0]pentane Carboxylic Acids. *The Journal of Organic Chemistry* **2020**, *85* (4), 2321–2337. DOI: 10.1021/acs.joc.9b03044.

(2) Abbott, J. R.; Allais, C.; Roush, W. R. Preparation of crystalline (diisopinocampheyl) borane. *Organic Syntheses* **2015**, *92*, 26–37.

(3) Calais, J.-L. Density-functional theory of atoms and molecules. R.G. Parr and W. Yang, Oxford University Press, New York, Oxford, 1989. IX + 333 pp. Price £45.00. *International Journal of Quantum Chemistry* **1993**, *47* (1), 101–101. DOI: https://doi.org/10.1002/qua.560470107.

(4) Gaussian 16, Rev. C.01; Gaussian, Inc.: Wallingford CT, 2016. (accessed).

(5) Spek, A. Single-crystal structure validation with the program PLATON. *Journal of Applied Crystallography* **2003**, *36* (1), 7–13. DOI: doi:10.1107/S0021889802022112.

(6) Palatinus, L.; Chapuis, G. SUPERFLIP - a computer program for the solution of crystal structures by charge flipping in arbitrary dimensions. *Journal of Applied Crystallography* **2007**, *40* (4), 786–790. DOI: doi:10.1107/S0021889807029238.

(7) Parois, P.; Cooper, R. I.; Thompson, A. L. Crystal structures of increasingly large molecules: meeting the challenges with CRYSTALS software. *Chemistry Central Journal* **2015**, *9* (1), 30. DOI: 10.1186/s13065-015-0105-4.

(8) Cooper, R. I.; Thompson, A. L.; Watkin, D. J. CRYSTALS enhancements: dealing with hydrogen atoms in refinement. *Journal of Applied Crystallography* **2010**, *43* (5 Part 1), 1100–1107. DOI: doi:10.1107/S0021889810025598.
